# Optimizing the Antibiotic Potency and Metabolic Stability of Pyridomycin Using a Semisynthetic Approach

**DOI:** 10.1021/acs.jmedchem.5c02409

**Published:** 2026-01-27

**Authors:** Katherine Valderrama, Oliver Horlacher, Gabriel Publicola, Patrick Eisenring, Maryline Kienle, Samira Boarbi, Mehdi Kiass, Jana Korduláková, Jonathan Chatagnon, Catherine Piveteau, Florence Leroux, Karin Savková, Monika Záhorszká, Francois-Xavier Cantrelle, Christian Lherbet, Lionel Mourey, Katarína Mikušová, Vanessa Mathys, Reiner Aichholz, Laurent Maveyraud, Karl-Heinz Altmann, Ruben C. Hartkoorn

**Affiliations:** † Univ. Lille, CNRS, Inserm, CHU Lille, Institut Pasteur Lille, U1019 - UMR 9017 - CIIL - Center for Infection and Immunity of Lille, F-59000 Lille, France; ‡ Department of Chemistry and Applied Biosciences, Institute of Pharmaceutical Sciences, 31064 ETH Zürich, 8093 Zurich, Switzerland; § 27091 Univ. Toulouse, CNRS, IPBS, 31077 Toulouse, France; ∥ Unit “Tuberculosis & Mycobacteria”, Human Bacterial Diseases Service, Infectious Diseases in Humans, 1050 Brussels, Belgium; ⊥ Faculty of Natural Sciences, Department of Biochemistry, 164047 Comenius University in Bratislava, Ilkovičova 6, Mlynská dolina, 842 15 Bratislava, Slovakia; # Univ. Lille, Inserm, Institut Pasteur de Lille, U1177 - Drugs and Molecules for Living Systems, F-59000 Lille, France; ∇ CNRS, EMR9002 BSI Integrative Structural Biology, 59000 Lille, France; ○ Univ. Lille, Inserm, CHU Lille, Institut Pasteur de Lille, U1167 - RID-AGE - Risk Factors and Molecular Determinants of Aging-Related Diseases, F-59000 Lille, France; ◆ Synthèse et Physico-Chimie de Molécules d’Intérêt Biologique (LSPCMIB), UMR 5068, CNRS, Université Toulouse (UT), 31062 Toulouse, France; ¶ 98560 PK Sciences, Novartis Institutes for BioMedical Research, 4002 Basel, Switzerland

## Abstract

Pyridomycin is a natural product with potent activity against *Mycobacterium tuberculosis* (*Mtb*), acting through direct inhibition of the fatty acid synthesis enzyme InhA. As a direct inhibitor, pyridomycin maintains activity on *Mtb* strains resistant to the InhA targeting prodrugs isoniazid and ethionamide. Evaluation of the drug-like properties of pyridomycin, however, found it to have poor *in vitro* metabolic stability, thus limiting its drug development potential. To address this limitation, semisynthetic derivatives were generated by replacing the metabolically labile hydroxypicolinic acid group with alternative (hetero)­aromatic moieties, identifying several derivatives with improved *in vitro* metabolic stability and with comparable or even enhanced antibacterial activity. Pharmacokinetic studies in mice, however, revealed that these gains did not reduce systemic clearance *in vivo*, and neither pyridomycin nor its derivatives were effective in a murine pulmonary tuberculosis model. Overall, semisynthesis yielded more potent, P450-stable analogs, but the improvements were insufficient to provide measurable *in vivo* efficacy.

## Graphical abstract

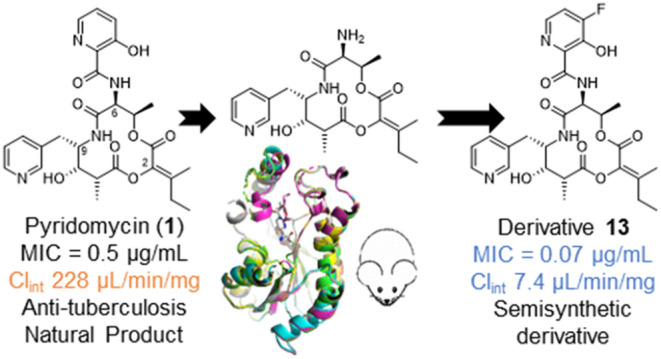

## Introduction

With more than 450,000 new cases of multidrug-resistant tuberculosis annually worldwide and limited therapeutic options to treat infected patients, the causative bacterium *Mycobacterium tuberculosis* (*Mtb*) is considered a critical priority pathogen by the WHO for novel antibiotics research and development. One major therapeutic target in tuberculosis treatment is the ACP-enoyl reductase InhA, a critical enzyme targeted by the active metabolite of the pro-drug antibiotic isoniazid. Clinical resistance to isoniazid is predominantly caused by mutations in its activating enzyme, the catalase KatG, which prevents activation of isoniazid into a bioactive isoniazid-NAD^+^ adduct. Due to the importance of InhA as a drug target, many efforts have been undertaken to develop direct inhibitors of InhA that do not require bioactivation and are not impacted by prevalent *katG* mutations.

As a testament to the efforts made to develop direct InhA inhibitors, numerous inhibitor classes have been discovered and characterized, including diaryl ethers,^1–4^ pyrazoles,^5^ sulfonamides,^6^ arylamides,^7^ thiadiazoles,^8^ diazaborines,^9^ pyridones,^10^ as well as the natural product pyridomycin (**1**).^11^ Pyridomycin (**1**), which acts as a competitive inhibitor of NADH binding to InhA,^11^ is naturally produced by both *Streptomyces pyridomyceticus* ^12,13^ and *Dactylosporangium fulvum*.^14^ It has a narrow antibiotic spectrum, with particularly potent activity against *Mtb*.^11^

A cocrystal structure of InhA and pyridomycin (**1**) revealed the antibiotic to bind to a unique pocket between the NADH and lipid substrate binding site.^15^ To date, a number of pyridomycin analogs have been synthesized through *de novo* chemical synthesis,^16,17^ though, as is often the case with natural products, none of these have shown improved potency. Further development of pyridomycin (**1**) as an antituberculosis drug will require the generation of analogs with better drug-like properties and improved potency compared to the natural product.

In this work, we demonstrate that the drug-like properties of pyridomycin (**1**) are compromised by its very low metabolic stability, which poses a major barrier for its potential development. With the aim of addressing this metabolic liability, the major pyridomycin metabolites were first identified. A semisynthetic approach was then employed to generate pyridomycin derivatives with improved metabolic stability and with superior on-target *in vitro* potency. The interaction of several pyridomycin derivatives with InhA was investigated by X-ray crystallography. Finally, the murine pharmacokinetics and *in vivo* efficacy of pyridomycin and derivatives were evaluated in *Mtb*-infected mice.

## Results

### High Metabolic Instability of Pyridomycin and Identification of Major Metabolites

Using both human and mouse liver microsomes (HLM and MLM, respectively), pyridomycin (**1**) was found to be rapidly metabolized by hydroxylation, with LC-MS analysis detecting the appearance of multiple mono-oxidized (*m*/*z* increase of +16) and doubly oxidized products (*m*/*z* increase of +32) (no glucuronidation was observed). Fragmentation of the six identified mono-oxidized pyridomycin metabolites (m1-6, Figure 1A) by LC-MS/MS allowed the assignment of the different sites of oxidation to 3 regions of the molecule, namely the C2-*iso*-butylidene moiety (four metabolites, m1, m2, m3, m5, together around 53% of oxidized metabolites), the northern 3-hydroxypicolinoyl group (m4, 37% of oxidized metabolites) and N-oxidation of the pyridine ring linked to C9 (m6, 10% of oxidized metabolites). The relative proportions of metabolites detected in HLM and MLM were similar.

**Figure 1:**
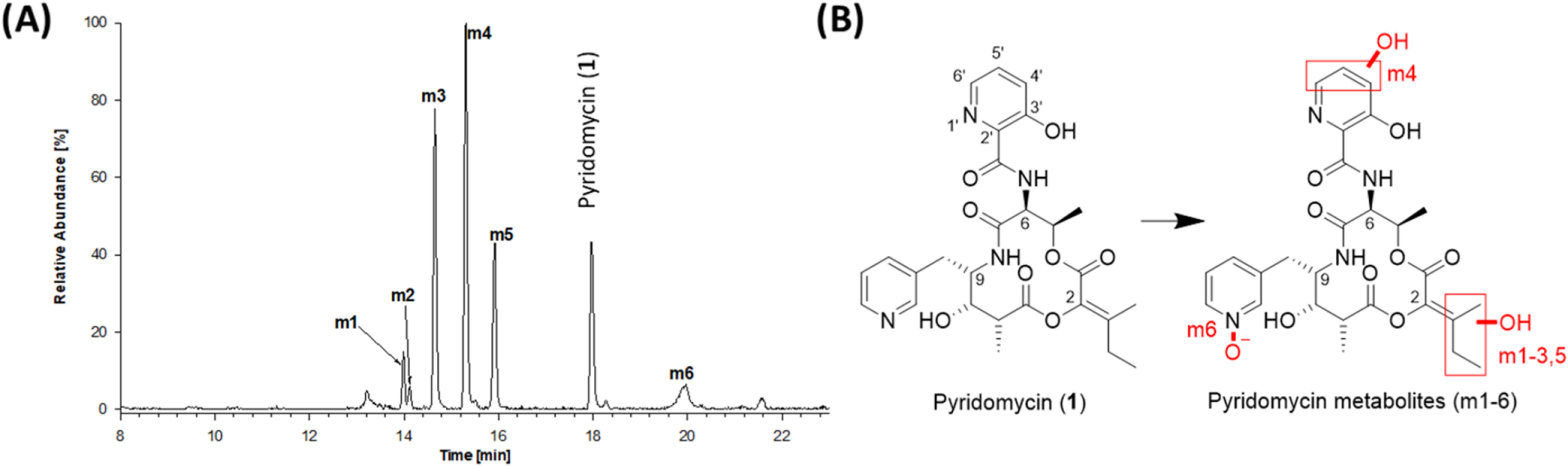
Identification of pyridomycin metabolites. (A) Selected ion chromatogram (SIC) following the abundance of pyridomycin (*m*/*z* = 541 (MH^+^)) and the hydroxylated pyridomycin metabolites m1-m6 (*m*/*z* = 557 (MH^+^)) identified following a 60 min incubation of 5 μM pyridomycin with human liver microsomes (HLM), and a NADPH regenerating system. Unassigned peaks were unrelated to pyridomycin. (B) Predicted regions of oxidation/hydroxylation in pyridomycin metabolites m1-m6 based on MS^n^ analysis.

### Synthesis of Pyridomycin Derivatives by Semisynthesis

The susceptibility of the C2-*iso*-butylidene group and the 3-hydroxypicolinic acid moiety to metabolic hydroxylation indicated that appropriate modification of these substructures should lead to pyridomycin analogs with enhanced metabolic stability over the natural product. As pyridomycin derivatives with modifications at the C2 position are broadly accessible only via complex chemical synthesis,^17^ priority was given to investigating replacements of the 3-hydroxypicolinic acid moiety, which we surmised would be accessible by semisynthesis from natural pyridomycin (**1**). Thus, based on previous work by Barrière et al. on pristinamycins IA/IB and virginiamycin S,^18^ we hypothesized that the amide bond between 3-hydroxypicolinic acid and the C6-amino group on the pyridomycin macrocycle could be reductively cleaved with Zn/HCl; the free amino group would then be acylated with various carboxylic acids to generate pyridomycin derivatives. In the event, treatment of **1** with Zn in aqueous HCl at 0 °C gave the desired amine **2** in 60–70% yield (Scheme 1). Semisynthetic derivatives **3**–**15** with the 3-hydroxypicolinic acid (HPA) part replaced by other aromatic moieties were then successfully generated through HATU-mediated coupling with the respective carboxylic acids (Scheme 1). Derivatives **3**–**15** allowed us to confirm the important pyridomycin-InhA binding interactions and to investigate the basic structure–activity relationship around the HPA moiety with regard to antibacterial potency and metabolic stability.

**Scheme 1:**
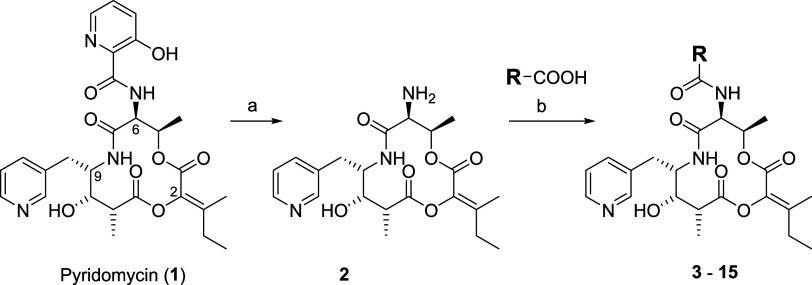
Reagents and Conditions: (a) Zn, aq. HCl, H_2_O, 0 °C, 45 min, 60%, (b) HATU, DIEA, DMF, rt, 18 h, 10-50%^a^

a For individual structures of pyridomycin derivatives, see Table 1.

### Antimycobacterial Potency and On-Target Activity of Pyridomycin Derivatives

The antimycobacterial potency of pyridomycin derivatives **3**-**15** was first assessed on wild-type *Mtb* (H37Rv) using the resazurin microtiter assay (Table 1). In addition to the wild-type strain, antibiotic activity was also determined on a pyridomycin-resistant H37Rv isolate with a mutation in InhA (M161L),^19^ and an isogenic *Mtb* strain that overproduces the pyridomycin target InhA (H37Rv::pMV*inhA* ^11^) (Table 1), both with the aim of evaluating the on-target activity of derivatives.

**Table 1:**
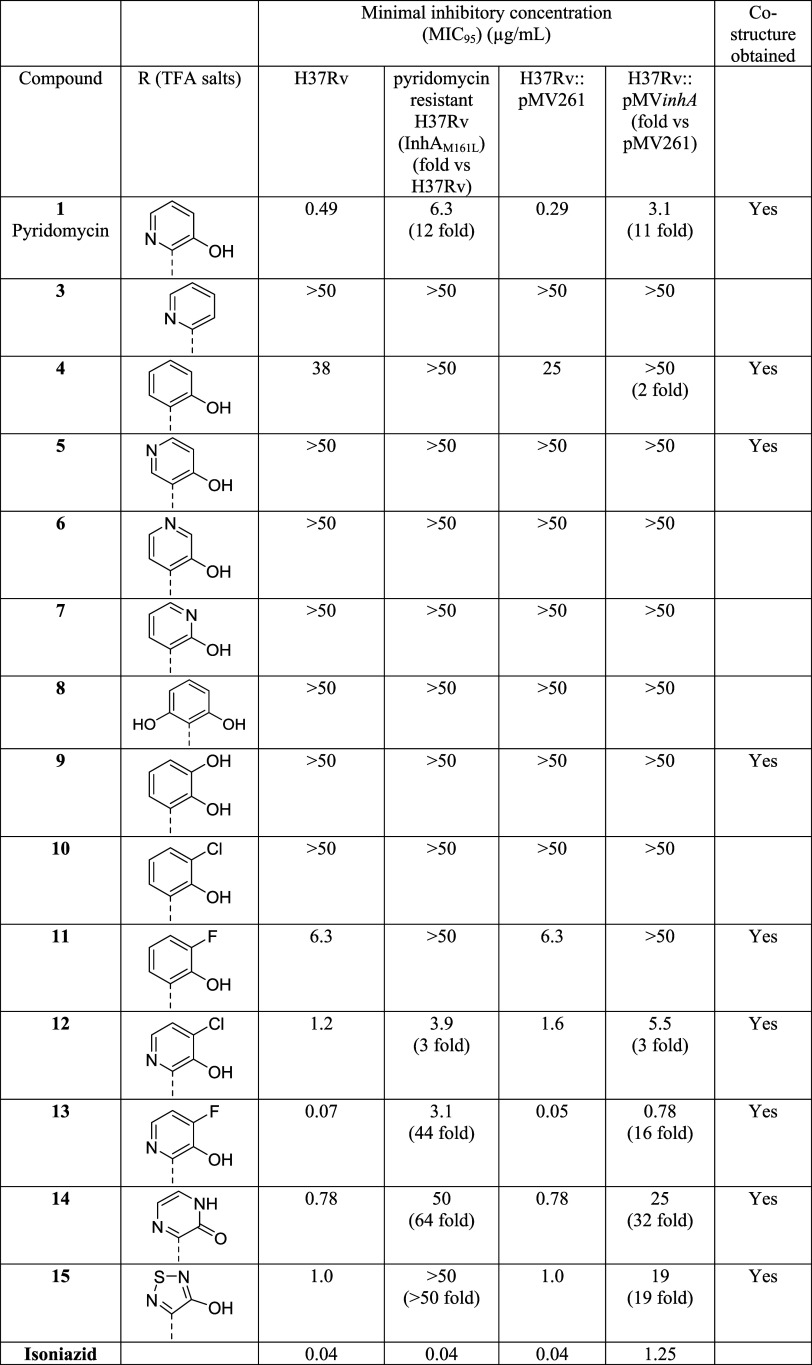
Summary of the Antimycobacterial Activity of Pyridomycin and Semisynthetic Derivatives on Wild-Type H37Rv, A Selected Pyridomycin-Resistant Mutant Carrying an M161L Mutation in InhA, H37Rv Vector Control pMV261, and H37Rv Overproducing InhA (H37Rv::pMV*inhA*)­^a^

a Antibiotic activity is presented as the concentration of compound (μg/mL) needed to prevent at least 95% resazurin turnover (MIC_95_) in the resazurin reduction microplate assay. Data are the average of two biological replicates. The last column indicates whether a co-structure of the inhibitor with InhA was obtained.

The structure–activity relationship data of pyridomycin derivatives **2–7** were in line with pyridomycin (**1**)-InhA binding interactions previously identified by X-ray crystallography (pdb: 4BII and 4BGE).^11^ These structural data showed hydrogen bonding interactions between the HPA hydroxy group and the side chain of Lys-165 of InhA, while the HPA nitrogen forms a hydrogen bond with a water molecule in the binding pocket. In agreement with these findings, removal of the HPA hydroxy group in derivative **3** rendered the molecule inactive, whereas substituting the HPA nitrogen by a CH group in derivative **4** greatly reduced antimycobacterial activity (Table 1). Similarly, a complete loss of antimycobacterial activity was also observed when the nitrogen in the pyridine ring was moved from the *ortho* position (relative to the carboxamide moiety) to either of the two *meta* positions (cpds. **5** and **7**) or the *para* position (cpd. **6**).

In an attempt to improve the potency of the des-aza derivative **4**, a hydroxy group was introduced at position 6 of the resorcylic acid moiety (compound **8**), with the aim of displacing the binding pocket water molecule that the HPA nitrogen interacts with, and thus gain affinity; however, derivative **8** proved to be inactive. In an alternative approach, it was also postulated that binding of the HPA moiety to InhA may be enhanced by the addition of a proximal hydroxy group that would provide an additional interaction with the Lys-165 side chain. To investigate this, catechol derivative **9** was generated, but this derivative was also found to be inactive. It was thus considered futile to generate the corresponding 3,4-dihydroxypyridine-2-carboxylic acid derivative.

The structure of the InhA-pyridomycin complex suggested that there might be some limited space around the six-membered HPA ring (particularly the 3-position) that could potentially be exploited with small substituent groups. To explore this space, we prepared derivatives **9–11**, which carry an additional substituent at the 3-position of the resorcylic acid moiety in derivative **4**. The latter was chosen as the basis for the investigation of additional modifications, as the requisite carboxylic acids were more readily available than the corresponding HPA derivatives and **4** had retained some antimycobacterial activity (though greatly reduced). As shown by the data in Table 1, hydroxylation (derivative **9**) or chlorination (derivative **10**) of the 3-position in the resorcylic acid moiety in **4** only led to a further reduction in antimycobacterial activity. In marked contrast, fluorination (derivative **11**) resulted in a 4-fold improvement in potency (Table 1). In addition, the activity of compound **11** was greatly impacted by the InhA­(M161L) mutation and by *inhA* overexpression, suggesting that the activity was due to InhA inhibition (Table 1). This finding suggested that introducing a fluorine substituent at position 4 of the HPA moiety in pyridomycin (**1**) might also lead to enhanced potency, which led us to prepare the corresponding derivative **13**. Gratifyingly, by analogy to derivative **11**, **13** showed a marked 7-fold improvement in antimycobacterial activity over natural pyridomycin (**1**). As for pyridomycin (**1**), both *inhA* overexpression and the InhA­(M161L) mutation caused significant resistance to **13**, confirming the on-target activity. Based on these observations, the chlorinated pyridomycin derivative **12** was synthesized, even though the corresponding chloro resorcylic acid-derived derivative **10** had been found to be inactive. Interestingly, derivative **12** exhibited anti-Mtb activity that was only 2-fold lower than that of natural pyridomycin (**1**); however, the InhA­(M161L) mutation and *inhA* overexpression had little impact on the antimycobacterial activity of **12**. Despite this, **12** was shown to prevent mycolic acid production in *Mtb* (Supporting Data) and structural biology confirmed the binding of **12** to the pyridomycin binding pocket of InhA. Together, these data imply that **12** targets InhA in *Mtb* but may also have an additional off-target activity. In addition to the modification described above, we also investigated the effect of incorporating a second nitrogen in the six-membered ring by replacing HPA by 2-pyridazine-3-carboxylic acid, resulting in derivative **14**. The latter was found to exhibit on-target activity similar to that of pyridomycin itself (Table 1). Finally, replacement of the 6-membered HPA moiety by a 5-membered 4-hydroxy-1,2,5-thiadiazole-3-carboxylic acid group (derivative **15**) also resulted in on-target anti-*Mtb* activity similar to natural pyridomycin (**1**) (Table 1, Figure S1). Overall, our semisynthesis approach allowed us to access a series of pyridomycin derivatives with modifications in the natural HPA moiety. Among these compounds, derivatives **12–15** showed similar or even improved anti-*Mtb* activity compared to natural pyridomycin (**1**), with the activity of **13–15** clearly remaining on-target. Finally, we note that the SAR derived here for HPA modifications/replacements in pyridomycin derivatives **3, 4, 12**, and **14** closely tracks observations for the corresponding fully synthetic 2-cyclohexyl-dihydropyridomycin variants (Table S1, compounds **S3–S6**).

### Structural Biology of InhA in Complex with Pyridomycin and Derivatives

High-resolution crystal structures were obtained for InhA in complex with pyridomycin (**1**) and pyridomycin derivatives **4**, **5**, **9**, and **11–15** (Figures 2 and S2, Table S2). Although structures of InhA with pyridomycin (and derivatives)^15^ were previously obtained by soaking InhA crystals containing the NADH cofactor, the structures described herein were obtained with an *apo*-InhA. The resulting InhA-inhibitor complexes were found in two different crystal forms, specifically spacegroup C2, for structures containing pyridomycin (**1**) and derivatives **4**, **5**, **9**, **12**, and **13** (6 molecules per asymmetric unit, with chains C, D, E and F forming the typical InhA tetramer and chains A and B forming a tetramer with symmetry related copies), and spacegroup P2_1_ for structures with derivatives **11**, **14**, and **15** (with 4 molecules forming the typical InhA tetramer in the asymmetric unit). In addition to differences in the crystal form, the costructures showed some differences in inhibitor occupancy per chain and in some cases showed differences in the conformation of the substrate binding loop that lines the inhibitor binding pocket (summarized in Figures 4–4). Such variability of crystal form, inhibitor occupancy, and substrate binding loop conformation has previously been observed with InhA and has been thoroughly discussed.^20^

**Figure 2:**
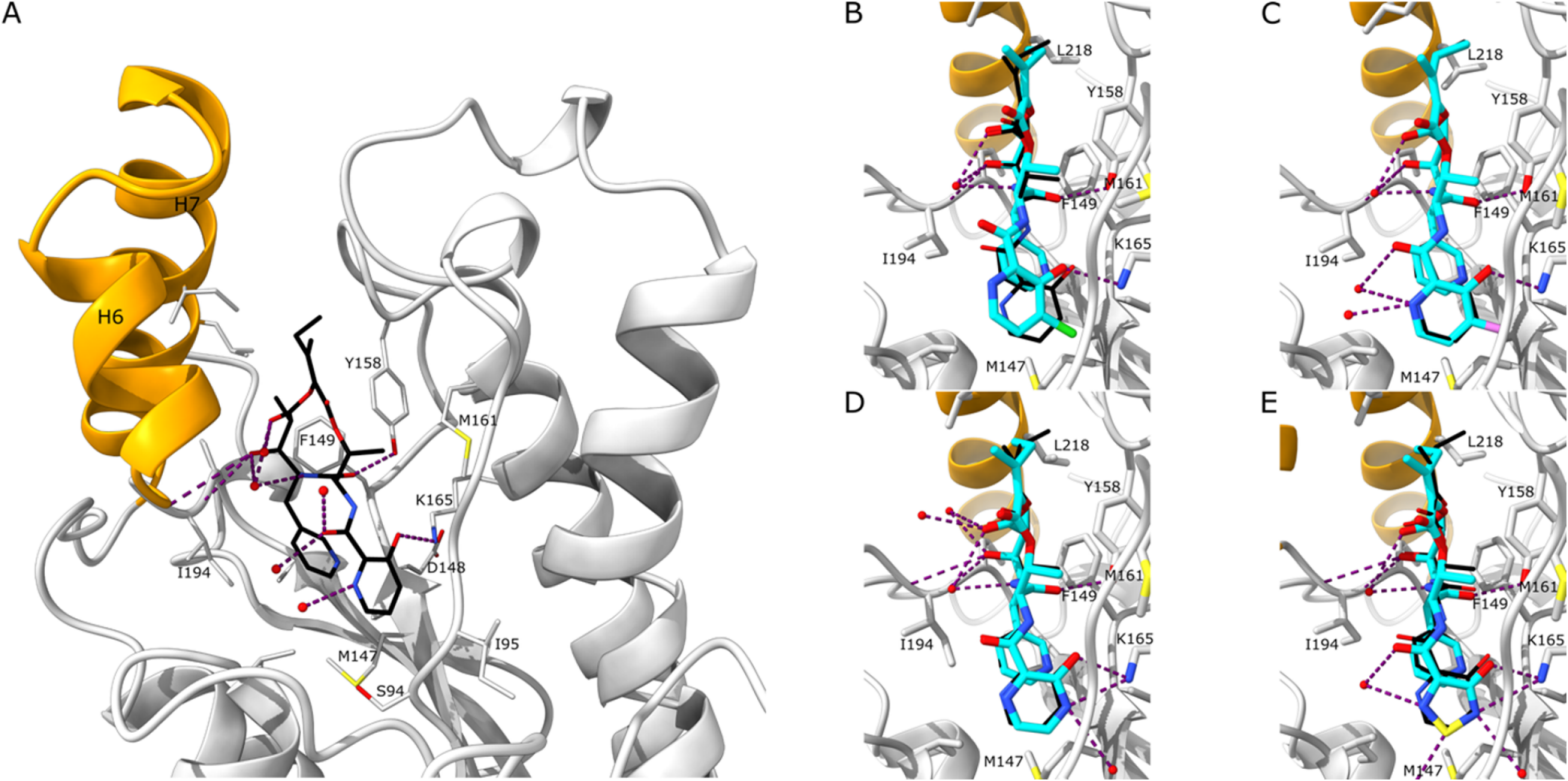
(A) View of the interaction of pyridomycin (black sticks) with InhA (gray), showing all side chains within 5 Å of pyridomycin (**1**) as sticks (PDB: 9RJG). Possible hydrogen bonds of pyridomycin (**1**) with InhA residues and with water molecules are shown as violet dotted lines. Helices H6 and H7 that form the substrate binding loop are represented in orange. (B–E) View of the interaction of pyridomycin derivatives (cyan sticks, B: compound **12**, PDB: 9RJL, C: compound **13**, PDB: 9RJM, D: compound **14**, PDB: 9RJN, and E: compound **15**, PDB: 9RJP) with InhA. Pyridomycin (**1**) is superimposed as black sticks. Possible hydrogen bonds of the pyridomycin derivatives with InhA residues and with water molecules are shown as violet dotted lines.

The InhA-pyridomycin crystal structure (PDB: 9RJG) revealed pyridomycin bound in all six InhA chains of the asymmetric unit, occupying nearly identical positions to those reported previously.^15^ As expected, cocrystal structures of InhA with eight pyridomycin derivatives showed the compounds bound in largely overlapping positions (Figures 2 and S2), with key hydrogen bond interactions of the macrolactone ring with Tyr-158, Thr-196 (through a water molecule), and Ile-194 conserved across all derivatives. Of note, two costructures were obtained for pyridomycin derivatives that showed no antibacterial activity. These include compound **5** (PDB: 9RJI), where the aromatic nitrogen of the introduced 4-hydroxynicotinic acid group was found to be within hydrogen bonding distance from the side chain of Ser-94 (Figure S2C). The other compound is **9** (PDB: 9RJJ), where the hydroxy groups of the 2,3-dihydroxy benzoyl moiety are both in hydrogen bonding distance to Lys-165 (Figure S3A). With respect to the active pyridomycin derivatives **12–15** (Figure 2), the introduced aromatic nitrogen atom and hydroxy groups largely overlap the corresponding positions of the hydroxypicolinic acid moiety in pyridomycin (**1**), forming hydrogen bond interactions with Lys-165 (Figure 2 **B–E**). Despite the similarity, it was noted that for the InhA-**12** costructure (Figure 2B, PDB: 9RJL), the introduction of the chlorine atom caused a significant tilt in the aromatic group, suggesting that this more bulky introduction was less well tolerated, and this may help explain why the resistance pattern of **12** (Table 1) deviated from that of the other derivatives.

### *In Vitro* ADME Properties of Pyridomycin (**1**) and Pyridomycin Derivatives

To evaluate if the more potent pyridomycin derivatives **12–15** had improved ADME properties, the *in vitro* plasma stability and metabolic stability in mouse liver microsomes (MLM) were determined. First, despite the presence of two ester bonds in their core macrocycle, all four compounds were found to have good plasma stability (>80% parent compound remaining after 6 h, Table 2). *In vitro* metabolic stability studies in MLM confirmed the high intrinsic clearance of pyridomycin (**1**), and this metabolism was likely mediated by cytochrome P450 oxidases, as clearance was prevented by the addition of the nonspecific cytochrome P450 inhibitor 1-aminobenzotriazole (ABT) (Table 2). Satisfyingly, the *in vitro* metabolic stability of derivatives **12–15** was revealed to be significantly higher than that of pyridomycin (**1**) (Table 2), thus demonstrating that appropriate modification/replacement of the metabolically labile HPA moiety could prevent the metabolism of the antibiotic.

**Table 2:** *In Vitro* Plasma and Mouse Liver Microsomes (MLM) Stability of Pyridomycin (**1**) and Pyridomycin Derivatives **12–15^a^**

		MLM stability expressed as	
compounds	plasma stability (% remaining)^b^	percentage remaining (%)^c^	intrinsic clearance Cl_int_ (μL/min/mg)^d^
pyridomycin (**1**)	84	5.7	228
pyridomycin (**1**) + ABT	95	98	2.1
**12**	98	73	25
**13**	86	91	7.4
**13** + ABT	85	100	0
**14**	95	92	8.1
**15**	100	93	7.4
enalapril	18		
propranolol			152

a The stability of the pyridomycin derivatives was measured by LC-MS/MS and multiple reaction monitoring (MRM) targeting the parent molecule.

b Percentage of compound remaining after 6 h incubation with plasma.

c Percentage of compound remaining after 40 min incubation with MLM.

d Calculated using Cl_int_ = |k|/[microsomes] where k is the first-order degradation constant (i.e., the slope of the logarithm of compound concentration as a function of incubation time) and [microsomes] is the concentration in microsomes expressed in mg/μL. For pyridomycin (**1**) and compound **13**, plasma stability and microsomal stability were also investigated with co-incubation of the cytochrome P450 inhibitor 1-aminobenzotriazole (ABT).

### Murine Pharmacokinetics of Pyridomycin (**1**) and Derivatives **12**–**15**

The pharmacokinetics of pyridomycin (**1**) and derivatives **12–15** was studied in mice following a single 10 mg/kg i.p. dose (Figure 3). In line with its high intrinsic clearance in MLM, pyridomycin (**1**) was found to be eliminated rapidly from murine plasma. When mice were preadministered 50 mg/kg p.o. ABT 2 h before pyridomycin dosing, pyridomycin *C*_max_ increased nearly 8-fold (decreased first pass clearance), systemic clearance decreased (36-fold increase in elimination half-life), and systemic exposure (AUC) increased more than 49-fold (Table 3), demonstrating the important role of hepatic clearance in pyridomycin pharmacokinetics in mice. Surprisingly, derivatives **12–15**, which all had shown improved *in vitro* metabolic stability in MLM, did not show any significant improvement in exposure, with all four compounds being rapidly eliminated. For the most potent derivative **13**, preadministration of ABT was also investigated; however, unlike for pyridomycin, ABT improved systemic exposure only slightly (3-fold increase in *C*_max_ and 4-fold increase in AUC, Table 3). In combination, the *in vitro* and *in vivo* clearance data in mice suggest that the exposure of derivative **13** is not driven by hepatic, cytochrome P450-mediated metabolism/elimination. The stark difference in the drivers of pharmacokinetic elimination between pyridomycin (**1**) and derivative **13** was unexpected and requires further work investigating the potential role of alternative routes of elimination such as renal clearance.

**Figure 3:**
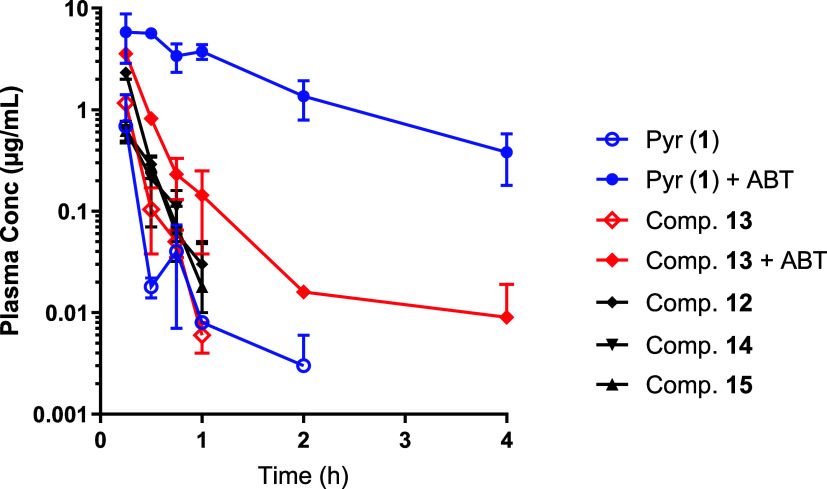
Murine plasma pharmacokinetic profile of pyridomycin (**1**) and pyridomycin derivatives (**12–15**) following 10 mg/kg intraperitoneal administration. For pyridomycin (**1**) and compound **13**, pharmacokinetics was also investigated with coadministration of the cytochrome P450 inhibitor 1-aminobenzotriazole (ABT).

**Table 3:** Murine Pharmacokinetic Parameters of Pyridomycin Derivatives following a 10 mg/kg Intraperitoneal Administration^a^

	**12**	**14**	**15**	**13**	**13 + ABT**	**Pyr (1)**	**Pyr (1) + ABT**
***C***_**max**_ **(μg/mL)**	2.31	0.63	0.62	1.16	3.55	0.69	5.81
***T***_**max**_ **(min)**	15	15	15	15	15	15	15
***t*** 1/2 **(min)** ^b^	5.1	10.4	11.3	4.8	7.3	3.0	54.4
**AUC**_**(0–4)**_ **(h·μg/mL)** ^c^	0.67	0.22	0.25	0.33	1.27	0.19	8.48
**AUC**_**(0‑∞)**_ **(h·μg/mL)** ^c^	0.67	0.27	0.25	0.33	1.28	0.19	9.38

a Compounds were administered as trifluoroacetic acid (TFA) salts. Pyr (**1**) = pyridomycin (**1**); ABT = 1-aminobenzotriazole.

b Apparent half-life (*t*_1/2_) was estimated in GraphPad Prism using a nonlinear regression with one phase decay.

c AUC_(0–4)_ was calculated based on the trapezoidal rule, with extrapolation for AUC_(0‑**∞**)_.

### *In Vivo* Efficacy of Pyridomycin (**1**) and Derivative **13** in H37Rv-Infected Mice

Based on their anti-*Mtb* activity, *in vitro* metabolism, and pharmacokinetic behavior, it was decided to evaluate the *in vivo* efficacy of both pyridomycin (**1**) and derivative **13** in an acute model of *Mtb* lung-infected mice (infected with the luminescent strain H37Rv-lux) alone or in combination with ABT. Treatment of *Mtb*-infected mice was initiated 1 week post infection, and lasted 1 week before evaluating the impact on bacterial load. Treatment with isoniazid (25 mg/kg, po, qd) resulted in the expected decrease in bacterial load (Figure 4). On the other hand, twice-daily dosing with pyridomycin (**1**) or derivative **13** at 25 mg/kg with or without pretreatment with ABT (50 mg/kg) was found not to impact bacterial growth, with results being similar to the vehicle control. Thus, our efforts to improve the *in vivo* exposure of mice to pyridomycin-based *Mtb* inhibitors were insufficient to result in an *in vivo* efficacy.

**Figure 4:**
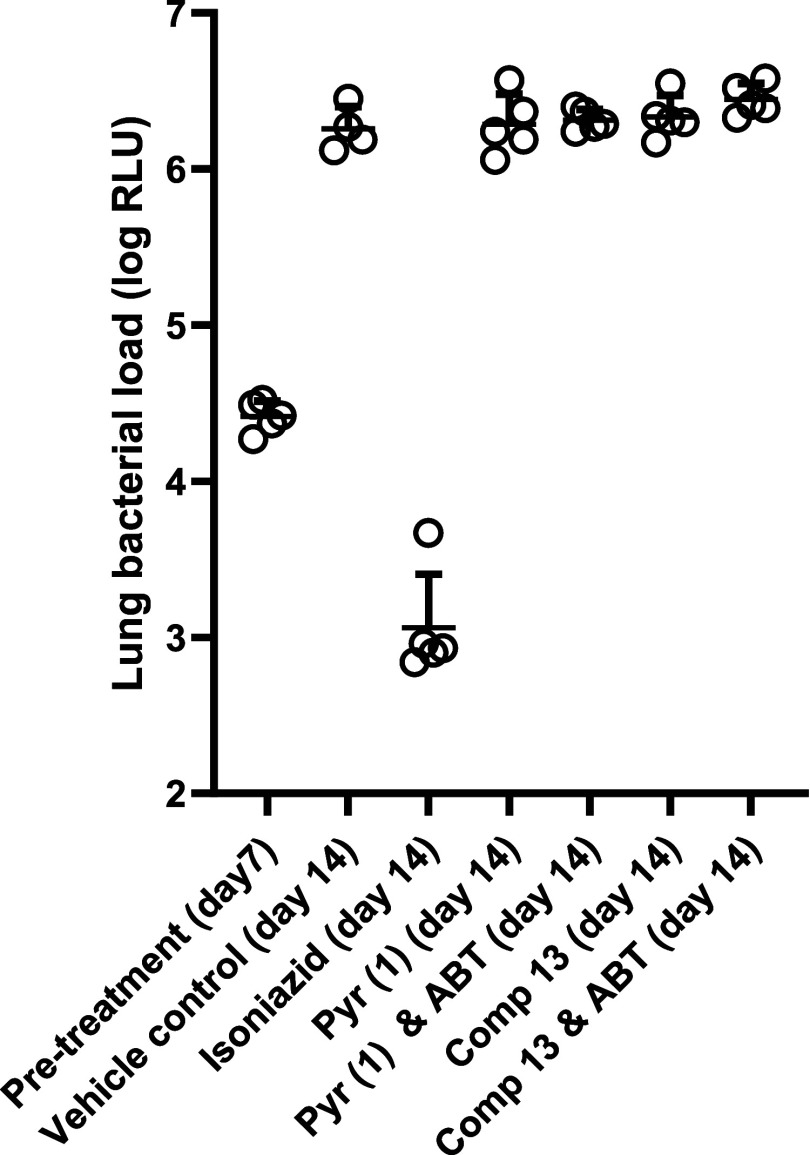
*In vivo* efficacy studies of pyridomycin and derivatives against *Mtb*-infected mice. Mice were infected on day 0 by intratracheal infection. Treatment started at day 7, for 5 days, with twice-daily administration of pyridomycin (**1**) or compound **13** (both 25 mg/kg, i.p., b.i.d.), with or without preadministration of ABT (50 mg/kg p.o., b.i.d.). As a control group, isoniazid treatment was given once daily (25 mg/kg, p.o., q.d.). Bacterial load in the lungs was measured by relative luminescence units (RLU) of the H37Rv-lux strain, 5 mice per group.

## Discussion and Conclusions

Natural products represent a rich source of antibiotics that can often unveil novel mechanisms of antibacterial action. A case in point is the bacterial natural product pyridomycin (**1**), which has been shown to inhibit the enoyl reductase InhA of *Mtb* through a novel mechanism,^11,15^ thereby overcoming current mechanisms of resistance to the antituberculosis drug isoniazid. Unfortunately, the antibiotic potential of natural products is frequently hampered by poor pharmacokinetic properties, such as rapid elimination by hepatic clearance. For these natural products to be developed into potential therapeutic agents, it is critical that derivatives are synthesized with improved drug-like properties.

Here, we have been able to create derivatives of pyridomycin (**1**) with greatly improved metabolic stability by first identifying the HPA moiety as the main site of metabolism in the natural product and then employing a semisynthetic approach to generate derivatives where this site was modified or replaced. In doing so, we found that the introduction of a fluorine substituent at position 4 of the pyridine ring of HPA, (derivative **13**), the substitution of N for C(4)H (derivative **14**), or the complete replacement of the 6-membered pyridine ring by a 5-membered 1,2,5-thiadiazole heterocycle (derivative **15**) all prevented microsomal clearance without loss of antibiotic activity. As the microsomal instability of pyridomycin (**1**) was found to be heavily driven by cytochrome P450-mediated hydroxylation of the HPA moiety, these modifications likely prevent this route of metabolism. In addition, as the corresponding derivatives showed near full *in vitro* metabolic stability, it appears that the modifications also prevented the oxidation of the metabolically labile *iso*-butylidene group, perhaps by altering substrate affinity for specific cytochrome P450s. Unfortunately, the considerable improvements in *in vitro* metabolic stability relative to pyridomycin did not translate into improved *in vivo* pharmacokinetic properties for derivatives **12–15**. In the case of derivative **13**, coadministration of ABT had little impact on its pharmacokinetic profile, suggesting that its route of elimination may be different from that of natural pyridomycin (**1**), with extensive phase II metabolism or renal clearance or both being likely causes.

Finally, twice-daily treatment of *Mtb-*infected mice with pyridomycin (**1**), even upon coadministration of ABT to boost its pharmacokinetic profile, did not result in any significant reduction in bacterial load. The reasons for this finding are unclear at this point but may be related to insufficient exposure of the bacteria to the compound in the lung lining fluid (not measured in this study). Additionally, we have noted that the *in vitro* anti-*Mtb* activity of pyridomycin is 4 to 8-fold lower when Tween 80 was omitted from the culture media,^17^ which makes it challenging to estimate the concentration needed to elicit antituberculosis activity in mice.

This work has demonstrated that semisynthesis is an attractive means to generate pyridomycin derivatives with improved metabolic stability but also enhanced anti-*Mtb* activity. While not sufficient to attain *in vivo* efficacy, this work is a step forward in the development of pyridomycin derivatives with drug-like properties.

## Experimental Section

### Purification of Pyridomycin from *Dactylosporangium fulvum* Culture

Pyridomycin was produced from *Dactylosporangium fulvum* as described previously^11,21^ with small modifications. Briefly, *D. fulvum* was grown in AYM medium (0.54% (w/v) sodium acetate, 0.4% (w/v) yeast extract, and 1% (w/v) malt extract, pH 7.2) for 7 days (28 °C, 220 rpm). Bacteria were pelleted (4000*g*, 30 min) and pyridomycin was first extracted from the supernatant by solid phase extraction using Amberchrom CG300 resin (Sigma) and elution with a gradient of methanol in water (pyridomycin was primarily present in the 60–80% MeOH fraction). Following evaporation of methanol under reduced pressure, pyridomycin was extracted from the aqueous fraction with ethyl acetate. The organic layer was then evaporated under reduced pressure, and pyridomycin (**1**) was purified by preparative HPLC, using an XBRIDGE PST C18 OBD Prep column (130A 5 μm 19 mm × 150 mm) and a mobile phase gradient of 20% to 40% ACN in H_2_O-0.1% TFA over 30 min. Fractions containing pyridomycin (TFA salt) were combined and lyophilized. The identity of pyridomycin (**1**) was confirmed by mass spectrometry and ^1^H NMR spectroscopy. The spectroscopic data were in agreement with those previously reported in the literature.^21,22^

### Metabolite Identification

#### *In Vitro* Experiment with Liver Microsomes

Stock solutions of pyridomycin (**1**) (2 mmol/L) were prepared in DMSO. Alamethicin solution was prepared (0.125 mmol/L) in water. UDPGA solution (24 mmol/L) was prepared in phosphate buffer (100 mM, pH 7.4). *In vitro* metabolites were produced by incubation of pyridomycin (**1**) at 37 °C for up to 60 min with liver microsomes from mouse and human. 3 μL liver microsomes, containing 20 mg protein/mL, were mixed with 417 μL of phosphate buffer, 60 μL of Alamethicin solution, and 60 μL of UDPGA. To this reaction mixture, 3 μL stock solution (2 mM in DMSO) of pyridomycin (**1**) was added and preincubated for 3 min at 37 °C. After preincubation, the final reaction was started by the addition of 60 μL of the NADPH regenerating system, containing isocitrate dehydrogenase (1 U/mL), NADP (1 mmol/L), and isocitrate (5 mmol/L). After 1 h, the reaction was stopped with 600 μL of ice-cold acetonitrile. The reaction mixture was stored at −80 °C. Prior to analysis, reaction mixtures were thawed and centrifuged (10,000*g*, 5 min), after which 100 μL of supernatant was diluted with 400 μL of water. Following a final centrifugation (10,000*g*, 5 min), 5–10 μL was used for HPLC/MS analysis to identify pyridomycin metabolites.

#### Capillary High-Performance Liquid Chromatography–Mass Spectrometry (HPLC/MS)

Capillary HPLC was performed on a system consisting of a Chorus-220 HPLC pump (CTC Analytics, Zwingen, Switzerland), a Hot Dog-5090 column oven (Prolab, Reinach, Switzerland), and an HTS-PAL autosampler with cooled sample stacks (CTC Analytics, Zwingen, Switzerland). Separations were performed on a Reprosil-Pure-C18-AQ HPLC column (150 mm × 0.3 mm inner diameter, 3.0 μm particle size) from Maisch (Ammerbuch-Entringen, Germany). Separations were performed at 45 °C. The flow rate was 4.5 μL/min, and the injection volume was 1 μL per separation. The solvent system used consisted of aqueous ammonium formate (10 mM, with 0.02% TFA, pH 4)/MeCN (95/5, v/v) as solvent A and aqueous ammonium formate (10 mM, with 0.02% TFA, pH 4)/MeCN/MeOH (5/90/5, v/v/v) as solvent B. The metabolites were separated using a linear solvent gradient: 0 min (5% B), 2 min (5% B), 27 min (95% B), and 32 min (95% B, 6.5 μL/min). Re-equilibration of the column was performed at 5% B for 5 min. Prior to analysis, 10 μL of sample was diluted 1/100 (v/v) with water/MeCN (90/10, v/v). Samples were kept in the autosampler at 10 °C. A LTQ XL Orbitrap (Linear Quadrupole 2D Ion Trap/Orbitrap, Thermo Scientific, CA, USA) mass spectrometer was used operating in positive mode with electrospray ionization (ESI). Settings for the mass spectrometer were 12 (arbitrary units) for sheath gas flow rate (N_2_) and a capillary temperature of 275 °C. Auxiliary and sweep gases were not used. The settings for the capillary voltage and tube lens voltage were 45 and 95 V, respectively. Auto gain control (AGC) target settings were 5 × 10^5^ and 1 × 10^4^ for full MS and MS/MS, respectively. The resolution was set to 30,000 in full scan mode. The omnipresent polysiloxane background ion [C_2_H_6_SiO]_6_^+^ with *m*/*z* 445.12003 was used as the external lock mass.

### Semisynthesis of Pyridomycin Derivatives

#### General Chemistry Procedures

NMR spectra for the cpds. **2**–**12** and **15** were recorded on a Bruker Avance III300 spectrometer equipped with a 5 mm BBO (X-1H) probe or a Bruker Avance IIIHD 600 equipped with a 5 mm cryogenic QCI (1*H*/2H/13*C*/15*N*/19F) probe. Spectra were for cpds. **13**, **14**, and **S3**–**S6** were recorded on a Bruker Avance 400 or 500 MHz NMR spectrometer at 300 K. Chemical shifts (δ) are reported in ppm and are referenced to the solvent signal as an internal standard (CD_3_OD δ 3.31 ppm for ^1^H spectra and (CD_3_)_2_SO δ 2.50 ppm for ^1^H spectra). Data are reported as follows: s = singlet, d = doublet, t = triplet, q = quartet, quint = quintet, sext = sextet, m = multiplet, br. = broad signal, and *J* = coupling constant in Hz. The multiplicity of signals is reported based on appearance (i.e., doublet of doublets that are apparent triplets are described as triplets). The ^13^C NMR spectrum of **13** was measured with complete proton decoupling. ^1^H and ^13^C signals were assigned using two-dimensional correlation experiments (COSY, HSQC, and HMBC). The purity of final products submitted for biological testing was determined by analytical RP-HPLC to be ≥ 95%.

Analytical mass spectrometry (non-high resolution) for cpds. **2**–**12** and **15** was performed on a UHPLC-MS system composed of a Ultimate 3000 UHPLC system, coupled with a LCQ Fleet Ion Trap Mass Spectrometer (Thermo Scientific). Chromatographic separation was achieved using an Acquity UPLC Peptide BEH C18 column (300 Å, 1.7 μm, 2.1 mm × 100 mm) using a mobile phase gradient from solvent A (H_2_O, 0.1% TFA) to B (acetonitrile, 0.1% formic acid). For cpds **13**, **14**, and **S2**–**S6**, analytical mass spectrometry was performed by the MS service of the Laboratory of Organic Chemistry (LOC) of the ETH Zürich; HRMS (ESI) using a Bruker Daltonics maxis (UHR-TOF) instrument. High-resolution mass spectrometry (HRMS) of purified compounds was performed by the ARIADNE-ADME platform (Institut Pasteur de Lille, France), using a quadrupole time-of-flight (TOF) LCT Premier XE mass spectrometry machine (Waters).

##### N-((5R,6S,9S,10S,11R,Z)-2-(Butan-2-ylidene)-10-hydroxy-5,11-dimethyl-3,7,12-trioxo-9-(pyridin-3-ylmethyl)-1,4-dioxa-8-azacyclododecan-6-yl)-3-hydroxypicolinamide (**2**)

Pyridomycin (**1**) (300 mg, 0.46 mmol, 1.0 equiv) was suspended in H_2_O (18.0 mL) with conc. HCl (1.44 mL) at 0 °C, and the mixture was stirred for 5 min. Zinc powder (280 mg, 4.3 mmol, 9.3 equiv) was then added in portions, and the mixture was stirred for 35 min at 0 °C until no pyridomycin (**1**) remained (based on TLC and HPLC-MS analysis). The pH was then adjusted to 8.0 by the addition of aq. NaOH (2 M) and the solution was extracted with DCM (twice) and EtOAc (thrice). The combined organic extracts were dried over MgSO_4_, filtered, and concentrated under vacuum. The orange oil was then subjected to flash chromatography (CH_2_Cl_2_: MeOH 9:1), providing **2** at >95% purity (107.2 mg, 46% yield). ^1^H NMR (300 MHz, MeOD-*d*_4_): δ (ppm): 8.47 (dd, *J* = 2.2, 0.74 Hz, 1H), 8.39 (dd, *J* = 4.9, 1.6 Hz, 1H), 7.81 (dt, *J* = 7.9, 1.9 Hz, 1H), 7.38 (ddd, *J* = 7.9, 4.9 Hz, 0.7, 1H), 5.12 (s, 1H), 4.10 (t, *J* = 7.1 Hz, 1H), 3.63 (s, 1H), 3.41 (d, *J* = 6.2 Hz, 1H), 3.06–2.89 (m, 2H), 2.71–2.56 (m, 1H), 2.29–2.15 (m, 4H), 2.10–1.96 (m, 1H), 1.43 (d, *J* = 7.3, 1H), 1.27 (d, *J* = 6.4 Hz, 3H), 1.01 (t, *J* = 7.6 Hz, 3H). HRMS (ESI): *m*/*z* calcd for C_21_H_30_N_3_O_6_ [M + H]+: 420.2129, found 420.2133.

#### General Procedure for the Synthesis of Pyridomycin Derivatives **3**–**12**, **15** through the Coupling of Carboxylic Acids with Free Amine **2**

To a solution of a chosen carboxylic acid (2.2 equiv) and HATU (2.2 equiv) in DMF (concentration acid 0.35 M), DIEA (3.2 equiv) was added slowly. The solution was stirred for 30 min at rt, and a solution of amine **2** (1 equiv) was added. The mixture was stirred for 18 h at rt. After this time, the mixture was diluted with EtOAc and sat. NaHCO_3_. The aqueous phase was extracted with EtOAc and the combined organic extracts were dried over MgSO_4_, filtered, and concentrated in vacuum. The residue was purified by preparative HPLC, using a XBRIDGE PST C18 OBD Prep column (130A 5 μm 19 mm × 150 mm) and a mobile phase gradient of 8% to 40% ACN in H_2_O-0.1% TFA over 40 min. The purified products were thus obtained as TFA salts.

##### N-((5R,6S,9S,10S,11R,Z)-2-(Butan-2-ylidene)-10-hydroxy-5,11-dimethyl-3,7,12-trioxo-9-(pyridin-3-ylmethyl)-1,4-dioxa-8-azacyclododecan-6-yl)­picolinamide (**3**)

Compound **3** was prepared through the coupling of picolinic acid (Sigma) with amine **2** according to the general procedure, with a 79% yield. ^1^H NMR (600 MHz, (CD_3_)_2_SO): δ 8.67 (doublet br, *J* = 4.5 Hz, 1H), 8.59 (br, 1H), 8.44 (d, *J* = 4.9 Hz, 1H), 8.21 (br,1H), 8.08 (doublet br, *J* = 7.7 Hz, 1H), 8.06 – 7.98 (br, 2H), 7.85 (br, 1H), 7.65 (ddd, *J* = 7.5, 4.7, 1.4 Hz, 1H), 7.46 (t, *J* = 6.1 Hz, 1H), 5.18 (br, 1H), 4.73 (br, 1H), 4.23–4.15 (br, 1H), 3.73 (t, *J* = 2.1 Hz, 1H), 3.04 (dd, *J* = 13.6, 6.0 Hz, 1H), 2.93 (dd, *J* = 13.5, 8.5 Hz, 1H), 2.72 – 2.64 (br, 1H), 2.17 (br, 6H), 1.40 (d, *J* = 7.2 Hz, 3H), 1.14 (d, *J* = 5 Hz, 3H), 0.99 (t, *J* = 7.6 Hz, 3H). HRMS (ESI): *m*/*z* calcd C_27_H_33_N_4_O_7_ [M + H]^+^: 525.2349, found: 525.2327.

##### N-((5R,6S,9S,10S,11R,Z)-2-(Butan-2-ylidene)-10-hydroxy-5,11-dimethyl-3,7,12-trioxo-9-(pyridin-3-ylmethyl)-1,4-dioxa-8-azacyclododecan-6-yl)-2-hydroxybenzamide (**4**)

Compound **4** was prepared through the coupling of 2-hydroxybenzoic acid (Sigma) with amine **2** according to the general procedure, with a 33% yield. ^1^H NMR (600 MHz, (CD_3_)_2_SO): δ 8.60 (s, 1H), 8.56 (d, *J* = 7.0 Hz, 1H), 8.47 (d, *J* = 4.0 Hz, 1H), 8.01 (d, *J* = 7.8 Hz, 1H), 7.91 (dd, *J* = 7.9, 1.6 Hz, 1H), 7.75 (br, 1H), 7.47 (t, *J* = 5.8 Hz, 1H), 7.38 (ddd, *J* = 8, 7.03, 1.7 Hz, 1H) or (m), 1H, 6.96 (d, *J* = 8.2 Hz, 1H), 6.93 (t, *J* = 7.5 Hz, 1H) or (m), 5.17 (br, 1H), 4.74 (br, 1H), 4.19–4.14 (br, 1H), 3.71 (br,1H), 3.04 (dd, *J* = 13.6, 6.1 Hz, 1H), 2.94 (dd, *J* = 13.6, 8.3 Hz, 1H), 2.70 – 2.63 (br, 1H), 2.17–1.97 (br, 6H), 1.39 (d, *J* = 7.3 Hz, 3H), 1.17 (br, 3H), 0.99 (t, *J* = 7.6 Hz, 3H). HRMS (ESI): *m*/*z* calcd C_28_H_34_N_3_O_8_ [M + H]^+^: 540.2346, found: 540.2338.

##### N-((5R,6S,9S,10S,11R,Z)-2-(Butan-2-ylidene)-10-hydroxy-5,11-dimethyl-3,7,12-trioxo-9-(pyridin-3-ylmethyl)-1,4-dioxa-8-azacyclododecan-6-yl)-4-hydroxynicotinamide (**5**)

Compound **5** was prepared through the coupling of 4-hydroxynicotinic acid (Ambinter) with amine **2** according to the general procedure, with a 53% yield. ^1^H NMR (600 MHz, (CD_3_)_2_SO): δ 10.53 (d, *J* = 7.6 Hz, 1H), 8.59 (s, 1H), 8.48 (d, *J* = 4.8 Hz, 1H), 8.42 (d, *J* = 1.7 Hz, 1H), 8.05 (d, *J* = 8.1 Hz, 1H), 7.77 (dd, *J* = 7.2, 1.8 Hz, 1H), 7.67 (d, *J* = 6.0 Hz, 1H), 7.53 (dd, *J* = 8.0, 5.3 Hz, 1H), 6.40 (d, *J* = 7.3 Hz, 1H), 5.13 (s, 1H), 4.66 (s, 1H), 4.20–4.13 (m, 1H), 3.69 (s, 1H), 3.04 (dd, *J* = 13.6, 6.2 Hz, 1H), 2.93 (dd, *J* = 13.6, 8.3 Hz, 1H), 2.66 (q, *J* = 6.8 Hz, 1H), 2.19–2.05 (m, 6H), 1.38 (d, *J* = 7.3 Hz, 3H), 1.16 (d, *J* = 4.7 Hz, 3H), 0.99 (t, *J* = 7.5 Hz, 3H). HRMS (ESI): *m*/*z* calcd C_27_H_33_N_4_O_8_ [M + H]^+^: 541.2298, found: 541.2314.

##### N-((5R,6S,9S,10S,11R,Z)-2-(Butan-2-ylidene)-10-hydroxy-5,11-dimethyl-3,7,12-trioxo-9-(pyridin-3-ylmethyl)-1,4-dioxa-8-azacyclododecan-6-yl)-3-hydroxyisonicotinamide (**6**)

Compound **6** was prepared through the coupling of 3-hydroxynicotinic acid (Sigma-Aldrich) with compound **2** according to the general procedure in 20% yield. ^1^H NMR (600 MHz, (CD_3_)_2_SO): δ 8.75 (d, *J* = 7.3 Hz, 1H), 8.62 (br, 1H), 8.52 (doublet br, *J* = 4.99 Hz, 1H), 8.38 (br, 1H), 8.16 (d, *J* = 5.04 Hz, 1H), 8.11 (doublet br, *J* = 7,80 Hz, 1H), 7.86–7.74 (br, 2H), 7.57 (dd, *J* = 7,5, 6.7 Hz, 1H), 4.75 (br, 1H), 4.22–4.15 (br, 1H), 3.74 (t, *J* = 2.1 Hz, 1H), 3.07 (dd, *J* = 13.6, 5.8 Hz, 1H), 2.96 (dd, *J* = 13.6, 8.7 Hz, 1H), 2.70–2.64 (br, 1H), 2.21–2.07 (br, 6H), 1.39 (d, *J* = 10,86 Hz, 3H), 1.15 (br, 3H), 0.99 (t, *J* = 7.6 Hz, 3H). HRMS (ESI): *m*/*z* calcd C_27_H_33_N_4_O_8_ [M + H]^+^:541.2298, found:541.2308.

##### N-((5R,6S,9S,10S,11R,Z)-2-(Butan-2-ylidene)-10-hydroxy-5,11-dimethyl-3,7,12-trioxo-9-(pyridin-3-ylmethyl)-1,4-dioxa-8-azacyclododecan-6-yl)-2-hydroxynicotinamide (**7**)

Compound **7** was prepared through the coupling of 2-hydroxynicotinic acid (Alfa Aesar) with amine **2** according to the general procedure, with an 18% yield. ^1^H NMR (600 MHz, (CD_3_)_2_SO): δ 12.28 (br, 1H), 10.05 (d, *J* = 7.4 Hz, 1H), 8.51 (br, 1H), 8.40 (d, *J* = 4.3 Hz, 1H), 8.34 (dd, *J* = 7.2, 2.3 Hz, 1H), 7.86 (d, *J* = 7.41 Hz, 1H), 7.68 (br, 2H), 7.42 – 7.32 (dd, *J* = 7.4, 4.8 Hz 1H), 6.47 (t, *J* = 6.75 Hz, 1H), 5.14 (br, 1H), 4.70 (br, 1H), 4.18 – 4.06 (br, 1H), 3.66 (br, 1H), 2.98 (dd, *J* = 13.6, 6.7 Hz, 1H), 2.87 (dd, *J* = 13.62, 7.84 Hz, 1H), 2.68–2.59 (br, 1H), 2.19 – 2.08 (br, 6H), 1.37 (d, *J* = 7.3 Hz, 3H), 1.17 (br, 3H), 0.99 (t, *J* = 7.56 Hz, 3H). HRMS (ESI): *m*/*z* calcd C_27_H_33_N_4_O_8_ [M + H]^+^: 541.2298, found: 541.2320.

##### N-((5R,6S,9S,10S,11R,Z)-2-(Butan-2-ylidene)-10-hydroxy-5,11-dimethyl-3,7,12-trioxo-9-(pyridin-3-ylmethyl)-1,4-dioxa-8-azacyclododecan-6-yl)-2,6-dihydroxybenzamide (**8**)

Compound **8** was prepared through the coupling of 2,6-dihydroxybenzoic acid (Sigma-Aldrich) with amine **2** according to the general procedure, with a 14% yield. ^1^H NMR (600 MHz, (CD_3_)_2_SO): δ 9.24 (br, 1H), 8.54 (s, 1H), 8.42 (d, *J* = 4.4 Hz, 1H), 7.89 (d, *J* = 7.8 Hz, 1H), 7.78 (d, *J* = 6.0 Hz, 1H), 7.42 – 7.34 (br, 1H), 7.19 (t, *J* = 8.2 Hz, 1H), 6.39 (s, 1H), 6.38 (s, 1H), 5.19 (br, 1H), 4.79 (br, 1H), 4.18–4.12 (br, 1H), 3.70 (t, *J* = 2.1 Hz, 1H), 3.00 (dd, *J* = 13.6, 6.3 Hz, 1H), 2.89 (dd, *J* = 13.6, 8.1 Hz, 1H), 2.65 (q, *J* = 7.5 Hz, 1H), 2.23–2.01 (br, 6H), 1.38 (d, *J* = 7.3 Hz, 3H), 1.21 – 1.10 (d, *J* = 4.1 Hz, 3H), 0.99 (t, *J* = 7.5 Hz, 3H). HRMS (ESI): *m*/*z* calcd C_28_H_34_N_3_O_9_ [M + H]^+^: 556.2295, found: 556.2294.

##### N-((5R,6S,9S,10S,11R,Z)-2-(Butan-2-ylidene)-10-hydroxy-5,11-dimethyl-3,7,12-trioxo-9-(pyridin-3-ylmethyl)-1,4-dioxa-8-azacyclododecan-6-yl)-2,3-dihydroxybenzamide (**9**)

Compound **9** was prepared through the coupling of 2,3-dihydroxybenzoic acid (Enamine) with amine **2** according to the general procedure, with a 30% yield. ^1^H NMR (600 MHz, (CD_3_)_2_SO): δ 8.56 (br, 1H), 8.44 (d, *J* = 4.7 Hz, 1H), 8.41 (d, *J* = 7.3 Hz, 1H), 7.94 (d, *J* = 7.7 Hz, 1H), 7.74 (br, 1H), 7.43 (dd, *J* = 7.1, 5.5 Hz, 1H), 7.34 (dd, *J* = 8.0, 1.2 Hz 1H), 6.97 (dd, *J* = 7.8, 1.5 Hz, 1H), 6.73 (t, *J* = 7.9 Hz, 1H), 5.17 (br, 1H), 4.74 (br, 1H), 4.18 – 4.11 (br, 1H), 3.69 (br, 1H), 3.02 (dd, *J* = 13.6, 6.3 Hz, 1H), 2.92 (dd, *J* = 13.6, 8.1 Hz, 1H), 2.69–2.62 (q, *J* = 6.9, 1H), 2.20 – 1.99 (br, 6H), 1.38 (d, *J* = 7.3 Hz, 3H), 1.19 (d, *J* = 3.9 Hz, 3H), 0.99 (t, *J* = 7.6 Hz, 3H). HRMS (ESI): *m*/*z* calcd C_28_H_34_N_3_O_9_ [M + H]^+^: 556.2295, found: 556.2277.

##### N-((5R,6S,9S,10S,11R,Z)-2-(Butan-2-ylidene)-10-hydroxy-5,11-dimethyl-3,7,12-trioxo-9-(pyridin-3-ylmethyl)-1,4-dioxa-8-azacyclododecan-6-yl)-3-chloro-2-hydroxybenzamide (**10**)

Compound **10** was prepared through the coupling of 3-chloro-2-hydroxybenzoic acid (Apollo Scientific) with amine **2** according to the general procedure, with a 26% yield. ^1^H NMR (600 MHz, (CD_3_)_2_SO): δ 8.56 (br, 1H), 8.52 – 8.40 (br, 2H), 7.95 (d, *J* = 7.7 Hz, 1H), 7.88 (dd, *J* = 8.0, 1.3 Hz, 1H), 7.79 (br, 1H), 7.59 (dd, *J* = 7.9, 1.5 Hz, 1H), 7.43 (br, 1H), 6.94 (t, *J* = 7.9 Hz, 1H), 5.15 (br, 1H), 4.74 (br, 1H), 4.20 – 4.14 (br, 1H), 3.71 (br, 1H), 3.02 (dd, *J* = 13.7, 6.0 Hz, 1H), 2.94 (dd, *J* = 13.7, 8.3 Hz, 1H), 2.71 – 2.63 (quadruplet br, J = 7.2 Hz, 1H), 2.24 – 2.03 (br, 6H), 1.39 (d, *J* = 7.3 Hz, 3H), 1.24 (d, *J* = 3,6 Hz, 3H), 0.99 (t, *J* = 7.6 Hz, 3H). HRMS (ESI): *m*/*z* calcd C_28_H_33_ClN_3_O_8_ [M + H]^+^: 574.1956, found 574.1977.

##### N-((5R,6S,9S,10S,11R,Z)-2-(Butan-2-ylidene)-10-hydroxy-5,11-dimethyl-3,7,12-trioxo-9-(pyridin-3-ylmethyl)-1,4-dioxa-8-azacyclododecan-6-yl)-3-fluoro-2-hydroxybenzamide (**11**)

Compound **11** was prepared through the coupling of 3-fluoro-2-hydroxybenzoic acid (Apollo Scientific) with amine **2** according to the general procedure, with an 8% yield. ^1^H NMR (600 MHz, (CD_3_)_2_SO): δ 8.57–8.53 (br, 2H), 8.42 (doublet br, J = 4.56 Hz, 1H), 7.90 (d, *J* = 7.8 Hz, 1H), 7.77 (br, 1H), 7.72 (doublet br, J= 8.04 Hz, 1H), 7.44 – 7.38 (br, 1H), 7.37 – 7.34 (ddd, J= 10.7, 8.1, 1.6 Hz 1H), 6.92 (td, *J* = 8.1, 4.9 Hz, 1H), 5.17 (br, 1H), 4.77 (br, 1H), 4.19–4.12 (br, 1H), 3.70 (br, 1H), 3.00 (dd, *J* = 13.6, 6.3 Hz, 1H), 2.91 (dd, *J* = 13.6, 8.1 Hz, 1H), 2.68 – 2.63 (q, J= 7.1 Hz, 1H), 2.23 – 2.05 (br, 6H), 1.38 (d, *J* = 7.3 Hz, 3H), 1.20 (d, 7.4 Hz, 3H), 0.99 (t, *J* = 7.6 Hz, 3H). HRMS (ESI): *m*/*z* calcd C_28_H_33_FN_3_O_8_ [M + H]^+^: 558.2252, found 558.2242.

##### N-((5R,6S,9S,10S,11R,Z)-2-(Butan-2-ylidene)-10-hydroxy-5,11-dimethyl-3,7,12-trioxo-9-(pyridin-3-ylmethyl)-1,4-dioxa-8-azacyclododecan-6-yl)-4-chloro-3-hydroxypicolinamide (**12**)

Compound **12** was prepared through the coupling of 4-chloro-3-hydroxypicolinic acid (Enamine) with amine **2** according to the general procedure, with a 40% yield. ^1^H NMR (600 MHz, (CD_3_)_2_SO): δ 8.55 (br, 1H), 8.40 (d, *J* = 3.8 Hz, 1H), 8.09 (d, *J* = 5.0 Hz, 1H), 7.96–7.84 (br, 2H), 7.79 (d, *J* = 5.0 Hz, 1H), 7.42 (br, 1H), 5.18 (br, 1H), 4.78 (br, 1H), 4.20–4.14 (br, 1H), 3.72 (t, *J* = 2.1 Hz, 1H), 3.01 (dd, *J* = 13.6, 6.1 Hz, 1H), 2.91 (dd, *J* = 13.6, 8.4 Hz, 1H), 2.70–2.63 (m, 1H), 2.29–1.99 (m, 6H), 1.39 (d, *J* = 7.2 Hz, 3H), 1.15 (d, *J* = 3,12 Hz, 3H), 0.99 (t, *J* = 7.6 Hz, 3H). HRMS (ESI): *m*/*z* calcd C_27_H_32_ClN_4_O_8_ [M + H]^+^: 575.1909, found: 575.1914.

##### N-((5R,6S,9S,10S,11R,Z)-2-(Butan-2-ylidene)-10-hydroxy-5,11-dimethyl-3,7,12-trioxo-9-(pyridin-3-ylmethyl)-1,4-dioxa-8-azacyclododecan-6-yl)-4-fluoro-3-hydroxypicolinamide (**13**)

Amine **2** (47.3 mg, 0.123 mmol, 1.00 equiv) was dissolved in dry DMF (1 mL). Meanwhile, a solution of 4-fluoro-3-HPA (19.5 mg, 0.124 mmol, 1.10 equiv), HATU (51.4 mg, 0.135 mmol, 1.20 equiv), and DIEA (0.059 mL, 0.338 mmol, 3.00 equiv) in dry DMF (2.5 mL) was prepared. This solution was stirred for 1 min at room temperature and then transferred to the solution of **2**. The reaction mixture was stirred overnight at room temperature, during which time it turned dark yellow/brown. The reaction mixture was diluted with EtOAc (ca. 20 mL), followed by the addition of sat. aq. NaHCO_3_ solution (ca. 10 mL). The phases were separated, and the aqueous phase was extracted with EtOAc (3 × 20 mL). The organic layers were combined, dried over MgSO_4_, filtered, and concentrated under reduced pressure. A silica cake (0.5 g) with the compound was prepared in MeOH. Purification by flash column chromatography with CH_2_Cl_2_/MeOH (95:5 to 9:1) and subsequent preparative HPLC (SymmetryPrep C18 5 μm 19 × 100 mm column, gradient: 5 to 25% MeCN-0.1% TFA in H_2_O-0.1% TFA in 19 min, flow: 25 mL/min, rt, t_R_ = 14.8 min) gave **13** as a slightly yellow solid (31.5 mg, 50%). [α]_D_^20^ = −57.99° (c = 1.00, MeOH); ^1^H NMR (500 MHz, CD_3_OD): δ (ppm) = 8.74 (s, 1H), 8.52 (d, *J* = 5.6 Hz, 1H), 8.38 (d, *J* = 7.9 Hz, 1H), 8.10–8.01 (m, 1H), 7.71 (s, 1H), 7.44 (dd, *J* = 10.3, 5.4 Hz, 1H), 5.34 (s, 1H), 4.68 (d, *J* = 6.3 Hz, 1H), 4.31 (s, 1H), 3.84 (s, 1H), 3.26–3.09 (m, 2H), 2.76 (d, *J* = 7.9 Hz, 1H), 2.26 (d, *J* = 9.3 Hz, 1H), 2.22 (s, 3H), 2.17–2.02 (m, 1H), 1.53 (d, *J* = 7.4 Hz, 3H), 1.31–1.20 (m, 3H), 1.15 (s, 1H), 1.04 (t, *J* = 7.6 Hz, 3H); ^13^C NMR (126 MHz, CD_3_OD): δ (ppm) = 177.17, 173.77, 170.18, 162.68, 162.40, 161.32, 148.76, 147.71, 144.59, 141.53, 140.30, 140.26, 132.92, 127.37, 119.14, 116.82, 116.48, 116.36, 76.40, 69.20, 56.91, 54.89, 42.10, 36.81, 27.94, 18.01, 17.58, 14.72, 11.85; IR (neat): [cm^–1^] = 2979, 2941, 1724, 1658, 1564, 1525, 1458, 1378, 1322, 1289, 1251, 1225, 1201, 1134, 1100, 1071, 996, 874, 865, 827, 799, 772, 720, 686.

HRMS (ESI): *m*/*z* calcd C_27_H_32_N_4_O_8_F [M + H]^+^: 559.2204, HRMS found: 559.2206

##### N-((5R,6S,9S,10S,11R,Z)-2-(Butan-2-ylidene)-10-hydroxy-5,11-dimethyl-3,7,12-trioxo-9-(pyridin-3-ylmethyl)-1,4-dioxa-8-azacyclododecan-6-yl)-3-hydroxypyrazine-2-carboxamide (**14**)

DIEA (30.5 μL, 176 μmol, 3.20 equiv) was added to a solution of 2-hydroxy-3-pyrazinecarboxylic acid (17.0 mg, 121 μmol, 2.2 equiv) and HATU (46.1 mg, 121 μmol, 2.2 equiv) in DMF (0.4 mL). The solution was stirred for 5 min and amine **2** (23.1 mg, 55.1 μmol, 1.00 equiv) in DMF (0.8 mL) was added at rt. The mixture was stirred for 18 h at rt. The mixture was diluted with EtOAc (2 mL) and sat. NaHCO_3_ (2 mL). The aqueous phase was extracted with EtOAc (3 × 5 mL), and the combined organic phases were dried over MgSO_4_, filtered, and concentrated *in vacuo*. The remaining orange oil was purified by flash column chromatography (CH_2_Cl_2_/MeOH 15%) to yield **14** (7.4 mg). The sample prepared for biological testing was purified by reversed-phase HPLC (Symmetry C18 5 μm 19 × 100 mm column, gradient: 15 → 40% MeCN/0.1% TFA in H_2_O/0.1% TFA in 14 min, flow: 25 mL/min, rt, t_R_ *=* 7.9 min) to a purity >98%. 4.50 mg (13%, as a TFA salt) were collected. ^1^H NMR (500 MHz, (CD_3_)_2_SO) δ 9.64 (s, 1H), 8.61 (s, 1H), 8.58–8.46 (m, 1H), 8.18 (s, 1H), 8.05 (s, 1H), 7.80 (s, 1H), 7.69 (s, 2H), 5.20 (s, 1H), 4.90 (d, *J* = 27.9 Hz, 1H), 4.63 (s, 1H), 4.13 (q, *J* = 8.2 Hz, 1H), 3.04 (dd, *J* = 13.3, 5.4 Hz, 1H), 2.91 (dd, *J* = 13.3, 9.1 Hz, 1H), 2.66 (s, 1H), 2.34–1.88 (m, 6H), 1.36 (d, *J* = 7.2 Hz, 3H), 1.21–1.04 (m, 3H), 0.95 (t, *J* = 7.6 Hz, 3H).

HRMS (ESI): *m*/*z* calcd C_26_H_32_N_5_O_8_ [M + H]^+^: 542.2245, found: 542.2249

##### N-((5R,6S,9S,10S,11R,Z)-2-(Butan-2-ylidene)-10-hydroxy-5,11-dimethyl-3,7,12-trioxo-9-(pyridin-3-ylmethyl)-1,4-dioxa-8-azacyclododecan-6-yl)-4-oxo-4,5-dihydro-1,2,5-thiadiazole-3-carboxamide (**15**)

Compound **15** was prepared through the coupling of 4-hydroxy-1,2,5-thiadiazole-3-carboxylic acid (Enamine) with amine **2** according to the general procedure, with a 12% yield. ^1^H NMR (600 MHz, (CD_3_)_2_SO): δ 8.54 (br, 1H), 8.44 (d, *J* = 4.6 Hz, 1H), 8.03 (d, *J* = 6.9 Hz, 1H), 7.94 (d, *J* = 7.7 Hz, 1H), 7.82 (br, 1H), 7.46 (dd, *J* = 6.9, 5.43 Hz, 1H), 5.15 (br, 1H), 4.74 (br, 1H), 4.17–4.12 (br, 1H), 3.71 (t, *J* = 2.0 Hz, 1H), 3.01 (dd, *J* = 13.5, 6.0 Hz, 1H), 2.91 (dd, *J* = 13.5, 8.4 Hz, 1H), 2.7 (br, 1H), 2.21–2.06 (br, 6H), 1.39 (d, *J* = 7.3 Hz, 3H), 1.15 (br, 3H), 0.99 (t, *J* = 7.6 Hz, 3H).

HRMS (ESI): *m*/*z* calcd C_24_H_30_N_5_O_8_S [M + H]^+^: 548.1815, HRMS found: 548.1849.

#### *In Vitro* Susceptibility of *Mtb* to Pyridomycin and Derivatives

The antibiotic activity of pyridomycin and derivatives was evaluated on the parental wild-type *Mtb* strain H37Rv, a pyridomycin-resistant H37Rv isolate with an M161L mutation in InhA (H37Rv:InhA­(M161L)),^19^ an H37Rv strain overexpressing *inhA* (H37Rv::pMVinhA), and a pMV261 vector control strain (H37Rv::pMV261).^11^ For the evaluation of antibiotic activity, susceptibility testing of *Mtb* strains was performed on bacteria grown in Middlebrook 7H9 media supplemented with 0.2% glycerol, 0.05% Tween 80, and 10% OADC (Gibco). Pyridomycin and derivatives were dissolved in DMSO. The minimal inhibitory concentration (MIC) of compounds on mycobacterial strains was evaluated using the resazurin microtiter assay as described previously,^11^ and expressed as the concentration of compound needed to prevent 95% of resazurin turnover (MIC_95_).

#### Plasma Stability of Pyridomycin Derivatives

To evaluate the plasma stability of pyridomycin and its derivatives, compounds (10 μM) were incubated in prewarmed mouse (CD-1) female plasma (BioIVT), in duplicate, at 37 °C; enalapril was used as a control compound for rapid plasma metabolism. Following 0, 15, 30, 60, 130, 240, and 360 min of incubation, 50 μL aliquots were transferred to tubes containing ice-cold acetonitrile and shaken vigorously to inactivate plasma proteins. Samples were then centrifuged (10 min, 13,000*g*, 4 °C), and supernatants were transferred to Matrix tubes for LC-MS/MS analysis. Briefly, samples were analyzed on a UPLC system Acquity I-Class (Waters) coupled to a triple quadrupole mass spectrometer, Xevo TQSμ (Waters), under multiple reaction monitoring (MRM) detection. The Waters Acquity BEH C18 column (50 × 2.1 mm, 1.7 μm, Waters) was placed at 40 °C, the flow rate was 600 μL/min, the injection volume was 1 μL, and the mobile phase was 5 mM ammonium formate pH 3.8 in H_2_O (A) and 5 mM ammonium formate pH 3.8 in acetonitrile (B). The gradient was initiated at 2% B, maintained for 10 s, then increased linearly to 98% B in 110 s, and maintained at 98% B for 30 s before returning to initial conditions. The MRM parameters (capillary voltage, cone voltage, and collision energy) were optimized for each compound. The half-life values (*t*_1/2_) for compounds were calculated from a nonlinear regression of the degradation time course data using the Xlfit software from IDBS Ltd.

#### Mouse Liver Microsome Stability of Pyridomycin and Derivatives

The *in vitro* metabolic stability of pyridomycin and its derivatives was determined using liver microsomes from female mice (CD-1, Corning). Briefly, in a final volume of 0.5 mL of 50 mM phosphate buffer (pH 7.4) with 5 mM MgCl_2_, liver microsomes (0.15 mg of protein) were mixed with a NADPH regenerating system (glucose-6-phosphate dehydrogenase (0.4 U/mL) with glucose-6-phosphate (5 mM)); propranolol was used as a control compound for high hepatic clearance. Following preincubation at 37 °C, the incubation mixture was spiked with pyridomycin or derivatives (final concentration of 1 μg/mL) and incubated at 37 °C. 50 μL aliquots were taken at 5, 10, 20, 30, and 40 min of incubation and quenched in 4 volumes of ice-cold acetonitrile. After centrifugation (13,000*g*, 10 min, 4 °C), the supernatants were transferred into Matrix tubes for LC-MS/MS analysis, as described above. Control incubations (*t*_0_ and *t*_final_) were performed with microsomes denatured by acetonitrile.

The quantification of compounds was performed by converting the averages of the areas of the analyte into the percentages of product consumed. The half-life (*t*_1/2_) was calculated from nonlinear regression (exponential decay) applied in the XlfitTM software (IDBS Ltd.). *In vitro* intrinsic clearance (Cl_int_ expressed in μL/min/mg) was calculated according to the equation Cl_int_ = |k|/[microsomes] where k is the first-order degradation constant (i.e., the slope of the logarithm of compound concentration as a function of incubation time) and [microsomes] is the concentration in microsomes expressed in mg/μL.

#### Murine Pharmacokinetic Analysis of Pyridomycin and Derivatives

Animals were maintained in compliance with European standards for the care and use of laboratory animals, and experimental protocols were approved by the local Animal Ethical Committee (agreement no. APAFIS# 01134.03).

Compound pharmacokinetics was performed similarly to that described previously, with minor changes.^23^ Briefly, pyridomycin and derivatives (as TFA salts) were solubilized in PBS (1% DMSO) at 1 mg/mL. Six-week-old female CD-1 mice were purchased from Charles River (Saint Germain Nuelles, France). All animals were maintained in standard animal cages under conventional laboratory conditions (12 h/12 h light/dark cycle, 22 °C) with *ad libitum* access to food and water. Compounds of interest were then dosed at 10 mg/kg by intraperitoneal (i.p.) administration. At 15, 30, 45, 60, 120, and 240 min post dosing, the mice were anesthetized with isoflurane and blood was collected from the retro-orbital sinus using sampling heparinized tubes (4 °C). The blood samples were centrifuged (2500*g*, 20 min, 4 °C) for plasma separation and stored at −80 °C. The compounds were extracted from the plasma with an ice-cold acetonitrile solution containing an internal standard (100 nM propranolol) in a ratio of 1:10. After centrifugation (13,000*g*, 10 min, 4 °C), the supernatants were transferred to Matrix tubes for LC-MS/MS analysis (MRM detection), as described above.

Standard curves were generated using plasma from a naive female mouse spiked with the appropriate compound solutions, resulting in 10 different concentrations of each compound tested. These standard curves were then extracted as plasma samples (in a ratio of 1:10), leading to a concentration of 0.3 to 10,000 nM. The samples were analyzed by LC-MS/MS. Peak areas were quantified using TargetLynx software (Waters).

#### *In Vivo* Efficacy of Pyridomycin and Derivative 13 against *Mtb*-Infected Mice

The sanitary status and well-being of mice were monitored, and individual weight changes were recorded regularly, following approved ethics protocols. Mouse infection study was performed in agreement with European regulations and guidelines (EC Directive 2010/63/UE) in the framework of experimental procedures approved by the Ethics Commission of Sciensano for animal experiments No. 20200107-01.

The *in vivo* efficacy of pyridomycin (**1**) TFA salt and compound (**13)** TFA salt (with and without ABT) was evaluated in a fast-acute mouse model of *Mtb* infection.^24^ Female balbC mice (Charles River) were anaesthetized with ketamine-xylazine i.p. and instilled intratracheally with around 1 × 10^5^ colony-forming units (CFU) in 100 μL of luminescent *M. tuberculosis* H37Rv-luxAB^25^ PBST (prepared from frozen stock). Mice were randomized into the required number of groups (5 mice per group). The infective bacterial load in the lungs was determined 1 and 7 days post infection by measuring the relative luminescence units of homogenized lungs. Treatment was initiated 7 days post infection for 6 days, in the following groups: (i) no treatment control, (ii) once daily 25 mg/kg po isoniazid, and (iii) twice daily 25 mg/kg ip pyridomycin (**1**).TFA, (iv) twice daily 25 mg/kg i.p. comp. **13**.TFA, (v) twice daily 50 mg/kg p.o ABT followed by 25 mg/kg i.p. pyridomycin (**1**).TFA 1 h later and (vi) twice daily 50 mg/kg p.o ABT followed by 25 mg/kg i.p. comp. **13**.TFA 1 h later. Pyridomycin (**1**).TFA and Comp. **13**.TFA were dissolved in acidified phosphate-buffered saline (PBS) with 1% DMSO (2.5 mg/mL). ABT was formulated at 5 mg/mL in 0.5% carboxymethyl cellulose in PBS. Isoniazid was formulated in PBS (2.5 mg/mL). Fourteen days post infection, the treatment groups were sacrificed and bacterial load determined by measuring the luminescence of lung extracts (RLU) as well as lung bacterial load by CFUs.

#### Structural Biology of InhA in Complex with Pyridomycin and Derivatives

Production and purification of InhA were performed as recently published^26^ except that the last gel filtration step after cleavage of the His_6_ tag was performed on a preparative gel filtration HiLoad 16/60 Superdex 200 (Cytiva) preequilibrated with buffer 30 mM PIPES, 150 mM NaCl at pH 6.1 instead of 6.8.

Structures of complexes with pyridomycin derivatives were obtained after cocrystallization or soaking using the *apo*-form of InhA. For pyridomycin (**1**), and derivatives **4**, **5**, **9**, **11**, **12**, **14**, and **15**, untagged InhA (in 30 mM PIPES, 150 mM NaCl, pH 6.1) was incubated at 8 mg/mL with ligands at 0.9–1.8 mM final concentrations in 10–20% DMSO. After 1 h incubation at 4 °C, crystallization assays were performed using the vapor diffusion method at 20 °C by mixing, with the help of a Mosquito crystallization robot (SPT Labtech, Melbourne, UK), 200 nL of InhA previously incubated with ligands with 200 nL of reservoir solutions. In the case of pyridomycin (**1**) and derivatives **11**, **14**, and **15**, the reservoir solutions contained 30% (v/v) PEG 300, 0.1 M MES pH 6.5. For compounds **4**, **5**, **9**, and **12**, the reservoir solutions contained 30% PEG 550 MME, 0.1 M NaCl, and 0.1 M Bicine pH 9. With respect to compound **13**, crystals of *apo*-InhA were obtained in 30% PEG 400, 0.1 M MgCl_2_, and 0.1 M MES pH 6.7 and soaked in a solution containing 0.9 mM of ligand and 10% DMSO for 150 min. All crystals were cryoprotected with paraffin oil before being flash frozen and stored in liquid nitrogen. Diffraction data were collected at the ALBA beamline XALOC,^27^ ESRF beamline ID30A3,^28^ and SOLEIL beamline PX1 ^29^ and processed using autoPROC^29^ and XDS.^30^ Structure refinement was initiated with Pipedream,^31^ and pursued with alternated cycles of manual corrections with Coot^32^ and refinement with Buster.^33^ Data processing and model refinement statistics are listed in Table S2. Dictionaries for pyridomycin and its derivatives were generated with the grade Server 2.^34^

## Supplementary Material





## Notes

Chemical synthesis of synthetic 2-cyclohexyl-dihydropyridomycin variants **S1–S6**, methods and results for the evaluation of mycolic acid production, supplemental structures of InhA bound to pyridomycin derivatives, crystallographic data and refinement table, and NMR, HRMS, and HPLC spectra of compounds **2–15** and **S3–S6** (PDF)
Molecular formula strings (CSV)

## Abbreviations Used

ABT: 1-aminobenzotriazole
MRM: multiple reaction monitoring
SIC: selected ion chromatography
*Mt*b: *Mycobacterium tuberculosis*
MLM: mouse liver microsomes
HLM: human liver microsomes
RLU: relative luminescence units
CFU: colony-forming units
