# Supplementary material for: Optimizing the Antibiotic Potency and Metabolic Stability of Pyridomycin Using a Semisynthetic Approach
Source: J Med Chem. 2026 Jan 27;69(3):2496–508. doi: 10.1021/acs.jmedchem.5c02409 (PMC12910649; doi:10.1021/acs.jmedchem.5c02409)
Supplement: Supplementary file 1 [file jm5c02409_si_001.pdf]

Supporting Information:

Manuscript title: Optimizing the antibiotic potency and metabolic stability of pyridomycin using a semi-synthetic approach

AUTHOR LIST: *Katherine Valderrama[1#], Oliver Horlacher[2#], Gabriel Publicola[3#], Patrick Eisenring[2], Maryline Kienle[2], Samira Boarbi[4], Mehdi Kiass[4], Jana Korduláková[5], Jonathan Chatagnon[1], Catherine Piveteau[6], Florence Leroux[6], Karin Savková[5], Monika Záhorszká[5], Francois-Xavier Cantrelle[7, 8], Christian Lherbet[9], Lionel Mourey[3], Katarína Mikušová[5], Vanessa Mathys[4], Reiner Aichholz[10], Laurent Maveyraud[3*], Karl-Heinz Altmann[2*], Ruben C. Hartkoorn[1*]*

[#] Joint first authors: Katherine Valderrama, Oliver Horlacher & Gabriel Publicola. These authors contributed equally.

[*] Joint last and corresponding authors: Laurent Maveyraud, Karl-Heinz Altmann & Ruben C. Hartkoorn. These authors jointly supervised this work.

AUTHOR AFFILIATIONS

[1] Univ. Lille, CNRS, Inserm, CHU Lille, Institut Pasteur Lille, U1019 - UMR 9017 - CIIL - Center for Infection and Immunity of Lille, F-59000 Lille, France.

[2] Department of Chemistry and Applied Biosciences, Institute of Pharmaceutical Sciences, ETH Zürich, 8093 Zurich, Switzerland

[3] Univ. Toulouse, CNRS, IPBS, 31077 Toulouse, France.

[4] Unit "Tuberculosis & Mycobacteria", Human Bacterial Diseases Service, Infectious Diseases in Humans, Sciensano, 1050 Brussels, Belgium.

[5] Faculty of Natural Sciences, Department of Biochemistry, Comenius University in Bratislava, Ilkovičova 6, Mlynská dolina, 842 15 Bratislava, Slovakia.

[6] Univ. Lille, Inserm, Institut Pasteur de Lille, U1177 - Drugs and Molecules for Living Systems, F-59000, Lille, France.

[7] CNRS, EMR9002 BSI Integrative Structural Biology, 59000 Lille, France.

[8] Univ. Lille, Inserm, CHU Lille, Institut Pasteur de Lille, U1167 - RID-AGE - Risk Factors and Molecular Determinants of Aging-Related Diseases, F-59000 Lille, France.

[9] Synthèse et Physico-Chimie de Molécules d'Intérêt Biologique (LSPCMIB), UMR 5068, CNRS, Université Toulouse (UT), 31062 Toulouse, France

[10] PK Sciences, Novartis Institutes for BioMedical Research, 4002 Basel, Switzerland.

CORRESPONDING AUTHOR EMAIL:
Laurent Maveyraud: Laurent.maveyraud@ipbs.fr,
Karl-Heinz Altmann: karl-heinz.altmann@pharma.ethz.ch,
Ruben C. Hartkoorn: ruben.hartkoorn@inserm.fr.

Table of Contents

1. Supplemental Methods & Results S3
1.1 Chemical synthesis of synthetic 2-cyclohexyl-dihydropyridomycin variants S1-S6 .... S3
1.2 Antibiotic activity of synthetic 2-cyclohexyl-dihydropyridomycin variants S1 & S3-S6 .... S7
Table S1 .... S7
1.3 Evaluation of Mycolic acid production in *Mtb* S8
Figure S1: S8
1.4 Supplemental structures of InhA bound to pyridomycin derivatives S9
Figure S2 (A-C) S9
Figure S2 (D-F) S10
Figure S2 (G-I) S11
1.4 Crystallographic data collection and refinement statistics S12
Table S2: S12
2. NMR, HRMS and HPLC Spectra of compounds 2-15 and S3-S6 .... S13
Compound 2 .... S13
Compound 3 .... S14
Compound 3 .... S15
Compound 4 .... S16
Compound 5 .... S18
Compound 6 .... S20
Compound 7 .... S22
Compound 8 .... S24
Compound 9 .... S26
Compound 10 .... S28
Compound 11 .... S30
Compound 12 .... S32
Compound 13 .... S34
Compound 14 .... S36
Compound 15 .... S38
Compound S3 .... S40
Compound S4 .... S40
Compound S5 .... S41
Compound S6 .... S41
3. References S42

1. Supplemental Methods & Results

1.1 Chemical synthesis of synthetic 2-cyclohexyl-dihydropyridomycin variants S1-S6

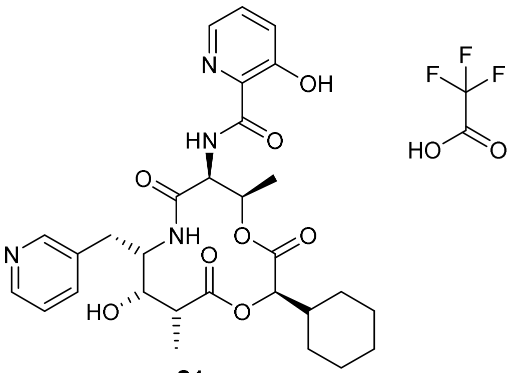

S1

N-((2R,5R,6S,9S,10S,11R)-2-Cyclohexyl-10-hydroxy-5,11-dimethyl-3,7,12-trioxo-9-(pyridin-3-ylmethyl)-1,4-dioxa-8-azacyclododecan-6-yl)-3-hydroxypicolinamide **(S1):**

The synthesis of **S1** and **S2** is described in ref. [1]

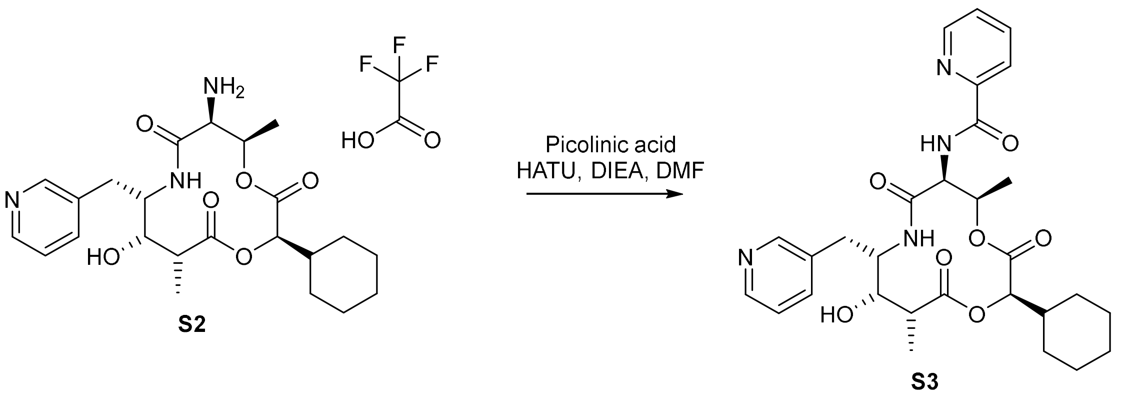

N-((2R,5R,6S,9S,10S,11R)-2-Cyclohexyl-10-hydroxy-5,11-dimethyl-3,7,12-trioxo-9-(pyridin-3-ylmethyl)-1,4-dioxa-8-azacyclododecan-6-yl)picolinamide **(S3):**

Synthesis of S3. DIEA (11.6 µL, 67.0 µmol, 3 eq.) was added to a solution of picolinic acid (3.02 mg, 24.6 µmol, 1.1 eq.), HATU (9.34 mg, 24.6 µmol, 1.1 eq.) and amine **S2** (as the trifluoroacetate, 12.5 mg, 22.3 µmol, 1.00 eq.) in MeCN (0.8 mL) at rt. (For the synthesis of **S2**, see ref. [1]). The mixture was stirred for 18 h at rt. The mixture was diluted with CH_2Cl_2 (2 mL) and sat. aq. $NaHCO_3$ (2 mL). The aq. phase was extracted with CH_2Cl_2 (3 x 3 mL) and the combined organic phases were dried over $MgSO_4$, filtered and concentrated *in vacuo*. The remaining orange oil was purified by FC (CH_2Cl_2/MeOH 5%) to yield **S3** (6.7 mg) as a colorless film. The samples prepared for biological testing were purified by reverse phase HPLC (Symmetry® C18 5 µm, 19x100 mm column,

gradient: 50% → 90% MeCN in H_2O in 12 min, flow: 25 mL/min, rt, t_R = 3.5 min) to a purity >98%. 3.57 mg (29%) were collected.

^{1}H NMR (500 MHz, $(CD_3)_2SO$): δ(ppm) 8.67 (ddd, *J* = 4.8, 1.5, 1.1 Hz, 1H), 8.37 (d, *J* = 1.9 Hz, 1H), 8.22 (d, *J* = 8.2 Hz, 1H), 8.20 (dd, *J* = 4.8, 1.6 Hz, 1H), 8.10 – 8.02 (m, 3H), 7.67 (ddd, *J* = 7.3, 4.8, 1.6 Hz, 1H), 7.53 (dt, *J* = 7.8, 1.9 Hz, 1H), 7.04 (dd, *J* = 7.8, 4.8 Hz, 1H), 5.28 (p, *J* = 6.4 Hz, 1H), 4.75 – 4.65 (m, 3H), 4.11 – 4.02 (m, 1H), 3.60 (d, *J* = 6.6 Hz, 1H), 2.86 (dd, *J* = 13.5,6.1 Hz, 1H), 2.75 (dd, *J* = 13.4, 8.6 Hz, 1H), 2.59 (qd, *J* = 7.0, 0.7 Hz, 1H), 1.86 – 1.76 (m,1H), 1.68 (dd, *J* = 12.4, 2.3 Hz, 2H), 1.60 (dd, *J* = 10.9, 0.7 Hz, 3H), 1.27 (d, *J* = 7.3 Hz, 3H), 1.24 – 1.01 (m, 8H).

HRMS (ESI): m/z calc. for $C_{29}H_{37}N_4O_7$ $[M+H]^+$: 553.2657, found 553.2654.

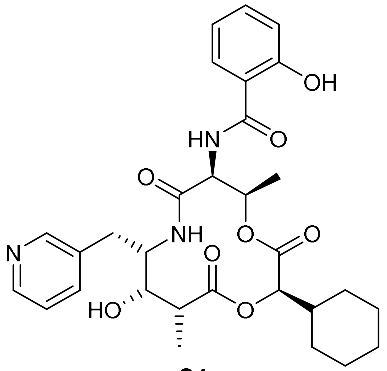

S4

N-((2R,5R,6S,9S,10S,11R)-2-Cyclohexyl-10-hydroxy-5,11-dimethyl-3,7,12-trioxo-9-(pyridin-3-ylmethyl)-1,4-dioxa-8-azacyclododecan-6-yl)-2-hydroxybenzamide **(S4):**

Synthesis of S4. DIEA (9.90 µL, 57.0 µmol, 3 eq.) was added to a solution of salicylic acid (2.89 mg, 20.9 µmol, 1.1 eq.), HATU (7.94 mg, 20.9 µmol, 1.1 eq.) and **S2** (8.50 mg, 19.0 µmol, 1 eq.) in MeCN (0.8 mL) at rt. The mixture was stirred for 18 h at rt. The mixture was diluted with EtOAc (2 mL) and sat. aq. $NaHCO_3$ (2 mL). The aq. phase was extracted with EtOAc (3 x 3 mL) and the combined organic phases were dried over $MgSO_4$, filtered and concentrated *in vacuo*. The orange oil was purified by FC (CH_2Cl_2/MeOH 5%) to yield **S4** (9.5 mg). The samples prepared for biological testing were purified by reverse phase HPLC (Symmetry® C18 5 µm 19x100 mm column, gradient: 40% → 65% MeCN in H_2O in 14 min, flow: 25 mL/min, rt, t_R = 8.2 min) to a purity >98%. 2.30 mg (21%) were collected.

^{1}H NMR (500 MHz, $(CD_3)_2SO$): δ(ppm) 8.93 (br. s, 1H), 8.38 (s, 1H), 8.27 (d, *J* = 4.0 Hz, 1H), 7.94 (d, *J* = 9.3 Hz, 1H), 7.90 (dd, *J* = 7.9, 1.7 Hz, 1H), 7.55 (d, *J* = 7.8 Hz, 1H), 7.39 – 7.30 (m, 1H), 7.09 (dd, *J* = 7.6, 4.8 Hz, 1H), 6.94 (d, *J* = 8.1 Hz, 1H), 6.88 (t, *J* = 7.5 Hz, 1H), 5.29 (p, *J* = 6.5 Hz, 1H), 4.74 – 4.63 (m, 3H), 4.03 (dd, *J* = 14.9, 7.7 Hz, 1H), 3.55 (d, *J* = 7.1 Hz, 1H), 2.86 (dd, *J* = 13.4, 6.7 Hz, 1H), 2.74 (dd, *J* = 13.4, 7.9 Hz, 1H), 2.56 (dd, *J* = 15.7, 8.4 Hz, 1H), 1.87 – 1.74 (m, 1H), 1.72 – 1.64 (m, 2H), 1.64 – 1.52 (m, 3H), 1.24 (d, *J* = 7.3 Hz, 3H), 1.22 (d, *J* = 6.5 Hz, 3H), 1.20 – 1.00 (m, 5H).

HRMS (ESI): *m/z* calc. for $C_{30}H_{38}N_3O_8$ $[M+H]^+$: 568.2653, found 568.2649.

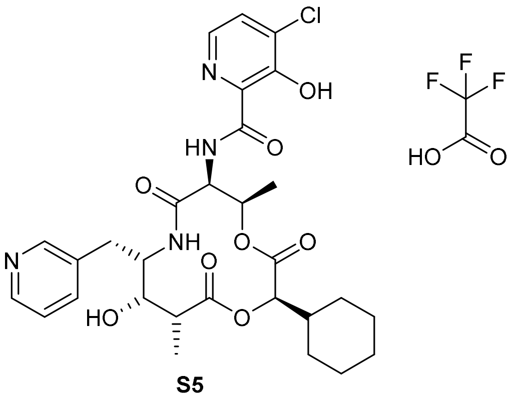

S5

6-Chloro-N-((2R,5R,6S,9S,10S,11R)-2-cyclohexyl-10-hydroxy-5,11-dimethyl-3,7,12-trioxo-9-(pyridin-3-ylmethyl)-1,4-dioxa-8-azacyclododecan-6-yl)-3-hydroxypicolinamide **(S5):**

Synthesis of S5. DIEA (18.6 µL, 107 µmol, 3 eq.) was added to a solution of 4-chloro-3-hydroxypicolinic acid (9.25 mg, 53.6 µmol, 1.50 eq.) and HATU (20.4 mg, 53.6 µmol, 1.50 eq.) in DMF (0.4 mL). The solution was stirred for 5 min and **S2** (16.0 mg, 35.8 µmol, 1.00 eq.) in DMF (0.8 mL) was added at rt. The mixture was stirred for 18 h at rt. The mixture was diluted with EtOAc (2 mL) and sat. aq. $NaHCO_3$ (2 mL). The aq. phase was extracted with EtOAc (3 x 3 mL) and the combined organic phases were dried over $MgSO_4$, filtered and concentrated *in vacuo*. The orange oil was purified by FC (CH_2Cl_2/MeOH 2.5% → 7.5%) to yield **S5** (10.2 mg). The samples prepared for biological testing were purified by reverse phase HPLC (Symmetry® C18 5 µm, 19x100 mm column, gradient: 30% → 80% MeCN-0.1% TFA in H_2O-0.1% TFA in 14 min, flow: 25 mL/min, rt, t_R = 5.9 min) to a purity >98%. 5.90 mg (23%, as a TFA salt) were collected.

^{1}H NMR (500 MHz, $(CD_3)_2SO$): δ (ppm) 8.63 (s, 1H), 8.52 (d, *J* = 5.0 Hz, 1H), 8.21 – 8.11 (m, 2H), 8.07 (s, 1H), 7.83 (d, *J* = 5.0 Hz, 1H), 7.64 – 7.55 (m, 1H), 5.27 (p, *J* = 6.3 Hz, 1H), 4.69 (d, *J* = 5.5 Hz, 1H), 4.67 – 4.62 (m, 1H), 4.21 – 4.11 (m, 1H), 3.69 (s, 1H), 3.04 (dd, *J* = 13.4, 5.0 Hz, 1H), 2.92 (dd, *J* = 13.2, 9.8 Hz, 1H), 2.60 (q, *J* = 7.1 Hz, 1H), 1.84 (s, 1H), 1.72 (s, 2H), 1.63 (s, 3H), 1.30 (d, *J* = 7.3 Hz, 3H), 1.17 (d, *J* = 6.6 Hz, 3H), 1.27 – 1.01 (m, 4H).

HRMS (ESI): *m/z* calc. for $C_{29}H_{36}ClN_4O_8$ [M+H]+: 603.2216, found 603.2222.

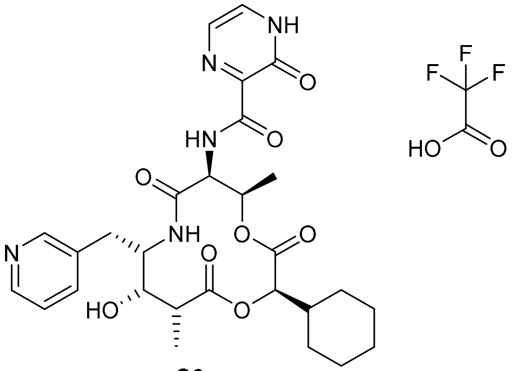

S6

N-((2R,5R,6S,9S,10S,11R)-2-Cyclohexyl-10-hydroxy-5,11-dimethyl-3,7,12-trioxo-9-(pyridin-3-ylmethyl)-1,4-dioxa-8-azacyclododecan-6-yl)-3-oxo-3,4-dihydropyrazine-2-carboxamide **(S6):**

Synthesis of S6. DIEA (28.5 µL, 165 µmol, 3.2 eq.) was added to a solution of 3-oxo-3,4-dihydropyrazine-2-carboxylic acid (15.8 mg, 113 µmol, 2.2 eq.) and HATU (43.0 mg, 113 µmol, 2.2 eq.) in DMF (0.4 mL). The solution was stirred for 5 min and **S2** (23.0 mg, 51.4 µmol, 1.00 eq.) in DMF (0.8 mL) was added at rt. The mixture was stirred for 18 h at rt. The mixture was diluted with EtOAc (2 mL) and sat. aq. $NaHCO_3$ (2 mL). The aq. phase was extracted with EtOAc (3 x 3 mL) and the combined organic phases were dried over $MgSO_4$, filtered and concentrated *in vacuo*. The orange oil was purified by FC (CH_2Cl_2/MeOH 10% → 20%) to yield **S6** (4.9 mg). The samples prepared for biological testing were purified by reverse phase HPLC (Symmetry® C18 5 µm, 19x100 mm column, gradient: 20% → 65% MeCN-0.1% TFA in H_2O-0.1% TFA in 14 min, flow: 25 mL/min, rt, t_R = 6.9 min) to a purity >98%. 3.50 mg (10%, as a TFA salt) were collected.

^{1}H NMR (500 MHz, $(CD_3)_2SO$): δ (ppm) 9.67 (s, 1H), 8.57 (s, 1H), 8.51 (d, *J* = 4.4 Hz, 1H), 8.11 (d, *J* = 7.7 Hz, 1H), 8.02 (d, *J* = 9.4 Hz, 1H), 7.84 – 7.76 (m, 1H), 7.74 – 7.66 (m, 1H), 7.65 – 7.57 (m, 1H), 5.25 (p, *J* = 6.1 Hz, 1H), 4.69 (d, *J* = 5.0 Hz, 1H), 4.65 – 4.58 (m, 1H), 4.14 – 4.05 (m, 1H), 3.66 (s, 1H), 3.02 (dd, *J* = 13.4, 5.0 Hz, 1H), 2.91 (dd, *J* = 12.8, 9.4 Hz, 1H), 2.58 (q, *J* = 6.9 Hz, 1H), 1.85 – 1.75 (m, 1H), 1.73 – 1.65 (m, 2H), 1.64 – 1.55 (m, 3H), 1.29 (d, *J* = 7.2 Hz, 3H), 1.18 (d, *J* = 6.5 Hz, 3H), 1.25 – 1.01 (m, 5H).

HRMS (ESI): *m/z* calc. for $C_{28}H_{36}N_5O_8$ $[M+H]^+$: 570.2558, found 570.2556.

1.2 Antibiotic activity of synthetic 2-cyclohexyl-dihydropyridomycin variants S1 & S3-S6

Table S1: Summary of the anti-mycobacterial activity of 2-cyclohexyl-dihydropyridomycin analogs **S1** and **S3-S6** on wild-type H37Rv, H37Rv vector control pMV261 and H37Rv overproducing InhA (H37Rv::pMV*inhA*). Antibiotic activity is presented as the concentration of compound (µg/mL) needed to prevent at least 95% of resazurin turnover (MIC_{95}) in the resazurin reduction microplate assay. Data are the average of two biological replicates.

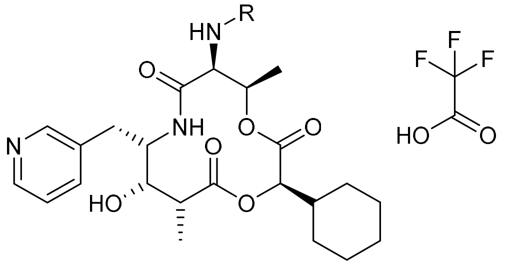

		Minimal inhibitory concentration (MIC_{95}) (µg/mL)		
Compound	R	H37Rv (MIC_{95}) µg/mL	H37Rv::pMV261	H37Rv::pMV*inhA* (fold vs pMV261)
**S1**	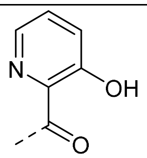	0.8	0.4	0.4 (1)
**S3**	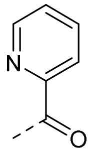	>100	ND	ND
**S4**	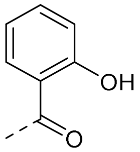	25	12.5	>100 (>4)
**S5**	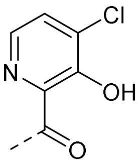	3.1	3.1	6.25 (2)
**S6**	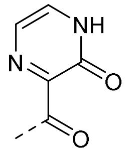	1.6	1.6	1.6 (1)

1.3 Evaluation of Mycolic acid production in *Mtb*

Mtb H37Rv was grown in Middlebrook 7H9 medium supplemented with 10% albumin-dextrose-catalase and 0.05% Tween 80 until OD_{600} = 0.48 at 37°C with shaking. Aliquots of 100 µL were transferred into Eppendorf tubes containing 2 µL of DMSO or 2 µL of pyridomycin derivatives in DMSO to achieve the final concentrations of 4, 8 or 20 µg/mL. After 26 h (37 °C, static incubation), 0.1 µCi of [^{14}C]-acetate (ARC; specific activity 10^6 mCi/mmol) was added to the cultures with a further incubation of 22 h (37 °C, static incubation). For each experiment two sets of the radiolabeled aliquots were prepared. One set was used for lipid extractions and the second set was used for preparation of the methyl esters of fatty acids and mycolic acid, as described previously[2].

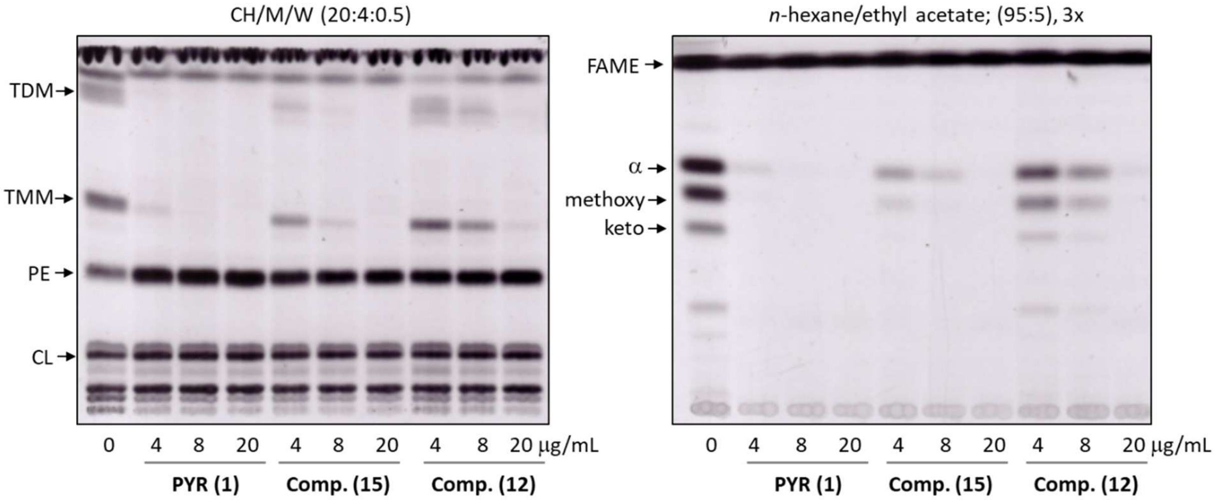

Figure S1: Evaluation of the effects of PYR (1), compound 15 and 12 on lipids and mycolic acids from *Mtb* H37Rv by metabolic labeling. *Left panel*: Lipids extracted from [^{14}C]-acetate labeled *Mtb* H37Rv grown in the absence and in the presence of the drugs were separated by TLC in $CHCl_3/CH_3OH/H_2O$ (20:4:0,5). *Right panel*: Methyl esters of fatty and mycolic acids isolated from [^{14}C]-acetate labeled *Mtb* H37Rv grown in the absence and in the presence of the drugs were separated by TLC in *n*-hexane/ethyl acetate; (95:5), developed 3 times. The radioactive bands were visualized by autoradiography. TMM, trehalose monomycolates; TDM, trehalose dimycolates; PE, phosphatidylethanolamine; CL, cardiolipin; FAME, methyl esters of fatty acids; α-, methoxy-, keto-, methyl esters of different forms of mycolic acids.

1.4 Supplemental structures of InhA bound to pyridomycin derivatives

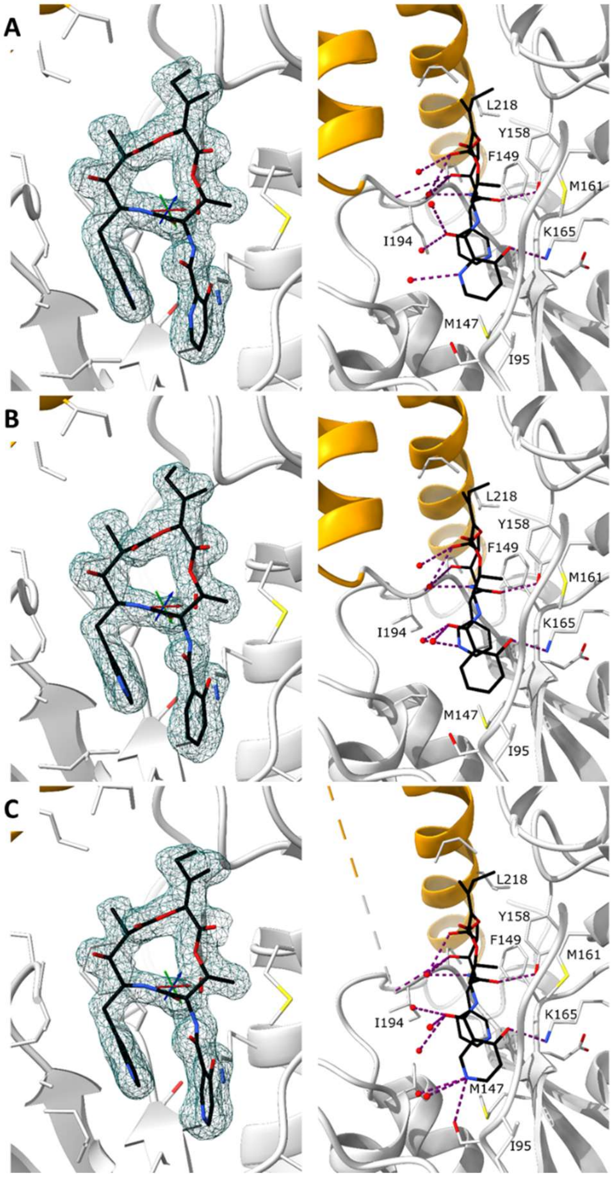

chain	H6	H7	lig.	occ.	F149	Y158
A	o	c	1	1.00	out	in
B	o	c	1	1.00	out	in
C	d	c	1	0.87	out	in
D	d	c	1	0.92	out	in
E	d	c	1	0.91	out	in
F	d	d	1	0.89	out	in

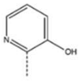

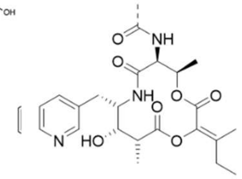

chain	H6	H7	lig.	occ.	F149	Y158
A	d/o	c	1	0.77	out	in
B	o	c	1	0.86	out	in
C	o	c	0	n/a	out	both
D	o	c	1	0.78	out	in
E	o	c	1	0.75	out	in
F	d	d/c	1	0.76	out	in

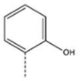

chain	H6	H7	lig.	occ.	F149	Y158
A	d	c	1	1.00	out	in
B	d	c	1	0.94	out	in
C	d/o	c	0	n/a	out	in
D	d	c	1	0.84	out	in
E	d	c	1	0.87	out	in
F	d	d/c	1	0.73	out	in

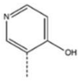

Figure S2: View of the interaction between the InhA active site and A) pyridomycin (**1**) (PDB: 9RJG), B) derivative **4** (PDB: 9RJH), and C) derivative **5** (PDB: 9RJI). For each compound, the left column shows the final 2*m*Fo-*D*Fc electron density map as a mesh, contoured at 1 s and carved around the bound compound (shown in black sticks). InhA protein is represented as a grey ribbon, with side chains of residues within 5 Å of bound compound displayed as sticks. The substrate binding loop is represented in orange. The middle column uses the same color code, and shows hydrogen bonds between bound compound and InhA or water molecules (represented as red spheres) as purple dashed lines. The orientation is rotated about 90° around the vertical axis. The right column summarizes in a table the structural features of each chain in the asymmetric unit of the crystal. Conformations of helices 6 (H6, residues 196-208) and 7 (H7, residues 209-226) are indicated as open (o), closed (c), disordered (d) (Rizet et al., 2025, ChemMedChem). Ligand binding in the specified chain is indicated with 1 (present) and 0 (absent) and the corresponding refined occupancy is indicated (n/a: not applicable). The conformation of Phe149 and Tyr148 is indicated as in or out (refs [3,4]). The chain highlighted in bold is shown in the figure.

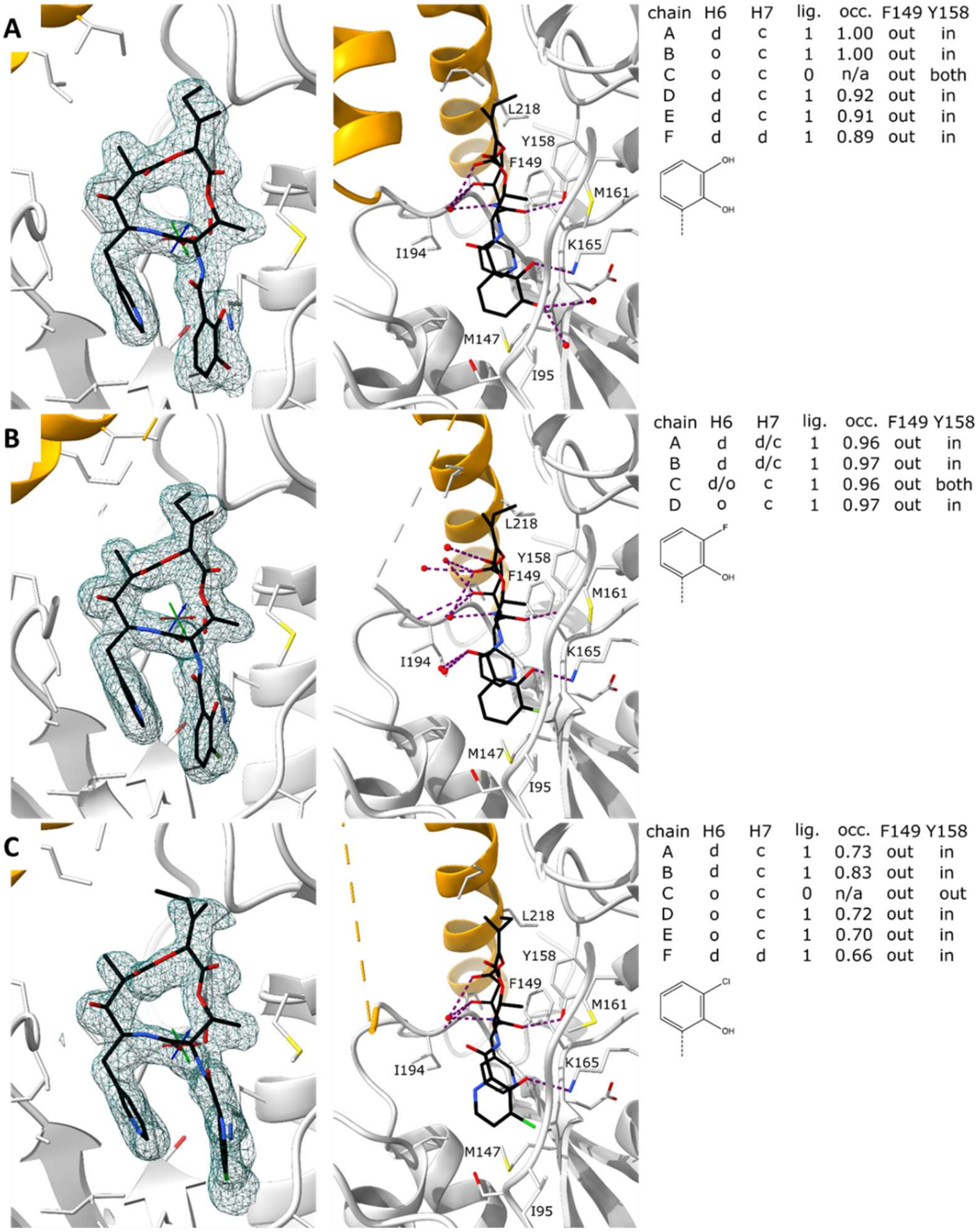

chain	H6	H7	lig.	occ.	F149	Y158
A	d	c	1	1.00	out	in
B	o	c	1	1.00	out	in
C	o	c	0	n/a	out	both
D	d	c	1	0.92	out	in
E	d	c	1	0.91	out	in
F	d	d	1	0.89	out	in

chain	H6	H7	lig.	occ.	F149	Y158
A	d	d/c	1	0.96	out	in
B	d	d/c	1	0.97	out	in
C	d/o	c	1	0.96	out	both
D	o	c	1	0.97	out	in

chain	H6	H7	lig.	occ.	F149	Y158
A	d	c	1	0.73	out	in
B	d	c	1	0.83	out	in
C	o	c	0	n/a	out	out
D	o	c	1	0.72	out	in
E	o	c	1	0.70	out	in
F	d	d	1	0.66	out	in

Figure S3: View of the interaction between the InhA active site and A) derivative **9** (PDB: 9RJJ), B) derivative **11** (PDB: 9RJK), and C) derivative **12** (PDB: 9RJL). For each compound, the left column shows the final 2*m*Fo-*D*Fc electron density map as a mesh, contoured at 1 s and carved around the bound compound (shown in black sticks). InhA protein is represented as a grey ribbon, with side chains of residues within 5 Å of bound compound displayed as sticks. The substrate binding loop is represented in orange. The middle column uses the same color code, and shows hydrogen bonds between bound compound and InhA or water molecules (represented as red spheres) as purple dashed lines. The orientation is rotated about 90° around the vertical axis. The right column summarizes in a table the structural features of each chain in the asymmetric unit of the crystal. Conformations of helices 6 (H6, residues 196-208) and 7 (H7, residues 209-226) are indicated as open (o), closed (c), disordered (d) (Rizet et al., 2025, ChemMedChem). Ligand binding in the specified chain is indicated with 1 (present) and 0 (absent) and the corresponding refined occupancy is indicated (n/a: not applicable). The conformation of Phe149 and Tyr148 is indicated as in or out (refs [3,4]). The chain highlighted in bold is shown in the figure.

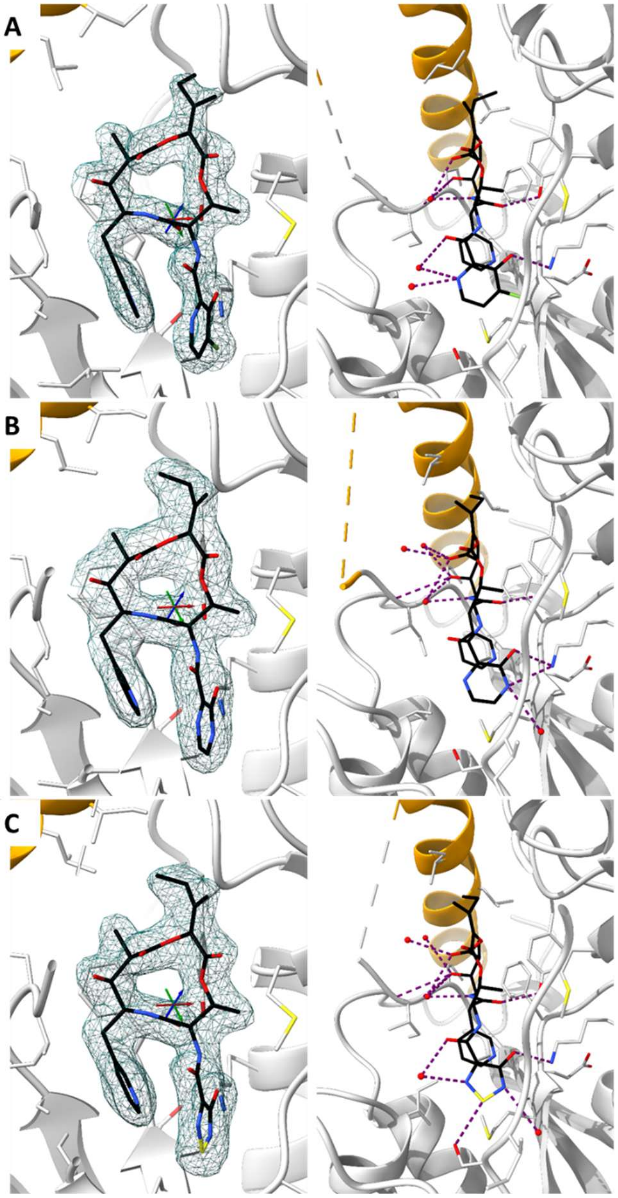

chain	H6	H7	lig.	occ.	F149	Y158
A	d/o	c	1	0.91	out	in
B	d	c	1	0.83	out	in
C	d/o	c	1	0.73	out	in
D	d	c	1	0.73	out	in
E	d	c	1	0.81	out	in
F	d	c	1	0.84	out	in

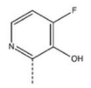

chain	H6	H7	lig.	occ.	F149	Y158
A	d	c	1	0.91	out	in
B	d	c	1	1.00	out	in
C	o	c	1	1.00	out	in
D	o	c	1	1.00	out	in

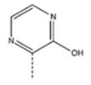

chain	H6	H7	lig.	occ.	F149	Y158
A	d	d/c	1	0.94	out	in
B	d	d/c	1	1.00	out	in
C	o	c	1	0.95	out	in
D	o	c	1	0.93	out	in

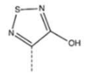

Figure S4): View of the interaction between the InhA active site and A) derivative **13** (PDB: 9RJM), B) derivative **14** (PDB: 9RJN), and C) derivative **15** (PDB: 9RJP). For each compound, the left column shows the final 2*m*Fo-*D*Fc electron density map as a mesh, contoured at 1 s and carved around the bound compound (shown in black sticks). InhA protein is represented as a grey ribbon, with side chains of residues within 5 Å of bound compound displayed as sticks. The substrate binding loop is represented in orange. The middle column uses the same color code, and shows hydrogen bonds between bound compound and InhA or water molecules (represented as red spheres) as purple dashed lines. The orientation is rotated about 90° around the vertical axis. The right column summarizes in a table the structural features of each chain in the asymmetric unit of the crystal. Conformations of helices 6 (H6, residues 196-208) and 7 (H7, residues 209-226) are indicated as open (o), closed (c), disordered (d) (Rizet et al., 2025, ChemMedChem). Ligand binding in the specified chain is indicated with 1 (present) and 0 (absent) and the corresponding refined occupancy is indicated (n/a: not applicable). The conformation of Phe149 and Tyr148 is indicated as in or out (refs [3,4]). The chain highlighted in bold is shown in the figure.

1.4 Crystallographic data collection and refinement statistics

Table S2: Crystallographic data collection and refinement statistics

	InhA-pyridomycine **1**		InhA-KV26a **4**		InhA-KV29a **5**		InhA-KV37a **9**		InhA-KV41a **11**		InhA-KV35a **12**		InhA-EP196 **13**		InhA-KV32a **14**		InhA-KV25a **15**	
PDBID	**9RJG**		**9RJH**		**9RJI**		**9RJJ**		**9RJK**		**9RJL**		**9RJM**		**9RJN**		**9RJP**	
	**Data collection and processing**																	
Beamline	ESRF, ID30A3		ALBA, XALOC		ALBA, XALOC		SOLEIL, PX1		ALBA, XALOC		ALBA, XALOC		ALBA, XALOC		ALBA, XALOC		ALBA, XALOC	
Spacegroup	C2		C2		C2		C2		$P2_1$		C2		C2		$P2_1$		$P2_1$	
Cell parameters																		
a, b, c (Å)	100.23, 83.50, 188.41		99.64, 92.29, 187.99		100.05, 82.14, 188.75		99.92, 82.54, 187.99		82.07, 81.35, 95.48		99.80, 82.35, 187.76		100.04, 82.58, 188.61		82.46, 81.30, 95.55		82.12, 81.18, 95.62	
?, ?, ? (°)	90.00, 95.89, 90.00		90.00, 95.84, 90.00		90.00, 95.79, 90.00		90.00, 95.79, 90.00		90.00, 99.414, 90.00		90.00, 95.67, 90.00		90.00, 95.94, 90.00		90.00, 99.53, 90.00		90.00, 99.89, 90.00	
Resolution range (Å)	62.47 – 1.71	1.74 – 1.71	187.0 – 1.71	1.74 – 1.71	63.35 – 1.77	1.80 – 1.77	187.03 – 2.08	2.12 – 2.08	94.19 – 1.66	1.69 – 1.66	63.39 – 1.70	1.73 – 1.70	187.60 – 1.99	2.02 – 1.99	94.23 – 2.50	2.54 – 2.50	61.50 – 2.15	2.18 – 2.15
No. observations	1,030,449	52,707	550,601	26,391	468,017	21,873	616,955	32,532	498,200	23,114	511,851	22,826	319,201	12,351	108,013	5,538	209,465	11,618
No. reflections	162,423	8,079	159,535	7,918	142,969	7,171	87,988	4,503	146,260	7,265	160,107	7,452	102,735	4,878	39,956	2,039	62,851	3,326
Multiplicity	6.3	6.5	3.5	3.3	3.3	3.1	7.0	7.2	3.4	3.2	3.23	3.1	3.1	2.5	2.7	2.7	3.3	3.5
Completeness (%)	98.8	99.5	98.1	97.7	95.6	95.8	96.3	99.9	99.7	99.7	96.2	90.5	97.3	91.8	91.9	95.1	92.4	98.9
<I/?>	12.6	2.2	9.0	2.3	7.8	2.1	8.9	1.7	9.4	1.4	9.6	2.2	11.8	2.0	6.1	1.8	8.8	1.7
R_{merge}	0.085	0.895	0.093	0.655	0.106	0.588	0.171	1.226	0.066	0.905	0.075	0.573	0.069	0.518	0.148	0.740	0.119	0.940
R_{pim}	0.039	0.379	0.080	0.567	0.068	0.396	0.069	0.486	0.042	0.592	0.049	0.0488	0.062	0.453	0.104	0.512	0.077	0.593
CC(1/2)	0.998	0.637	0.994	0.612	0.990	0.635	0.996	0.643	0.995	0.775	0.996	0.643	0.997	0.644	0.986	0.634	0.994	0.653
	**Structure refinement**																	
Resolution range (Å)	62.50 – 1.71		25.06 – 1.71		25.86 – 1.77		30.46 – 2.08		94.20 – 1.66		63.40 – 1.70		187.60 – 1.99		36.56 – 2.50		31.40 – 2.15	
No. Reflections (work/free)	151,295 / 8,121		151,500 / 7,986		135,814 / 7,118		83,531 / 4,424		138,859 / 7,362		152,111 / 7,996		97,599 / 5,135		37,969 / 1,964		59,561 / 3,092	
R_{work}/R_{free}	0.1724 / 0.1944		0.1749 / 0.1980		0.2182 / 0.2355		0.1853 / 0.2180		0.1855 / 0.2050		0.1937 / 0.2161		0.1995 / 0.2267		0.2168 / 0/2487		0.2098 / 0.2316	
No. of non-H atoms																		
Protein	11,491		11,626		11,287		11,675		7,565		11,445		11,392		7,693		7,789	
Ligand[a]	234		195		195		200		174		200		240		156		152	
Solvent	1,128		1,269		856		864		816		1,114		856		289		555	
Rms deviation																		
Bond lengths (Å)	0.0090		0.0090		0.0085		0.0077		0.0094		0.0067		0.0079		0.0069		0.0065	
Bond angles (°)	0.94		0.95		0.85		0.90		0.97		0. 87		0.92		0.88		0.87	
Ramachandran																		
Most favoured (%)	96.7		97.1		96.5		96.9		96.6		96.6		96.9		96.1		96.8	
Allowed (%)	3.3		2.8		3.4		3.1		3.4		3.3		3.1		3.8		3.2	
Outliers (%)	0.0		0.1		0.0		0.0		0.0		0.1		0.0		0.1		0.0	

[a] Ligand refers only to pyridomycin or its derivative. Other molecules, such as polyethylene glycol, are considered as solvent.

2. NMR, HRMS and HPLC Spectra of compounds 2-15 and S3-S6

Compound 2

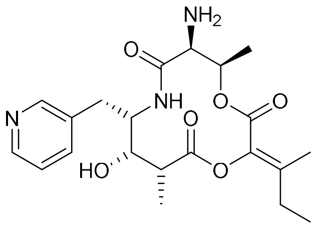

3

Chemical Formula: $C_{21}H_{29}N_3O_6$
Exact Mass: 419.21
Molecular Weight: 419.48

Compound 2: ^{1}H-NMR (300 MHz, MeOD-d$_4$)

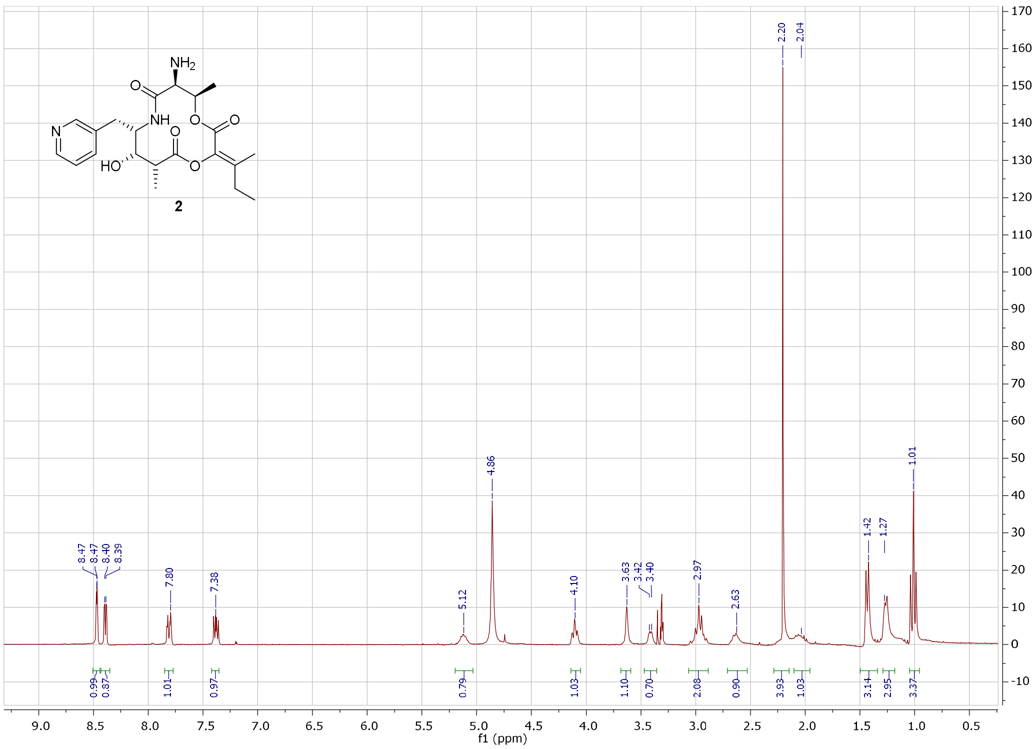

Compound 3 (internal batch code KV28a)

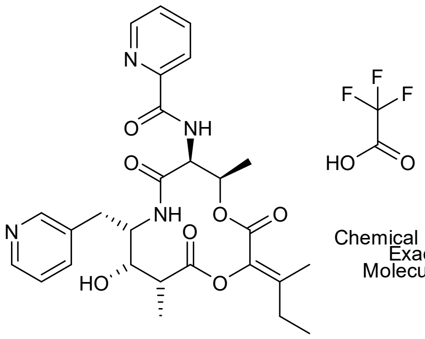

Chemical Formula: $C_{27}H_{32}N_4O_7$
Exact Mass: 524.23
Molecular Weight: 524.57

3

Compound **3**, (internal batch code KV28a): HPLC-UV purity (254 nm)

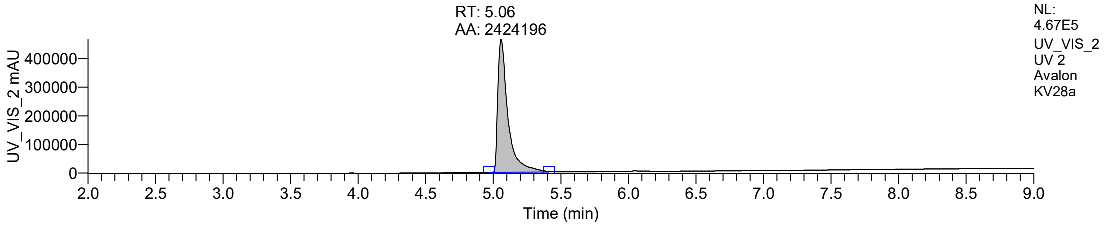

Compound **3**, (internal batch code KV28a): HRMS

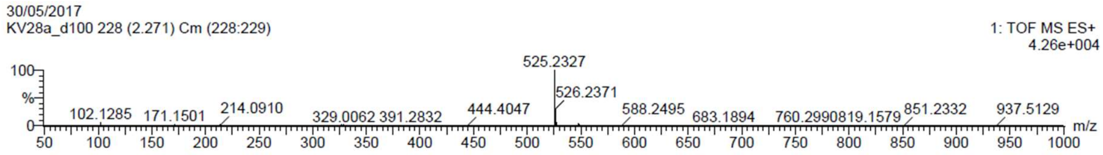

Minimum:				-1.5		
Maximum:		10.0	5.0	50.0		
Mass	Calc. Mass	mDa	PPM	DBE	i-FIT	Formula
525.2327	525.2349	-2.2	-4.2	13.5	43.6	C27 H33 N4 O7

Compound 3, (internal batch code KV28a): ^{1}H NMR (600 MHz, $(CD_3)_2SO$)

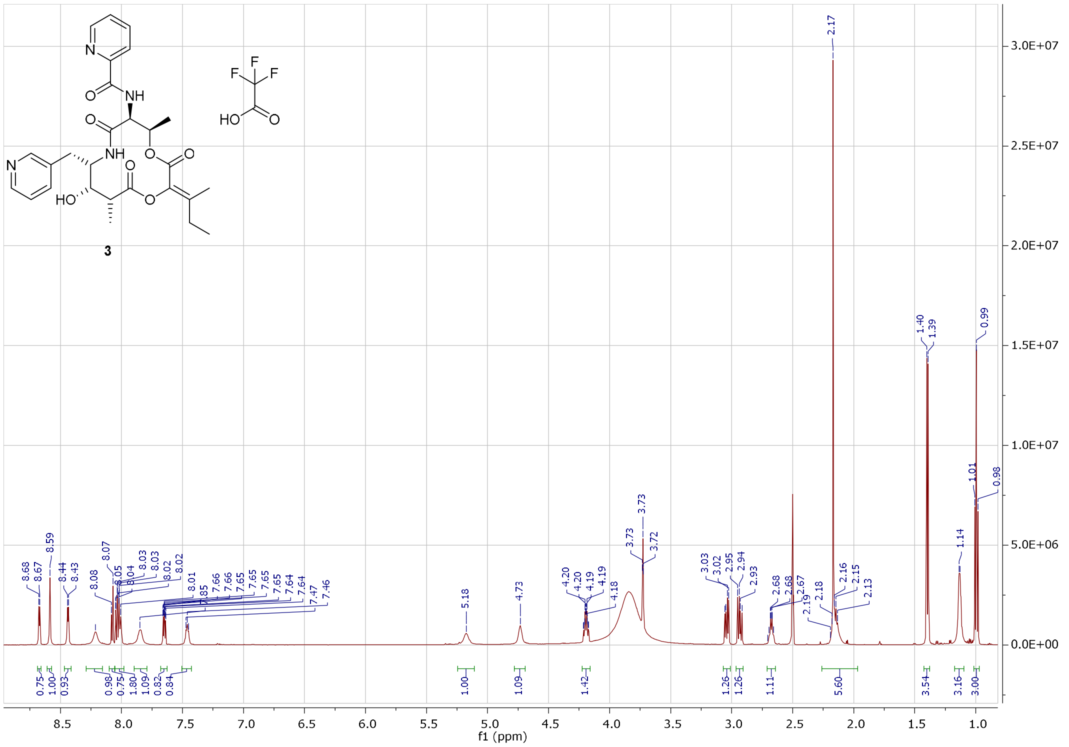

Compound 4 (internal batch code KV26a)

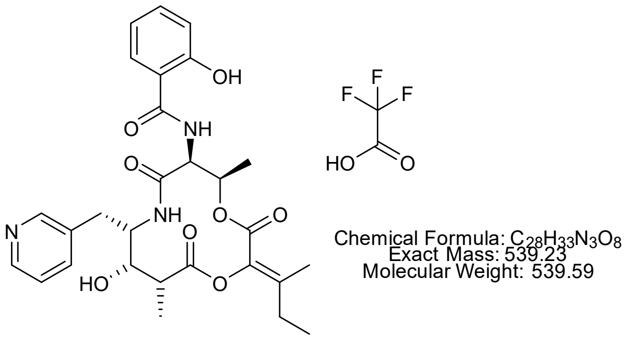

4

Compound **4**, (internal batch code KV26a): HPLC-UV purity (254 nm)

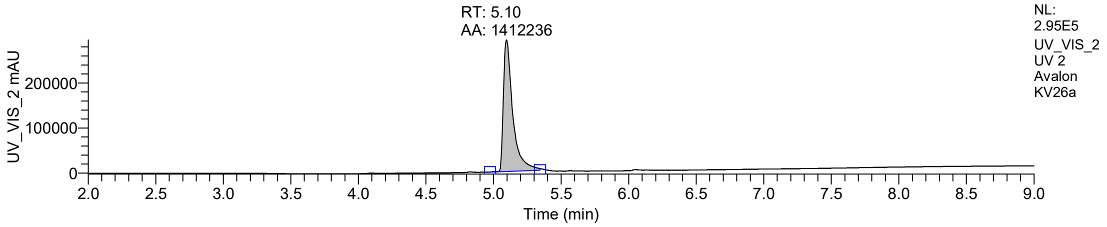

Compound **4**, (internal batch code KV26a): HRMS

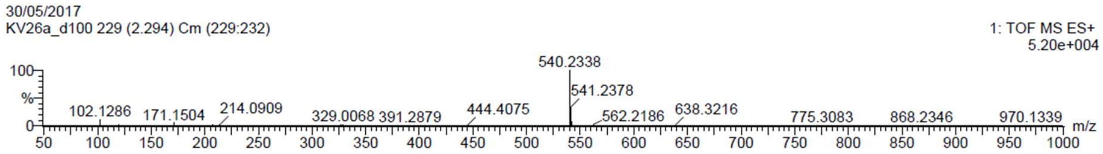

Minimum:				-1.5		
Maximum:		10.0	5.0	50.0		
Mass	Calc. Mass	mDa	PPM	DBE	i-FIT	Formula
540.2338	540.2346	-0.8	-1.5	13.5	15.4	C28 H34 N3 O8

Compound **4**, (internal batch code KV26a): ^{1}H NMR (600 MHz, $(CD_3)_2SO$)

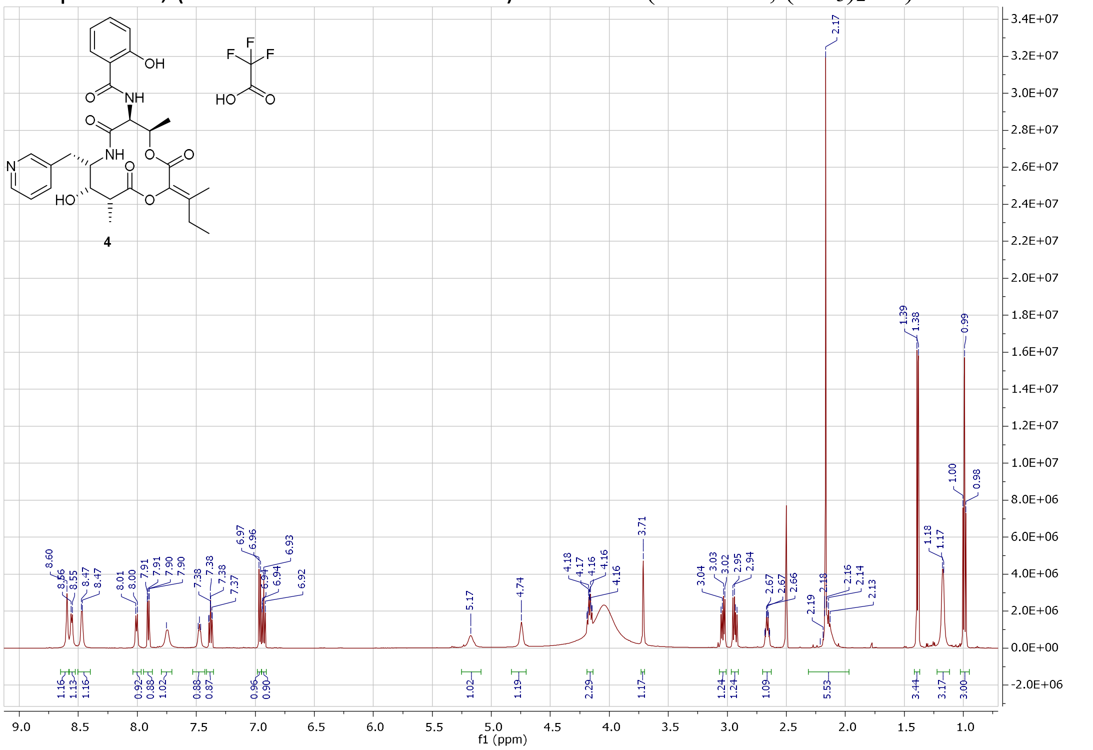

Compound 5 (internal batch code KV29a)

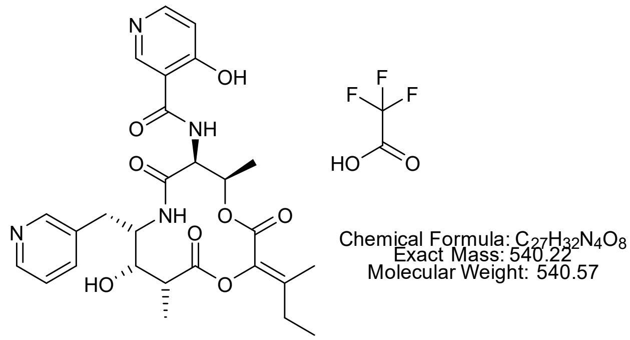

5

Compound **5** (internal batch code KV29a): HPLC-UV purity (254 nm)

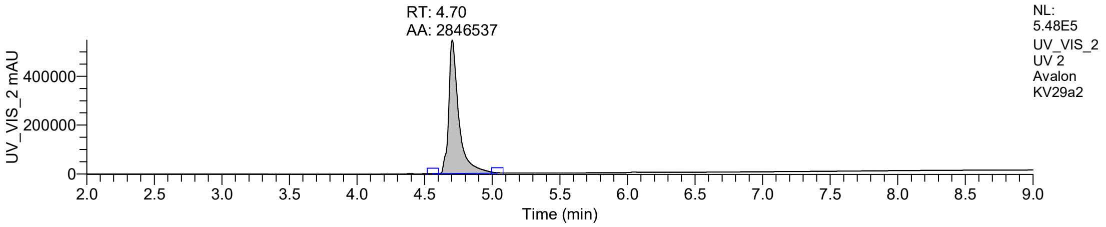

Compound **5** (internal batch code KV29a): HRMS

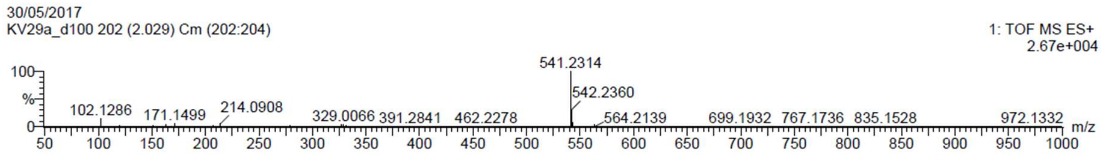

Minimum:				-1.5		
Maximum:		10.0	5.0	50.0		
Mass	Calc. Mass	mDa	PPM	DBE	i-FIT	Formula
541.2314	541.2298	1.6	3.0	13.5	6.0	C27 H33 N4 O8

Compound **5** (internal batch code KV29a): ^{1}H NMR (600 MHz, $(CD_3)_2SO$)

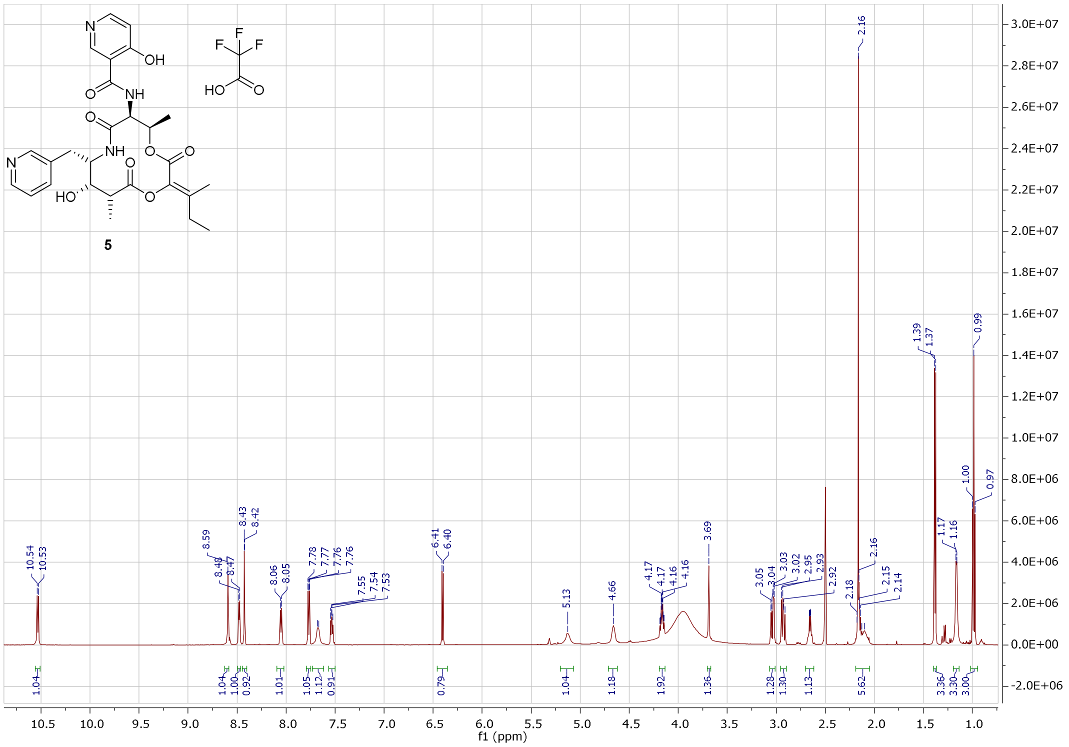

Compound 6 (internal batch code KV27a)

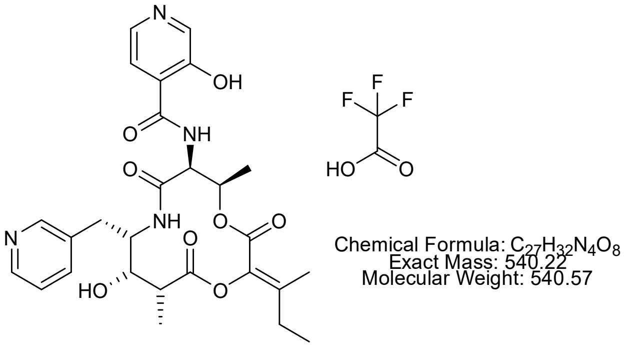

6

Compound **6** (internal batch code KV27a): HPLC-UV purity (254 nm)

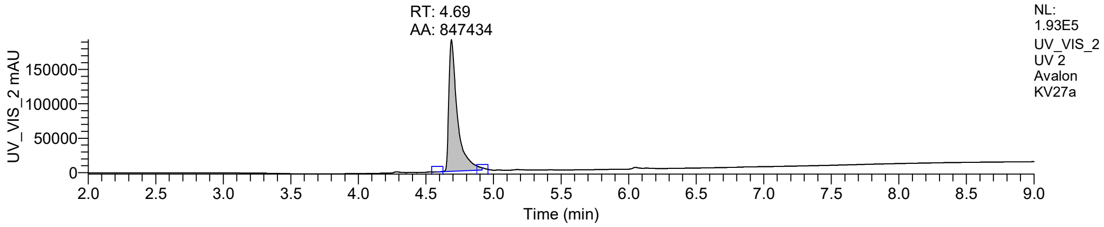

Compound **6** (internal batch code KV27a): HRMS

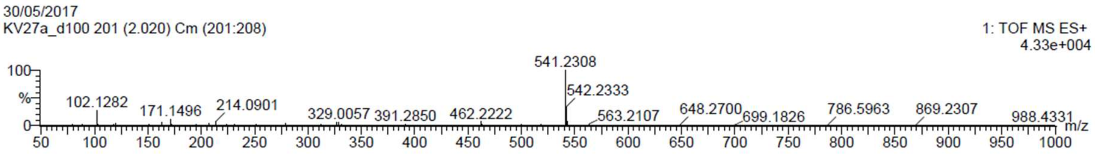

Minimum:				-1.5		
Maximum:		10.0	5.0	50.0		
Mass	Calc. Mass	mDa	PPM	DBE	i-FIT	Formula
541.2308	541.2298	1.0	1.8	13.5	29.0	C27 H33 N4 O8

Compound **6** (internal batch code KV27a): ^{1}H NMR (600 MHz, $(CD_3)_2SO$)

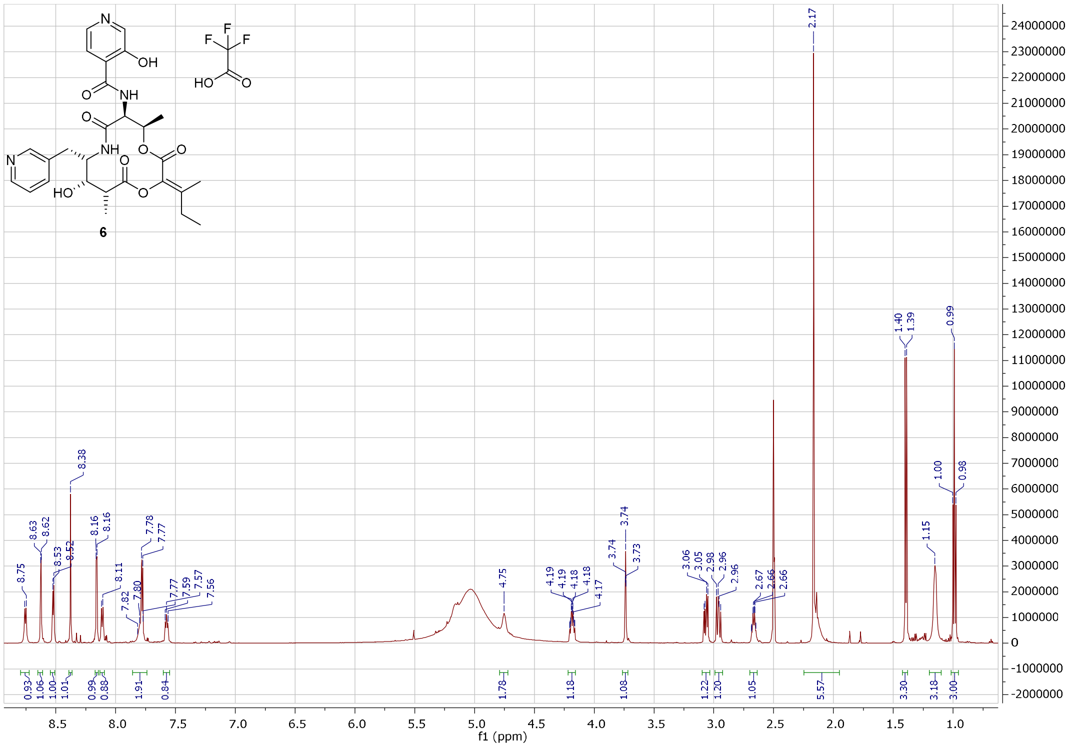

Compound 7 (internal batch code KV08a)

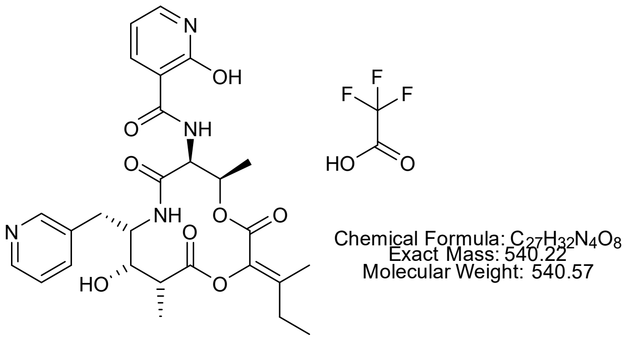

7

Compound **7** (internal batch code KV08a): HPLC-UV purity (254 nm)

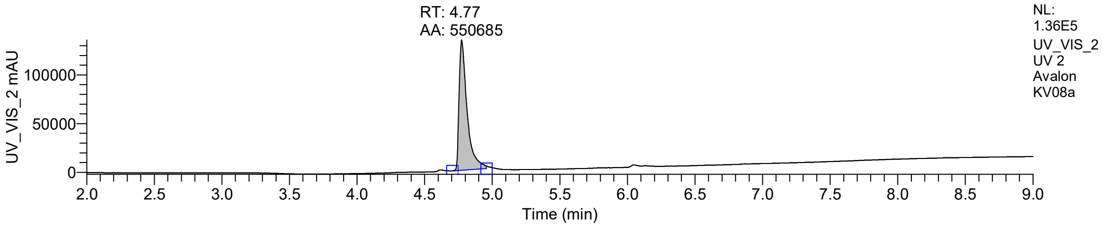

Compound **7** (internal batch code KV08a): HRMS

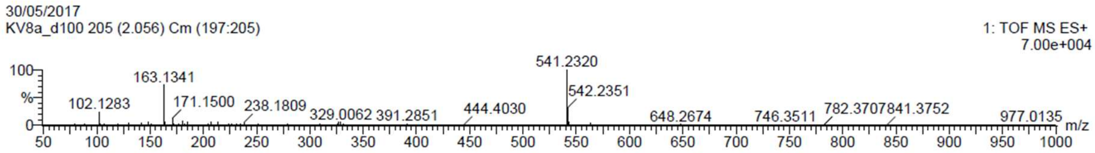

	Mass	Calc. Mass	mDa	PPM	DBE	i-FIT	Formula
Minimum:					-1.5		
Maximum:			10.0	5.0	50.0		
	541.2320	541.2298	2.2	4.1	13.5	35.7	C27 H33 N4 O8

Compound **7** (internal batch code KV08a): ^{1}H NMR (600 MHz, $(CD_3)_2SO$)

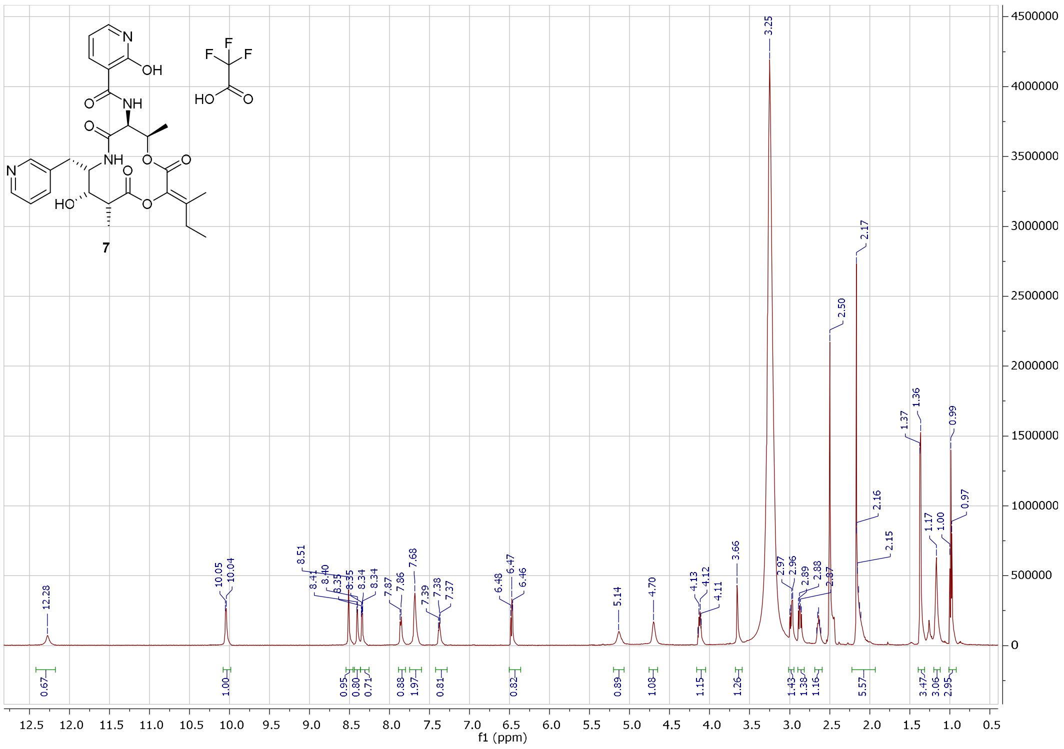

Compound 8 (internal batch code KV31a)

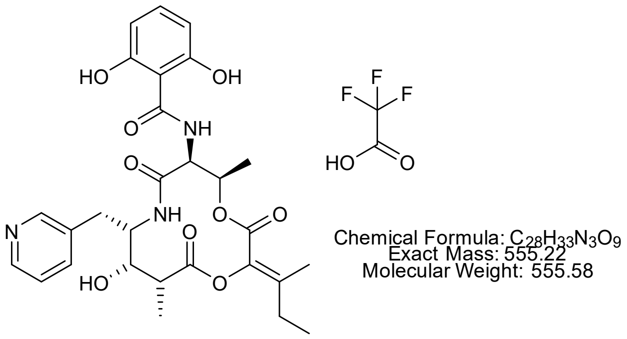

8

Compound **8** (internal batch code KV31a): HPLC-UV purity (254 nm)

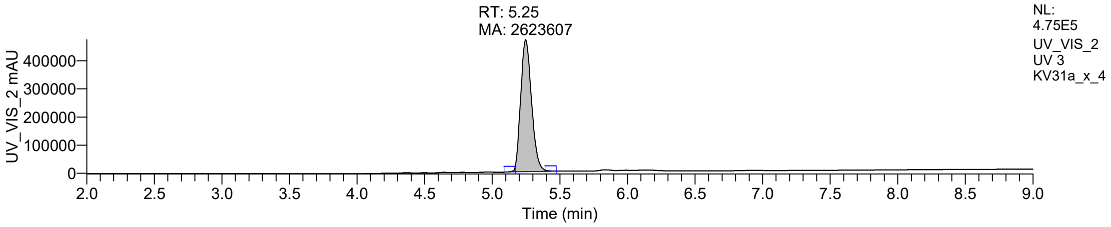

Compound **8** (internal batch code KV31a): HRMS

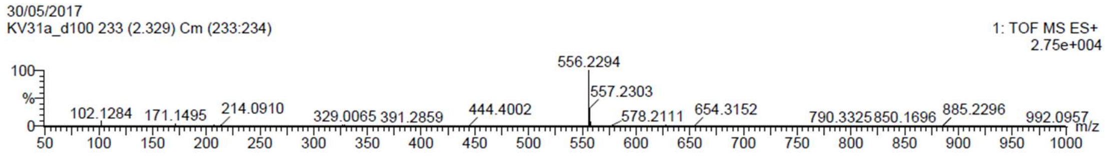

Minimum:				-1.5		
Maximum:		10.0	5.0	50.0		
Mass	Calc. Mass	mDa	PPM	DBE	i-FIT	Formula
556.2294	556.2295	-0.1	-0.2	13.5	4.7	C28 H34 N3 O9

Compound **8** (internal batch code KV31a): ^{1}H NMR (600 MHz, $(CD_3)_2SO$)

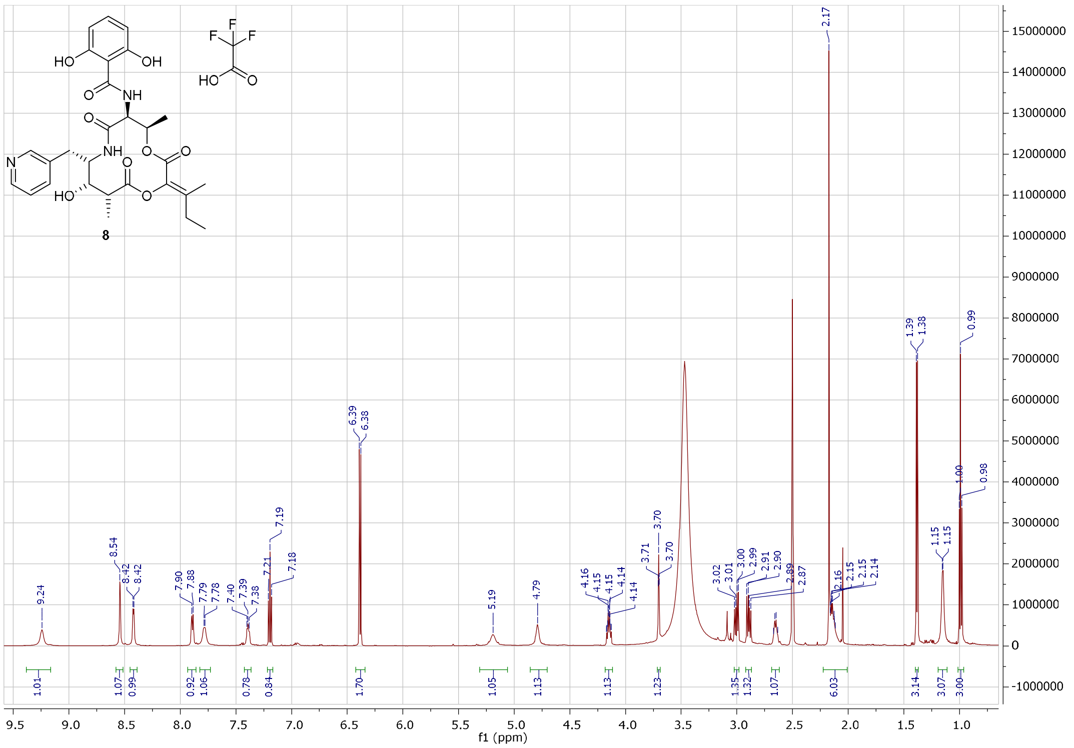

Compound 9 (internal batch code KV37a)

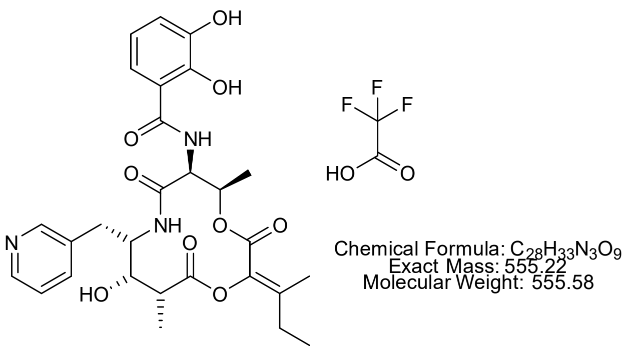

9

Compound **9** (internal batch code KV37a): HPLC-UV purity (254 nm)

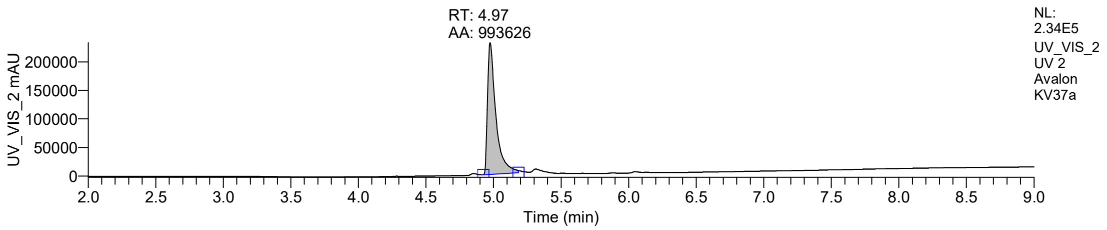

Compound **9** (internal batch code KV37a): HRMS

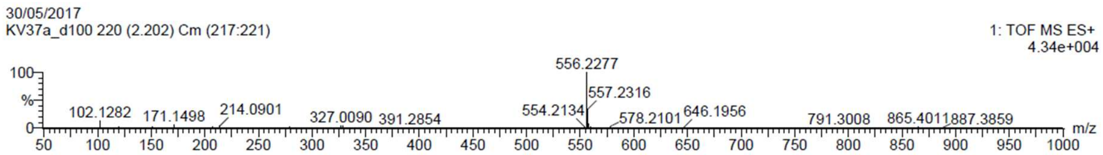

Minimum:				-1.5		
Maximum:		10.0	5.0	50.0		
Mass	Calc. Mass	mDa	PPM	DBE	i-FIT	Formula
556.2277	556.2295	-1.8	-3.2	13.5	41.3	C28 H34 N3 O9

Compound **9** (internal batch code KV37a): ^{1}H NMR (600 MHz, $(CD_3)_2SO$)

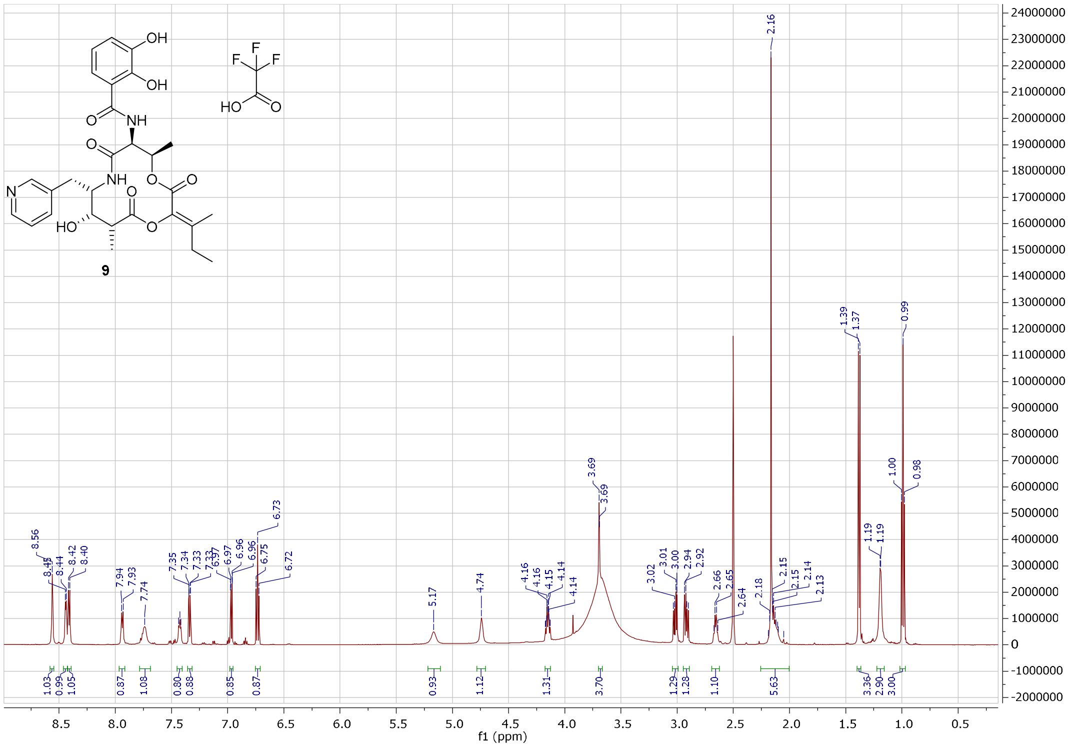

Compound 10 (internal batch code KV40a)

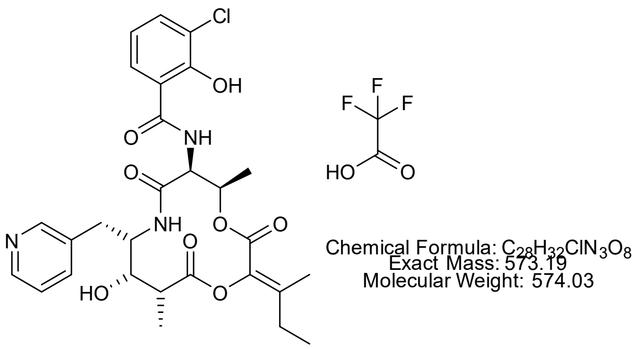

10

Compound **10** (internal batch code KV40a): HPLC-UV purity (254 nm)

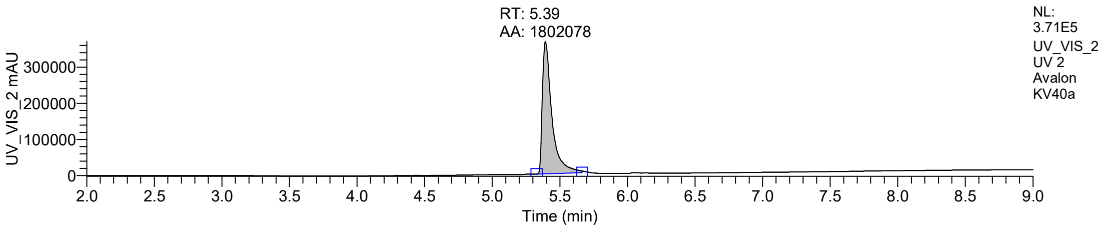

Compound **10** (internal batch code KV40a): HRMS

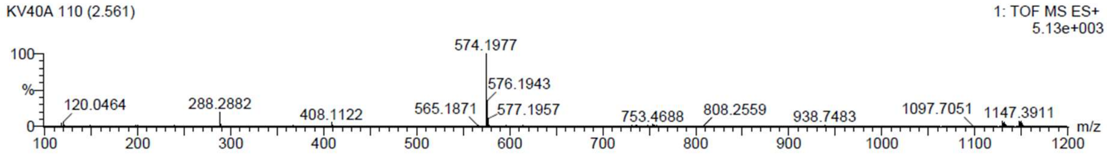

Minimum:				-1.5		
Maximum:		5.0	10.0	50.0		
Mass	Calc. Mass	mDa	PPM	DBE	i-FIT	Formula
574.1977	574.1956	2.1	3.7	13.5	6.3	C28 H33 N3 O8 Cl

Compound **10** (internal batch code KV40a): ^{1}H NMR (600 MHz, $(CD_3)_2SO$)

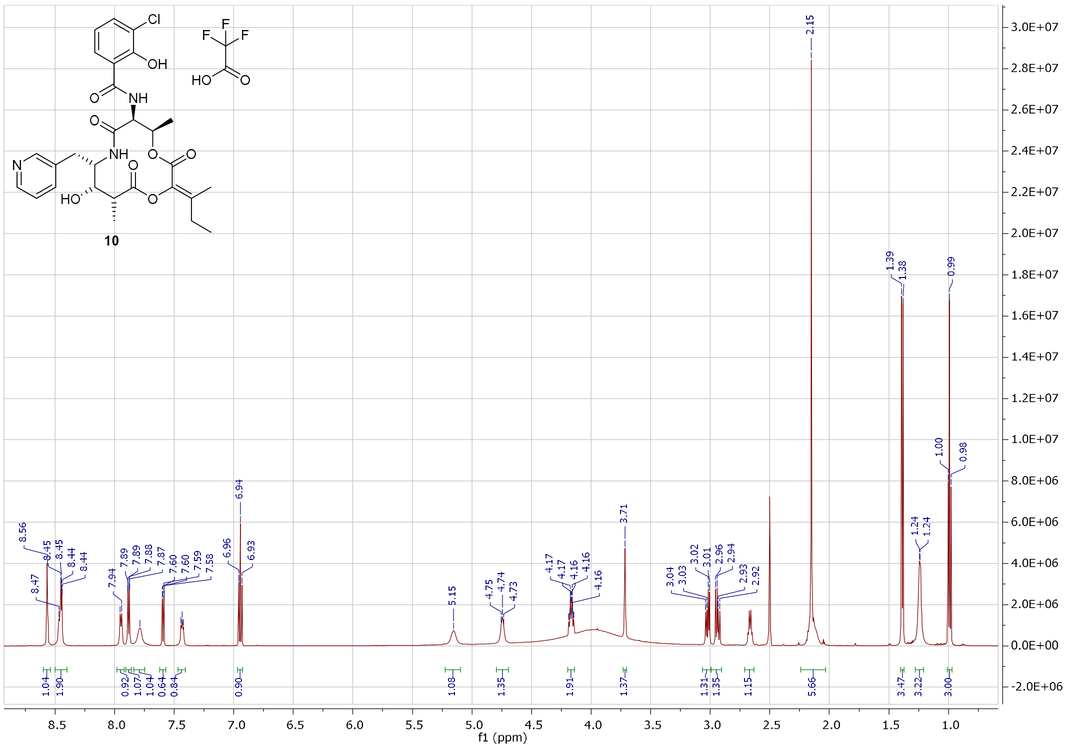

Compound 11 (internal batch code KV41a)

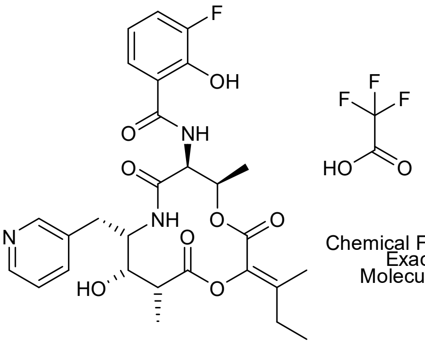

Chemical Formula: $C_{28}H_{32}FN_3O_8$
Exact Mass: 557.22
Molecular Weight: 557.58

11

Compound **11** (internal batch code KV41a): HPLC-UV purity (254 nm)

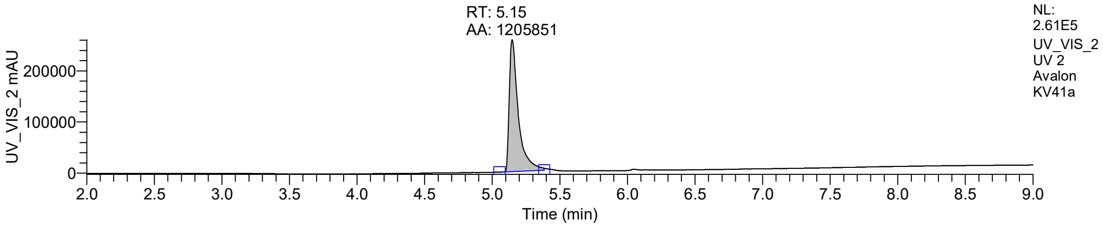

Compound **11** (internal batch code KV41a): HRMS

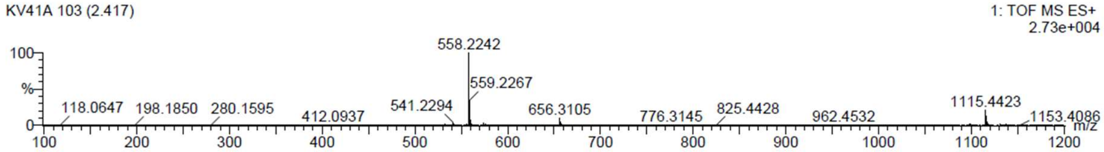

Minimum:				-1.5		
Maximum:		5.0	10.0	50.0		
Mass	Calc. Mass	mDa	PPM	DBE	i-FIT	Formula
558.2242	558.2252	-1.0	-1.8	13.5	12.6	C28 H33 N3 O8 F

Compound **11** (internal batch code KV41a): ^{1}H NMR (600 MHz, $(CD_3)_2SO$)

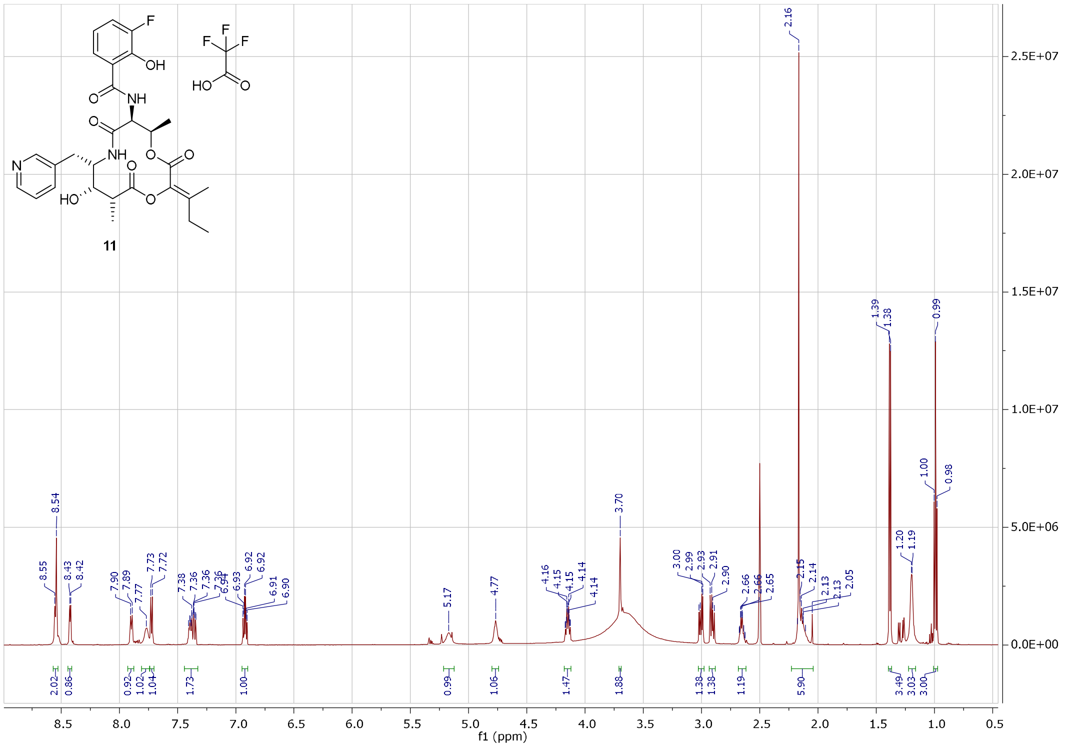

Compound 12 (internal batch code KV35a)

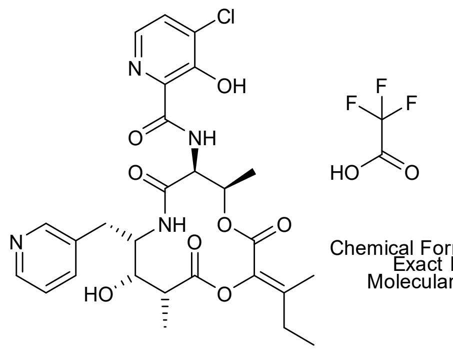

Chemical Formula: $C_{27}H_{31}ClN_4O_8$
Exact Mass: 574.18
Molecular Weight: 575.02

12

Compound **12** (internal batch code KV35a): HPLC-UV purity (254 nm)

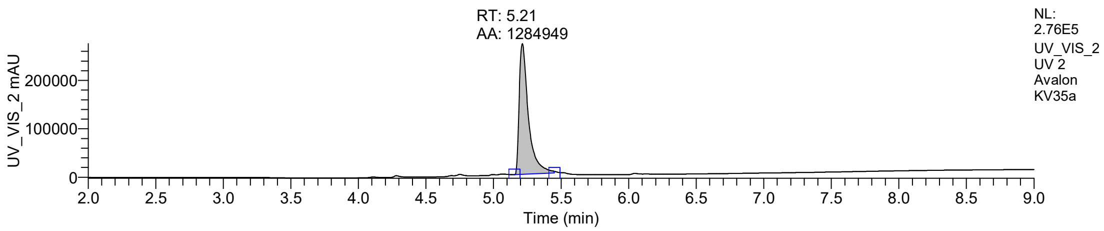

Compound **12** (internal batch code KV35a): HRMS

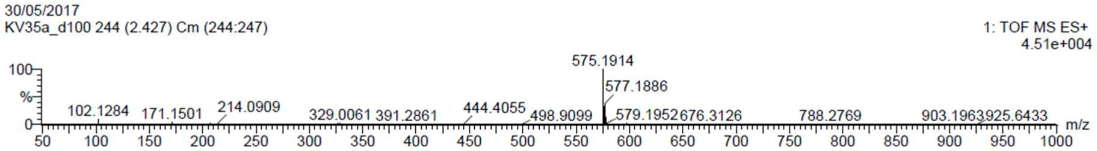

Minimum:				-1.5		
Maximum:		10.0	5.0	50.0		
Mass	Calc. Mass	mDa	PPM	DBE	i-FIT	Formula
575.1914	575.1909	0.5	0.9	13.5	2.7	C27 H32 N4 O8 Cl

Compound **12** (internal batch code KV35a): ^{1}H NMR (600 MHz, $(CD_3)_2SO$)

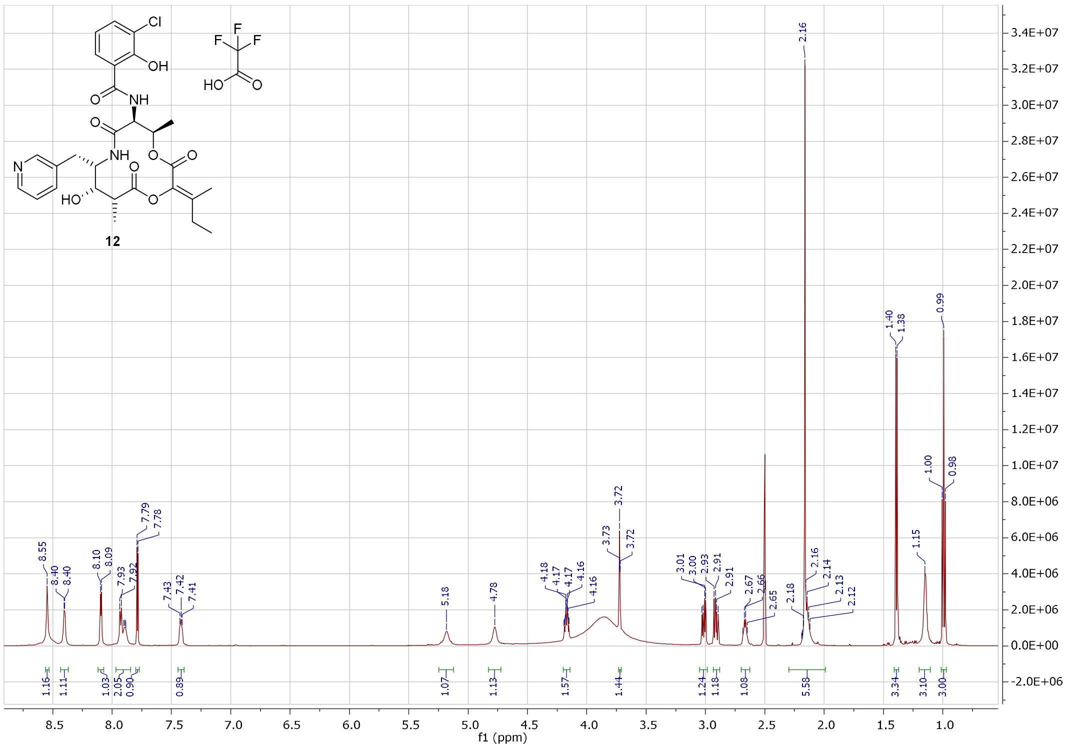

Compound 13 (internal batch code EP196)

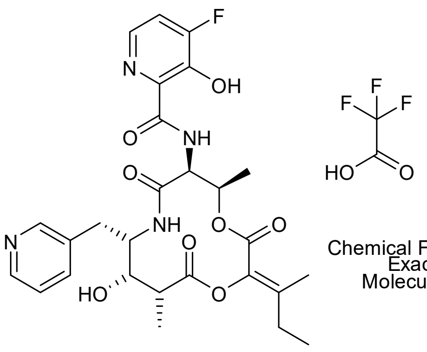

Chemical Formula: $C_{27}H_{31}FN_4O_8$
Exact Mass: 558.21
Molecular Weight: 558.56

13

Compound **13** (internal batch code EP196): HPLC-UV purity (254 nm)

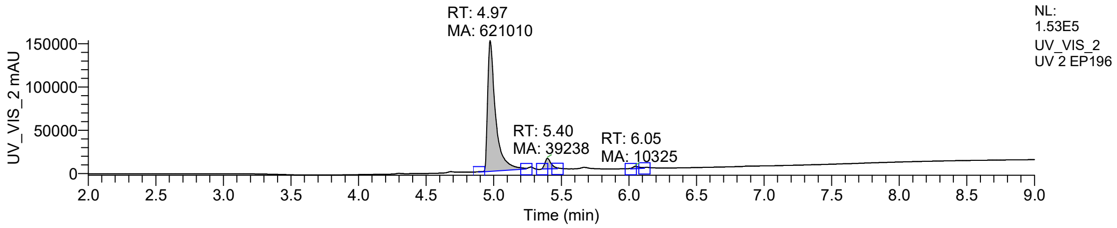

Compound **13** (internal batch code EP196): HRMS

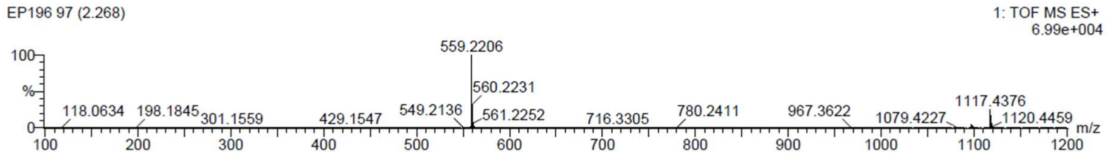

Minimum:				-1.5		
Maximum:		5.0	10.0	50.0		
Mass	Calc. Mass	mDa	PPM	DBE	i-FIT	Formula
559.2206	559.2204	0.2	0.4	13.5	1.0	C27 H32 N4 O8 F

Compound **13** (internal batch code EP196): ^{1}H NMR (500 MHz, CD_3OD) (top) and ^{13}C NMR (126 MHz, CD_3OD) (bottom)

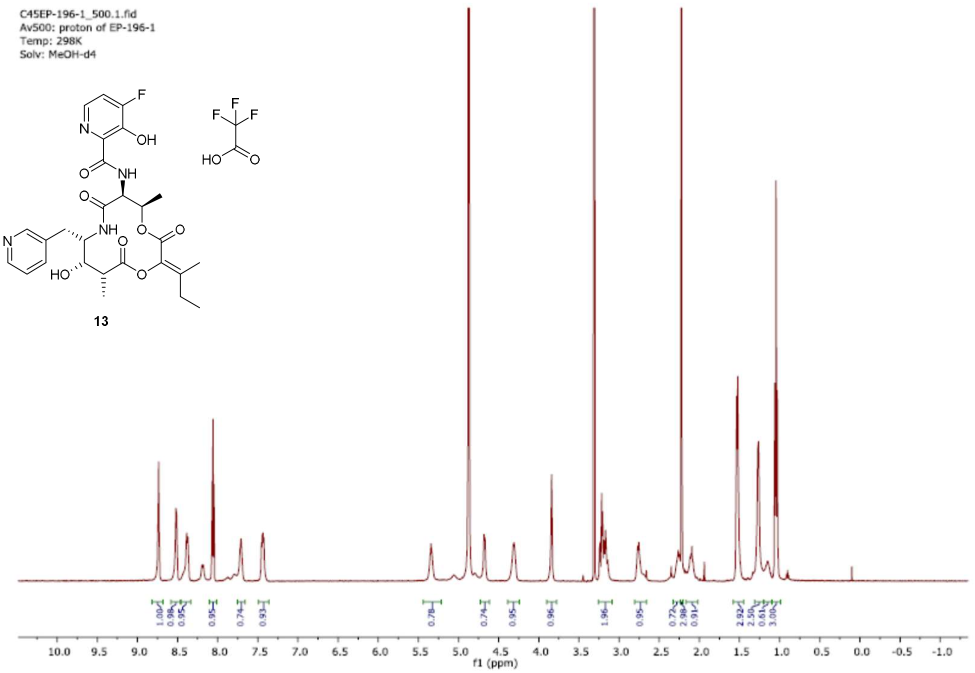

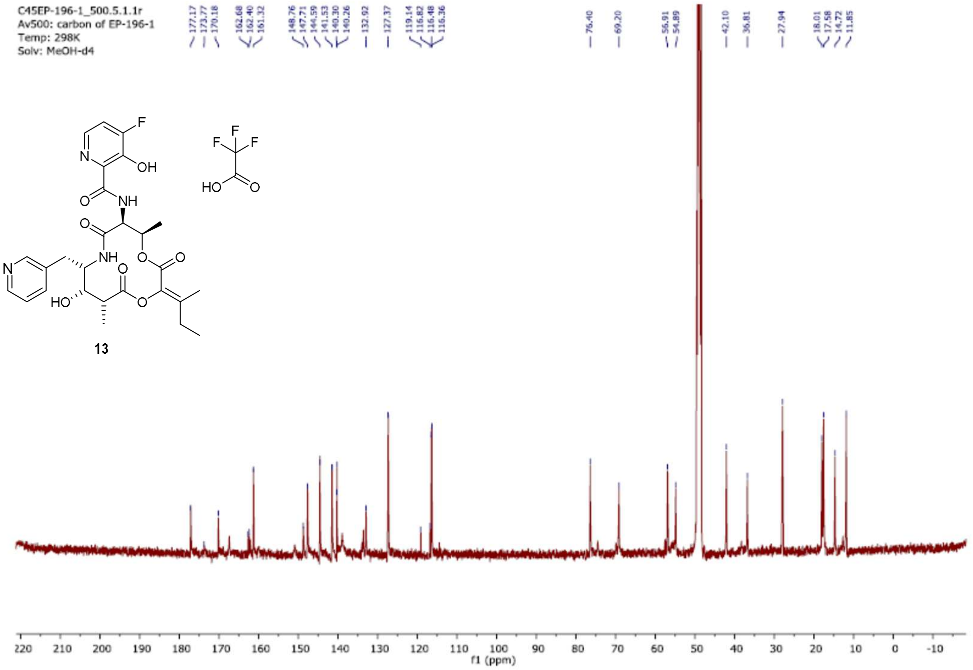

Compound 14 (internal batch code KV32a/DM458)

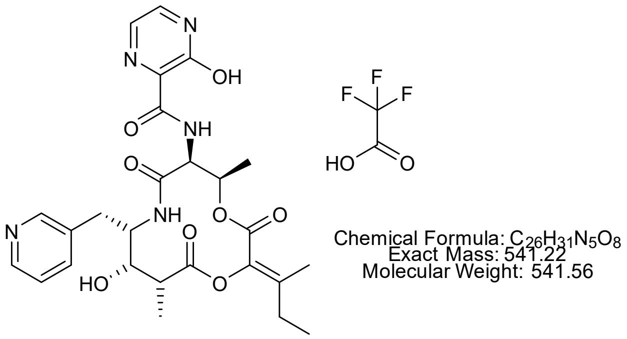

14

Compound **14** (internal batch code KV32a/DM456): HPLC-UV purity (254 nm)

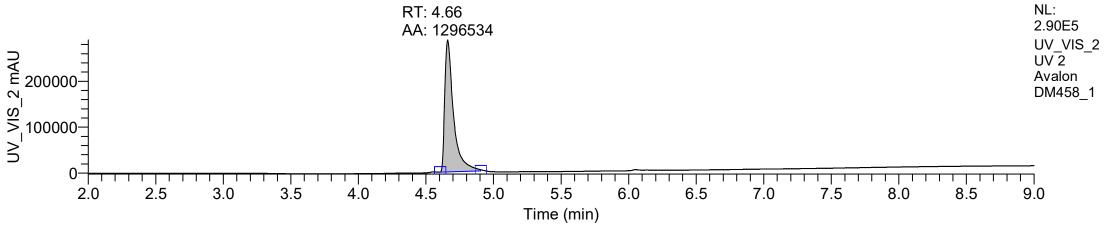

Compound **14** (internal batch code KV32a/DM456): HRMS

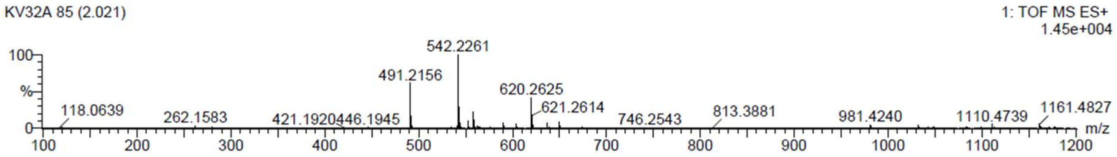

Minimum:				-1.5		
Maximum:		5.0	10.0	50.0		
Mass	Calc. Mass	mDa	PPM	DBE	i-FIT	Formula
542.2261	542.2251	1.0	1.8	13.5	11.5	C26 H32 N5 O8

Compound **14** (internal batch code KV32a/DM456): ^{1}H NMR (500 MHz, CD_3OD)

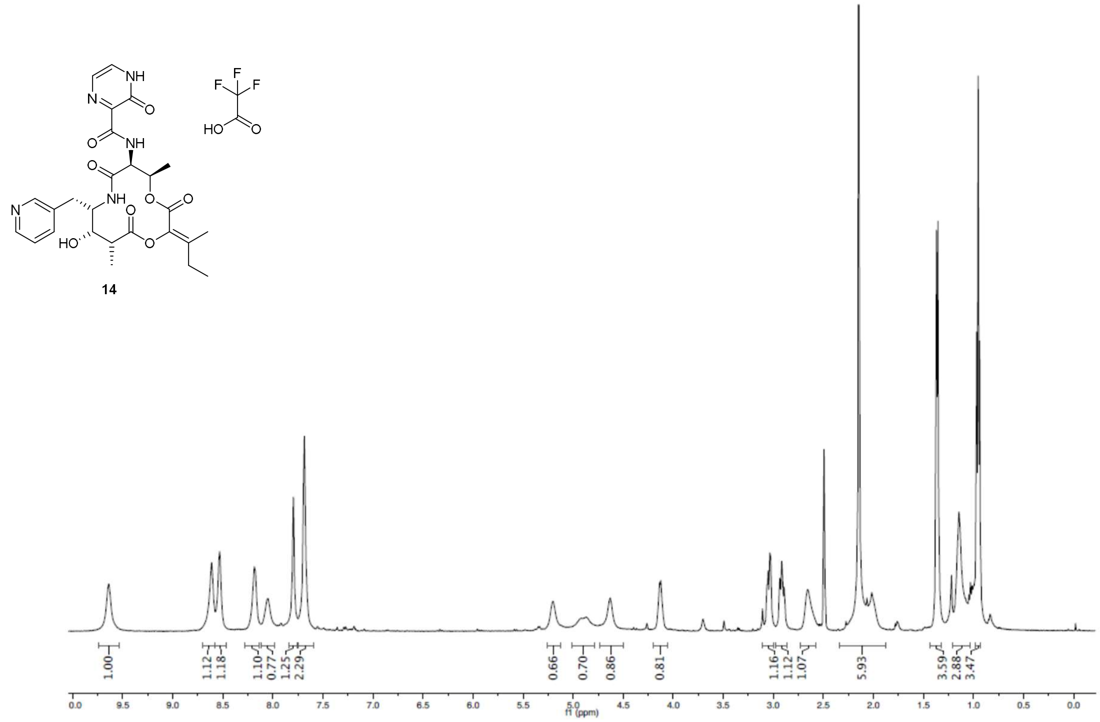

Compound 15 (internal batch code KV25a)

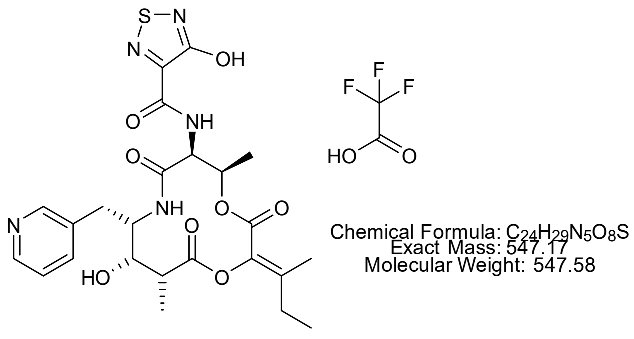

15

Compound **15** (internal batch code KV25a): HPLC-UV purity (254 nm)

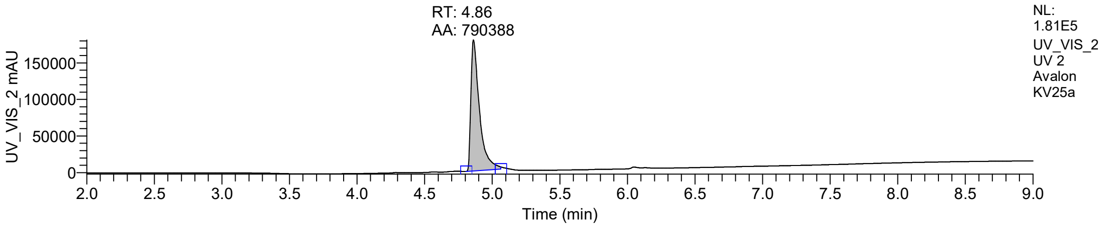

Compound **15** (internal batch code KV25a): HRMS

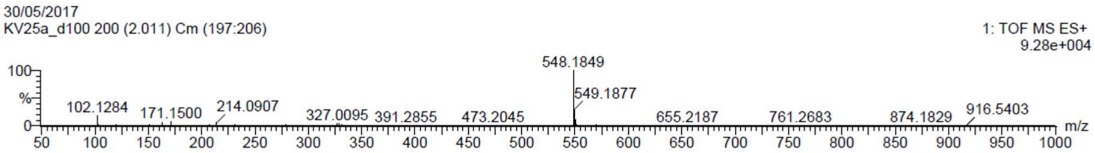

Minimum:				-1.5		
Maximum:		10.0	5.0	50.0		
Mass	Calc. Mass	mDa	PPM	DBE	i-FIT	Formula
548.1849	548.1815	3.4	6.2	12.5	54.2	C24 H30 N5 O8 S

Compound **15** (internal batch code KV25a): ^{1}H NMR (600 MHz, $(CD_3)_2SO$)

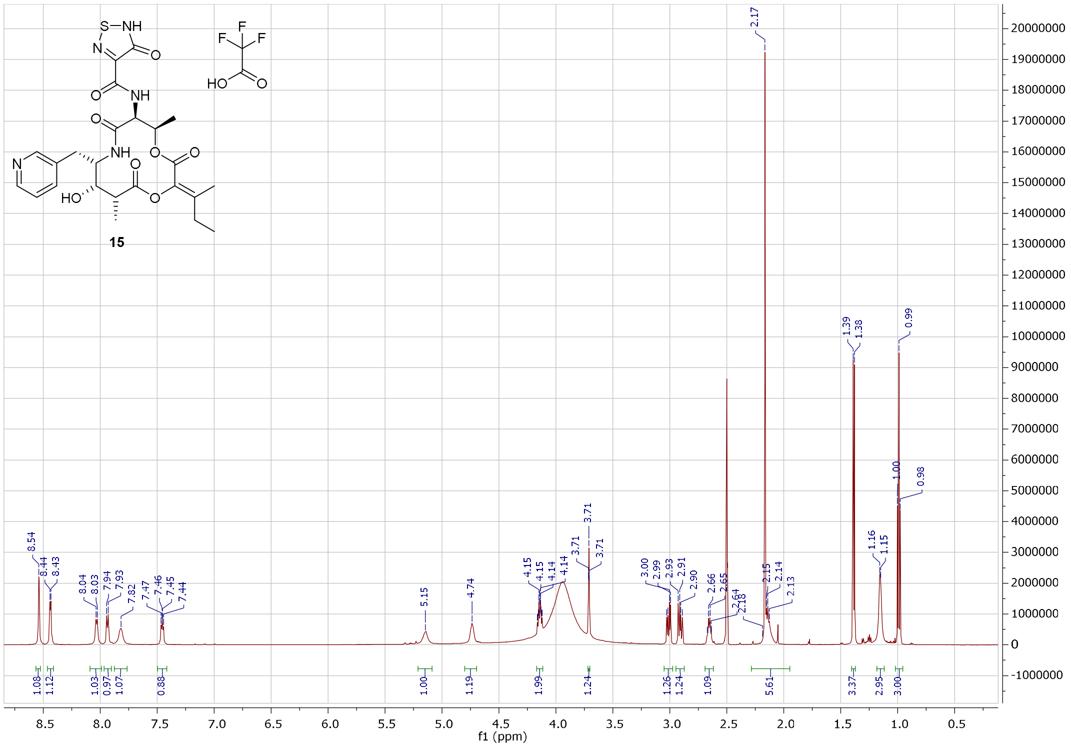

Compound S3: ^{1}H NMR (500 MHz, $(CD_3)_2SO$):

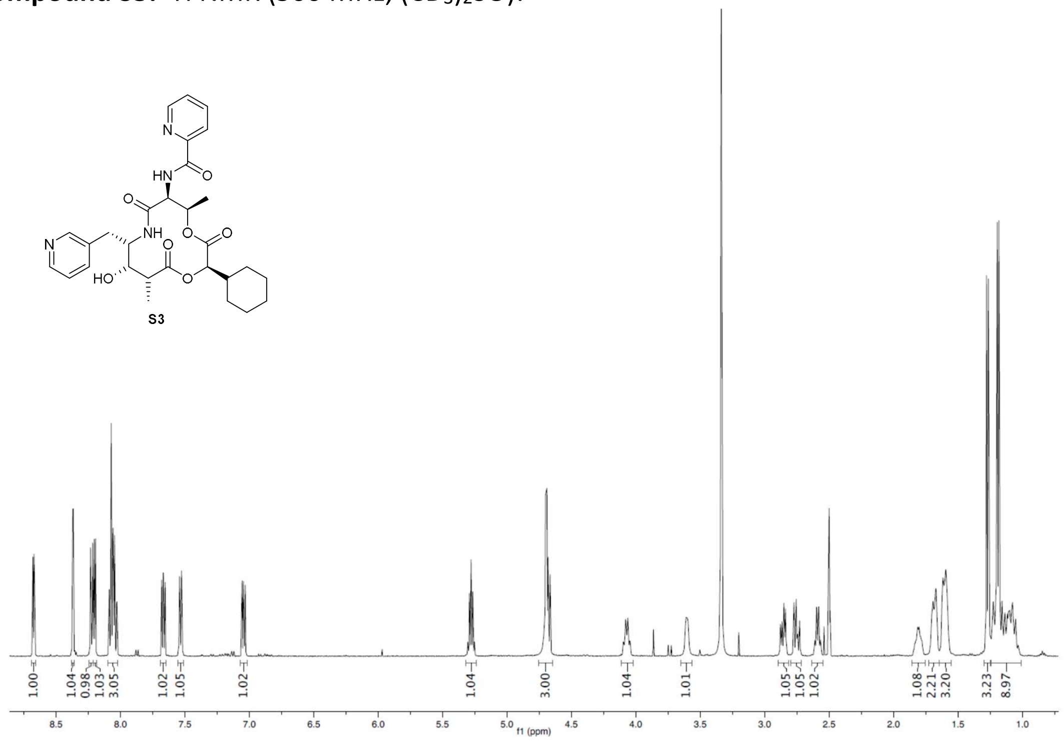

Compound S4: ^{1}H NMR (500 MHz, $(CD_3)_2SO$):

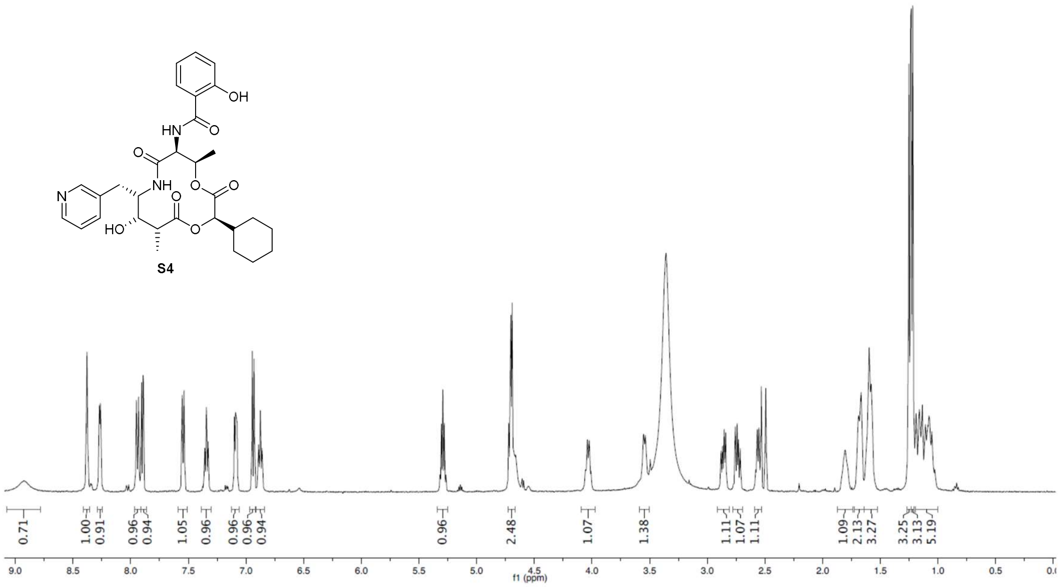

Compound S5: ^{1}H NMR (500 MHz, $(CD_3)_2SO$):

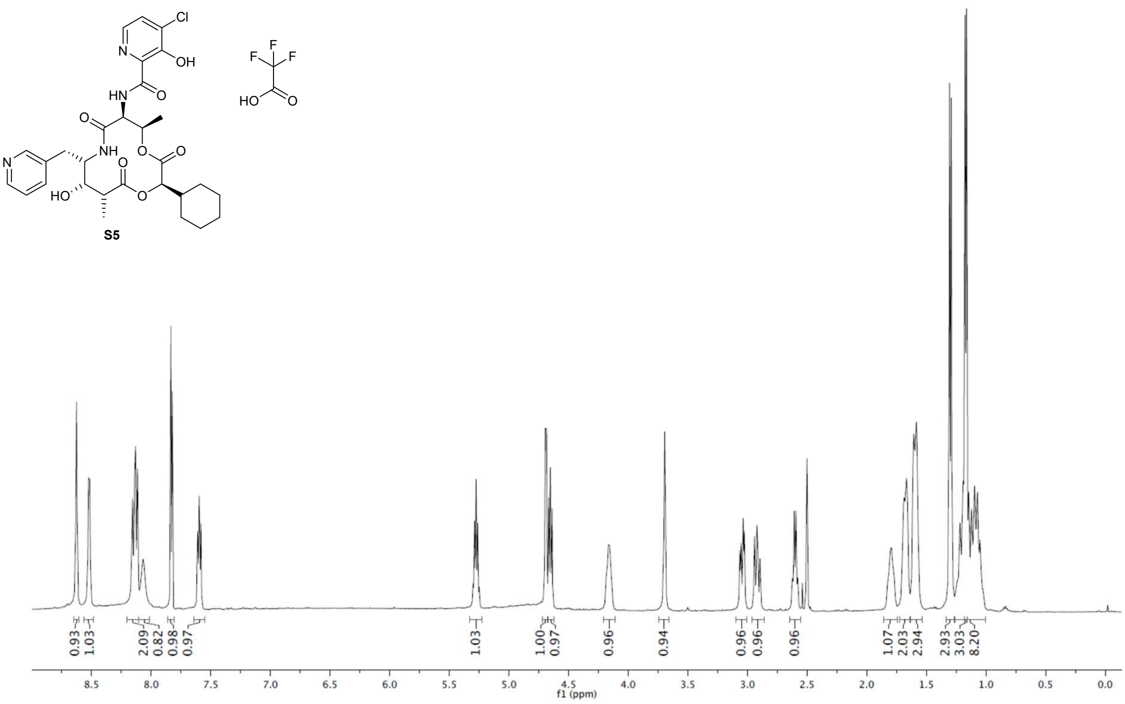

Compound S6: ^{1}H NMR (500 MHz, $(CD_3)_2SO$):

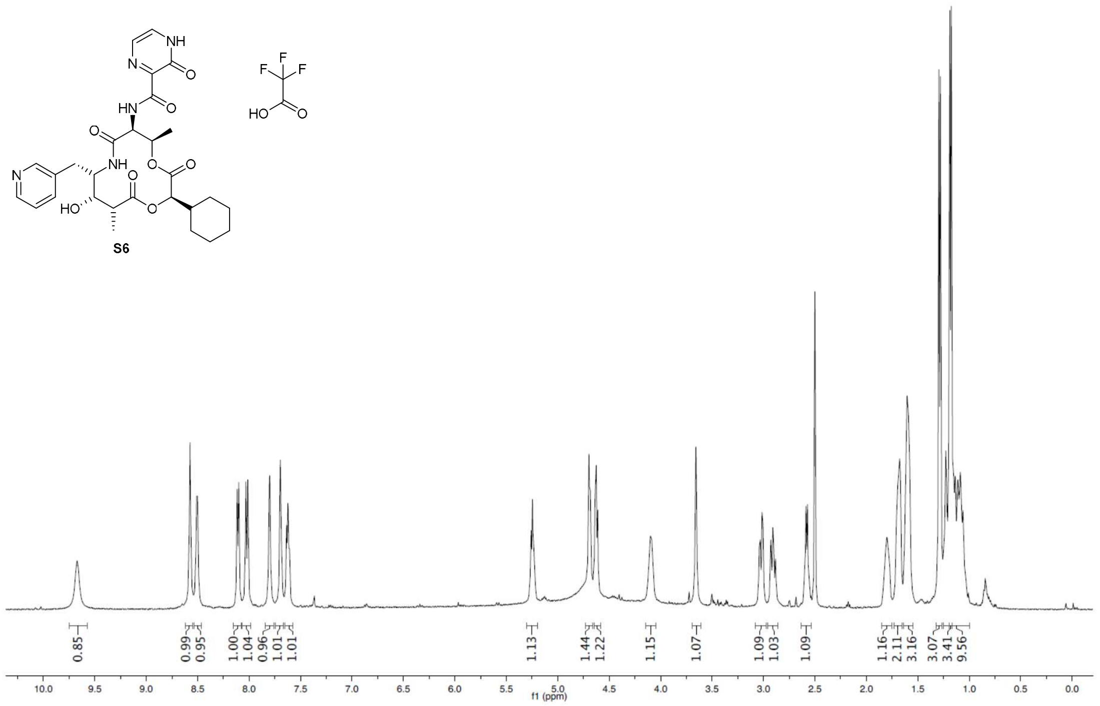

3. References

(1) Kienle, M.; Eisenring, P.; Stoessel, B.; Horlacher, O. P.; Hasler, S.; van Colen, G.; Hartkoorn, R. C.; Vocat, A.; Cole, S. T.; Altmann, K.-H. Synthesis and Structure–Activity Relationship Studies of C2-Modified Analogs of the Antimycobacterial Natural Product Pyridomycin. *J. Med. Chem.* **2020**, *63* (3), 1105–1131. https://doi.org/10.1021/acs.jmedchem.9b01457.

(2) Hajian, B.; Scocchera, E.; Shoen, C.; Krucinska, J.; Viswanathan, K.; G-Dayanandan, N.; Erlandsen, H.; Estrada, A.; Mikušová, K.; Korduláková, J.; Cynamon, M.; Wright, D. Drugging the Folate Pathway in Mycobacterium Tuberculosis: The Role of Multi-Targeting Agents. *Cell Chem. Biol.* **2019**, *26* (6), 781-791.e6. https://doi.org/10.1016/j.chembiol.2019.02.013.

(3) Rizet, J.; Maveyraud, L.; Rengel, D.; Guillet, V.; Publicola, G.; Rodriguez, F.; Lherbet, C.; Mourey, L. Is Mycobacterial InhA a Suitable Target for Rational Drug Design? *ChemMedChem* **2025**, *20* (13). https://doi.org/10.1002/cmdc.202500079.

(4) Chollet, A.; Maveyraud, L.; Lherbet, C.; Bernardes-Génisson, V. An Overview on Crystal Structures of InhA Protein: Apo-Form, in Complex with Its Natural Ligands and Inhibitors. *Eur. J. Med. Chem.* **2018**, *146* (April 2019), 318–343. https://doi.org/10.1016/j.ejmech.2018.01.047.
